# Light-controlled enzymatic synthesis of γ-CD using a recyclable azobenzene template†

**DOI:** 10.1039/d3sc01997g

**Published:** 2023-06-29

**Authors:** Juliane Sørensen, Emilie Ljungberg Hansen, Dennis Larsen, Mathias Albert Elmquist, Andreas Buchleithner, Luca Florean, Sophie R. Beeren

**Affiliations:** a Department of Chemistry, Technical University of Denmark Kemitorvet Building 207 Kongens Lyngby 2800 Denmark sopbee@kemi.dtu.dk

## Abstract

Cyclodextrins (CDs) are important molecular hosts for hydrophobic guests in water and extensively employed in the pharmaceutical, food and cosmetic industries to encapsulate drugs, flavours and aromas. Compared with α- and β-CD, the wide-scale use of γ-CD is currently limited due to costly production processes. We show how the yield of γ-CD in the enzymatic synthesis of CDs can be increased 5-fold by adding a tetra-*ortho*-isopropoxy-substituted azobenzene template irradiated at 625 nm (to obtain the *cis*-(*Z*)-isomer) to direct the synthesis. Following the enzymatic reaction, the template can then be readily recovered from the product mixture for use in subsequent reaction cycles. Heating induces thermal *cis*-(*Z*) to *trans*-(*E*) relaxation and consequent dissociation from γ-CD whereupon the template can then be precipitated by acidification. For this study we designed and synthesised a set of three water-soluble azobenzene templates with different *ortho*-substituents and characterised their photoswitching behaviour using UV/vis and NMR spectroscopy. The templates were tested in cyclodextrin glucanotransferase-mediated dynamic combinatorial libraries (DCLs) of cyclodextrins while irradiating at different wavelengths to control the *cis*/*trans* ratios. To rationalise the behaviour of the DCLs, NMR titrations were carried out to investigate the binding interactions between α-, β- and γ-CD and the *cis* and *trans* isomers of each template.

## Abstract 2

A water-soluble, tetra-*ortho*-isopropoxy-substituted azobenzene is employed as a photo-removable and recyclable template for the enzymatic conversion of α-cyclodextrin to γ-cyclodextrin.

## Graphical abstract

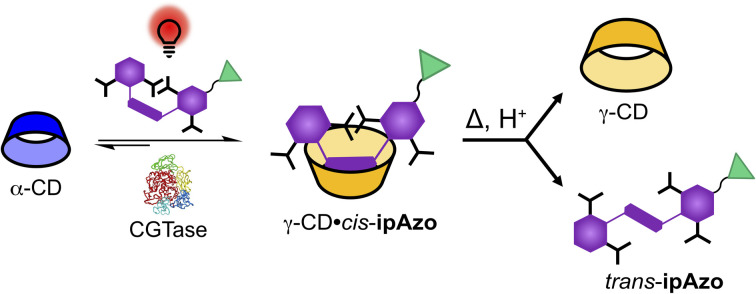

## Introduction

Cyclodextrins (CDs) are macrocyclic α-1,4-linked glucans naturally formed by bacterial digestion of starch. They are most commonly made up of 6, 7, or 8 glucopyranose units and named α-, β-, and γ-cyclodextrin, respectively.^1^ CDs have a truncated cone-like shape with a hydrophobic interior and hydrophilic exterior, which makes them ideal hosts for many different hydrophobic guests in water.^2^ CDs are industrially produced on a ton scale yearly and are used in the pharmaceutical, food, and cosmetic industries to encapsulate, solubilise and/or stabilise drug molecules, fat-soluble vitamins, fatty acids, aromas, and fragrances.^1,3^ While β-CD is the most commonly used CD, and makes up 70% of the global CD production, it is the least soluble, which limits some applications.^4^ γ-CD is the most soluble of the three CDs, and given its larger hydrophobic cavity and non-toxic profile, it has a great application potential, especially in the pharmaceutical industry.^5^ However, the use of γ-CD is limited by lower accessibility, due to costly and energy-inefficient production processes, and it constitutes only 5% of global CD production.^6^ Novel approaches to the production of γ-CD, leading to improved manufacturing processes, could enable the wider application of γ-CD in many industries.

We have shown that when cyclodextrin glucanotransferase (CGTase) acts on an α-1,4-glucan source, an interconverting mixture of CDs and linear α-1,4-glucans is formed.^7^ This system can be considered as a dynamic combinatorial library (DCL), akin to the more widely known dynamic systems based on reversible covalent reactions, such as disulfide exchange, hydrazone exchange and imine exchange.^8^ CGTase catalyses both fast, reversible transglycosylation and slow hydrolysis of α-1,4-glucosidic linkages. When an α-1,4-glucan is treated with CGTase, glucose is ultimately produced as the thermodynamic product.^9^ However, the CDs are intrinsically more stable than the linear glucans, and so form a kinetically trapped subsystem, which operates under *pseudo*-thermodynamic control, wherein the product distribution reflects the relative stabilities of the CDs.^7^ The distribution of CDs can be altered by adding a template molecule that selectively binds to, stabilises and amplifies a specific CD.^10^

Azobenzenes are amongst the best characterised photoswitches,^11^ and have been extensively explored for wide-ranging applications, such as photo-responsive polymers,^12^ molecular switches and machines,^13^ and functional materials for biomedical applications.^14^ Variation of the phenyl substituents can change the wavelength required for photoswitching, the thermal relaxation rates and binding capabilities.^15^ The complexation of azobenzenes with different CDs can be controlled with light, as different affinities and selectivities are observed for the *cis*-(*Z*) and *trans*-(*E*) isomers.^16^

We have previously reported the use of azobenzene templates in our CGTase-mediated DCLs of CDs to control the outcome of the enzymatic reaction with light.^17^ Using simple, unsubstituted azobenzene templates, the distribution could be changed towards the selective formation of either α- or β-CD just by irradiating with different wavelengths. With the aim of broadening the scope to also include light-controlled synthesis of γ-CD, tetra-*ortho*-substituted azobenzenes came to our attention. Wu and co-workers have investigated the binding of differently tetra-*ortho*-substituted azobenzenes to α-, β-, and γ-CD.^18^ In particular, the authors reported that tetra-*ortho*-isopropoxy-substituted azobenzene binds to γ-CD, but only as the *cis*-isomer.

**Fig. 1:**
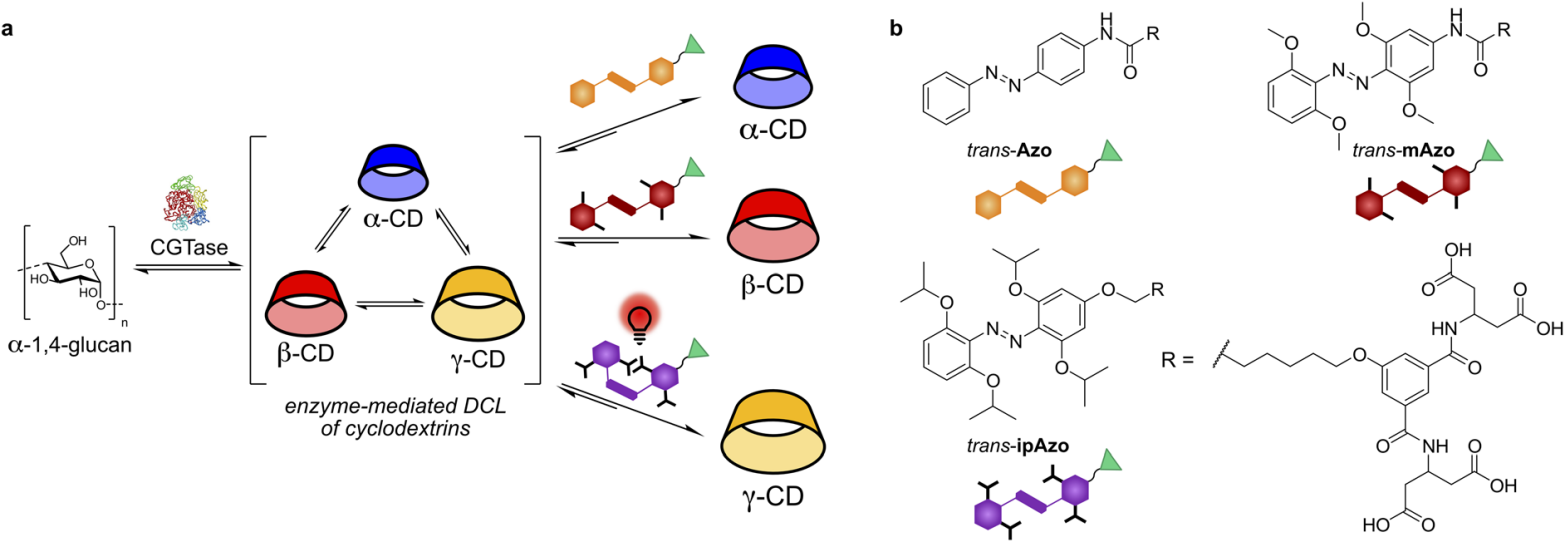
(a) Schematic representation showing CGTase acting on an α-1,4-glucan source to form a dynamic combinatorial library (DCL) of CDs and the use of different azobenzene templates and light irradiation to achieve selective synthesis of different CDs. The conditions that amplify α-, β- and γ-CD are shown. (b) Structures of the *trans* isomers of unsubstituted (Azo), methoxy-substituted (mAzo) and isopropoxy-substituted (ipAzo) azobenzenes synthesised in this work.

Herein, we present a set of water-soluble, photoswitchable templates for light-controlled enzymatic synthesis of CDs. We show that depending on the *ortho*-substituents, and the wavelength of light used to irradiate the DCL, we can favour the synthesis of α-, β-, and γ-CDs (Fig. 1). Using the isopropoxy-substituted template (ipAzo), we can obtain γ-CD in vastly improved yield, compared with the untemplated library. Moreover, this template can be readily recovered from the reaction by thermal isomerisation followed by precipitation, enabling its recycling and re-use in subsequent enzymatic syntheses.

## Results and discussion

### Design and synthesis of azobenzene templates

**Scheme 1:**
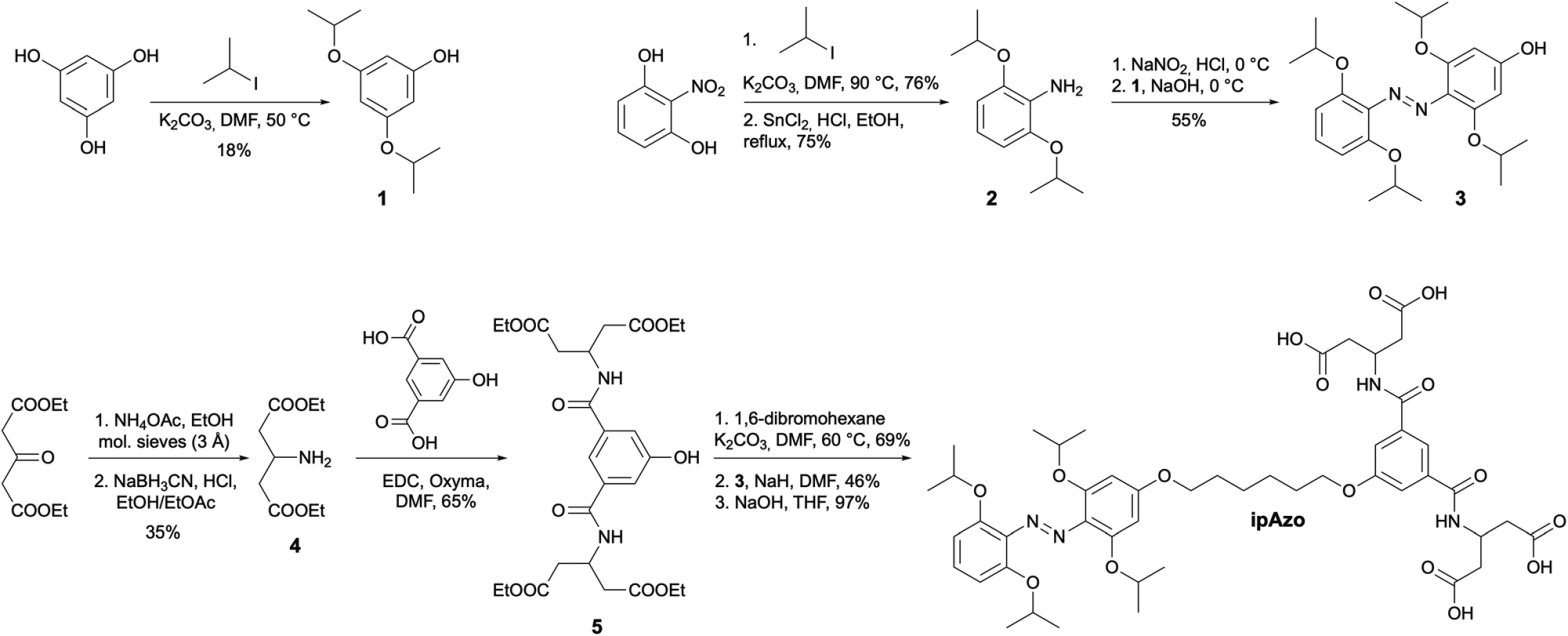
Synthesis of the tetra-*ortho*-isopropoxy-substituted azobenzene template ipAzo.

Three photoswitch templates incorporating azobenzene motifs with different *ortho*-substituents were synthesised: Azo, mAzo, and ipAzo, which feature no substituent, methoxy and isopropoxy substituents, respectively (Fig. 1b). To ensure suitable solubility at pH 7.5, the templates were appended with a hydrophilic tetracarboxylic acid motif *via* an aliphatic linker. Synthetic routes for the substituted azobenzene moieties were adapted from the procedure reported by Wu and co-workers.^18^ Synthetic schemes for the unsubstituted template (Azo) and methoxy-substituted template (mAzo) are shown in Schemes S1 and S2.† As a representative example, the synthetic route for the isopropoxy-substituted template (ipAzo) is shown in Scheme 1. Benzene-1,3,5-triol was treated with K_2_CO_3_ and 5 equivalents of 2-iodopropane at 50 °C to obtain 3,5-diisopropoxyphenol (1). 2-Nitroresorcinol was isopropylated using similar conditions, and the 2,6-diisopropoxynitrobenzene was then reduced to aniline 2 using SnCl_2_ and HCl in refluxing ethanol. Aniline 2 was treated with NaNO_2_ under acidic conditions at 0 °C to form the corresponding diazonium ion *in situ*, which was then reacted with phenol 1 to form azobenzene 3. The ester-protected solubilising group 5 ^10*a*^ was synthesised starting from diethyl 3-oxoglutarate, which was reacted with ammonium acetate in a reductive amination using sodium cyanoborohydride to obtain diethyl 3-aminoglutarate (4). 5-Hydroxyisophthalic acid was then coupled with 4 using EDC and oxyma to give the tetraester 5. The azobenzene and solubilising group were linked with an alkyl chain. 1,6-Dibromohexane was reacted with 5 using K_2_CO_3_ at 60 °C, then with 3 using NaH in dry DMF. Subsequent ester hydrolysis using aqueous NaOH in THF yielded template ipAzo. The solubility of the three templates were tested and pleasingly they were all soluble at least up to 10 mM in sodium phosphate buffer (100 mM) at pH 7.5.

### Characterisation of photophysical properties

**Fig. 2:**
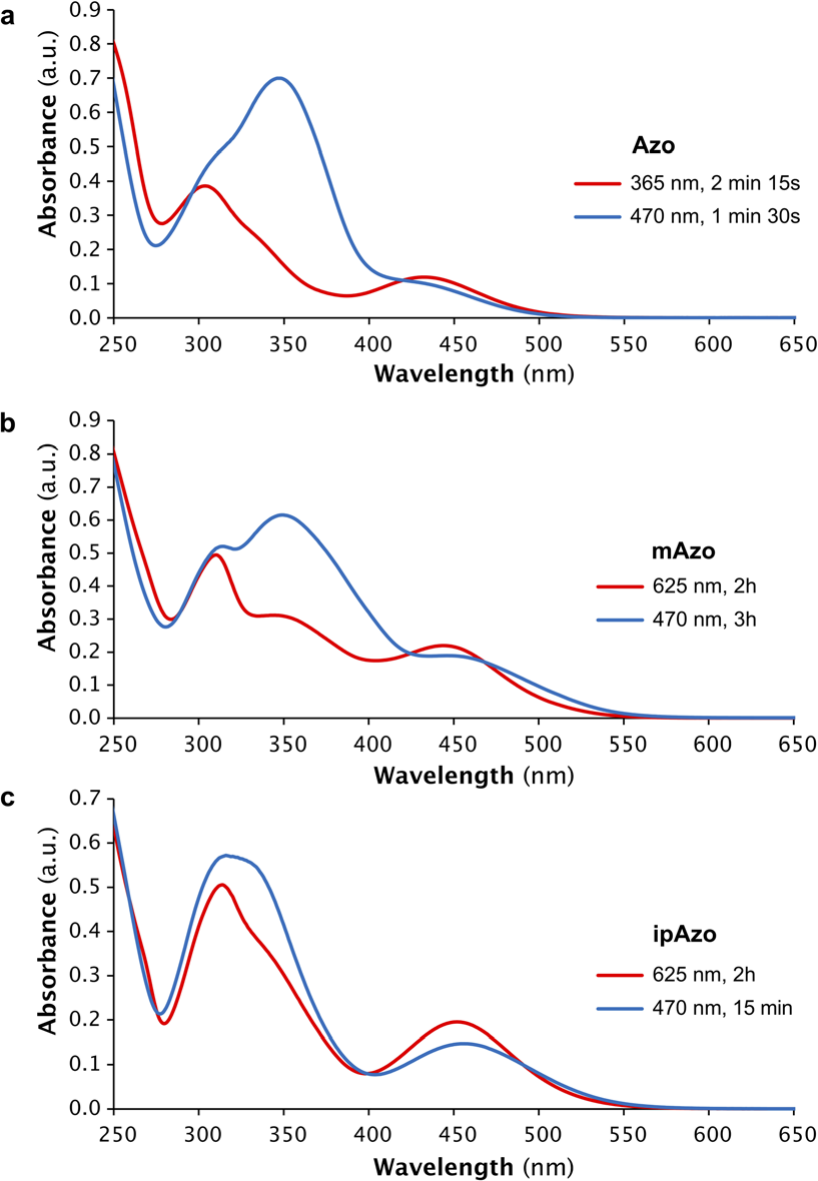
UV-vis absorption spectra of templates (a) Azo, (b) mAzo, and (c) ipAzo (50 μM in 100 mM sodium phosphate buffer at pH 7.5) showing the photostationary states at the specified wavelengths. The irradiation times required to obtain the different photostationary states are indicated. The spectra showing primarily the *cis* and *trans* isomers are depicted in red and blue, respectively.

The photophysical properties of the azobenzene templates were investigated using UV-vis absorption and NMR spectroscopy. Samples of Azo, mAzo and ipAzo were prepared in 100 mM sodium phosphate buffer at pH 7.5 (50 μM template for UV-vis spectroscopy and 10 mM for NMR spectroscopy). Irradiation of each photoswitch at 365 nm, 470 nm, 530 nm and 625 nm was examined (Fig. S1–S4†). The maximum *trans* to *cis* photoisomerisation was obtained by irradiating Azo with UV light at 365 nm and irradiating mAzo and ipAzo with red light at 625 nm, which resulted, in each case, in a decrease in the absorption band at around 350 nm and an increase in the absorption band near 450 nm (Fig. 2). For both mAzo and ipAzo, absorbance at 625 nm is very weak for the *trans* (as well as the *cis*) isomers, and irradiation for 2 hours was required to reach the photostationary state. Isomerisation from *cis* back to *trans* could in all cases be promoted by irradiation with blue light at 470 nm but this was less effective than thermal relaxation. After keeping the photo-irradiated solutions in the dark at 30 °C overnight, Azo, mAzo and ipAzo had thermally relaxed into 97%, 95% and 91% *trans*-isomer, as determined using ^1^H NMR spectroscopy (Fig. S5–S7†).

**Fig. 3:**
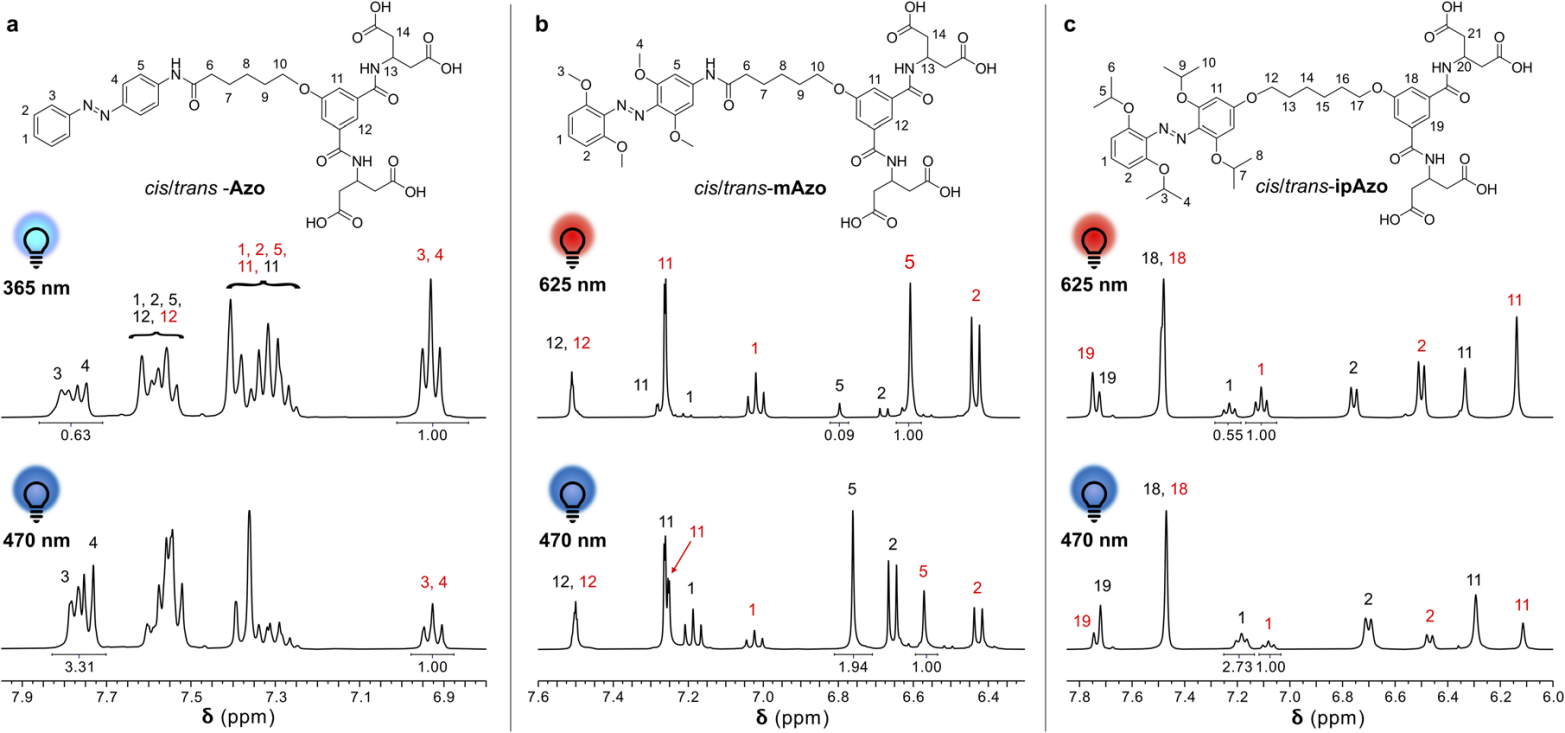
Partial ^1^H NMR spectra showing the aromatic region at the photostationary states for (a) Azo upon irradiation with UV light (365 nm) and blue light (470 nm); (b) mAzo upon irradiation with red light (625 nm) and blue light (470 nm); and (c) ipAzo when irradiated with red light (625 nm) and blue light (470 nm). Signals from the *cis* and *trans* isomers are shown labelled in red and black, respectively, and relative integrals are indicated for selected peaks.

The photostationary states upon irradiation with 365 nm, 530 nm, 625 nm and 470 nm were quantified using ^1^H NMR spectroscopy and are listed in Table 1. Fig. 3 depicts the aromatic region of the ^1^H NMR spectra of Azo, mAzo and ipAzo showing the photostationary states with highest observed *trans* and *cis* concentrations, respectively. *Trans*-Azo could be switched to 61% *cis*-Azo by irradiation at 365 nm. Meanwhile *trans*-mAzo could be switched to 92% *cis*-mAzo, and *trans*-ipAzo could be switched to 65% *cis*-ipAzo, by irradiation with red light at 625 nm. As all templates exhibited significantly different *cis*/*trans* ratios upon irradiation with light and after thermal back relaxation, they were judged suitable to be tested for light-controlled CD synthesis in CGTase-mediated DCLs.

**Table 1:** Photostationary states for Azo, mAzo, and ipAzo

	*Cis*-(*Z*) : *trans*-(*E*) ratio^a^				
	Before irr^b^	365 nm (UV)	530 nm (green)	625 nm (red)	470 nm (blue)
Azo	3 : 97	61 : 39	c	c	23 : 77
mAzo	5 : 95	c	c	92 : 8	34 : 66
ipAzo	9 : 91	c	50 : 50	65 : 35	24 : 76

a Conditions: 10 mM template at room temperature in 100 mM sodium phosphate buffer at pH 7.5. Determined using ^1^H NMR spectroscopy.

b Measured after storing at 30 °C in the dark overnight.

c Not determined.

### Enzyme-mediated dynamic combinatorial libraries

**Fig. 4:**
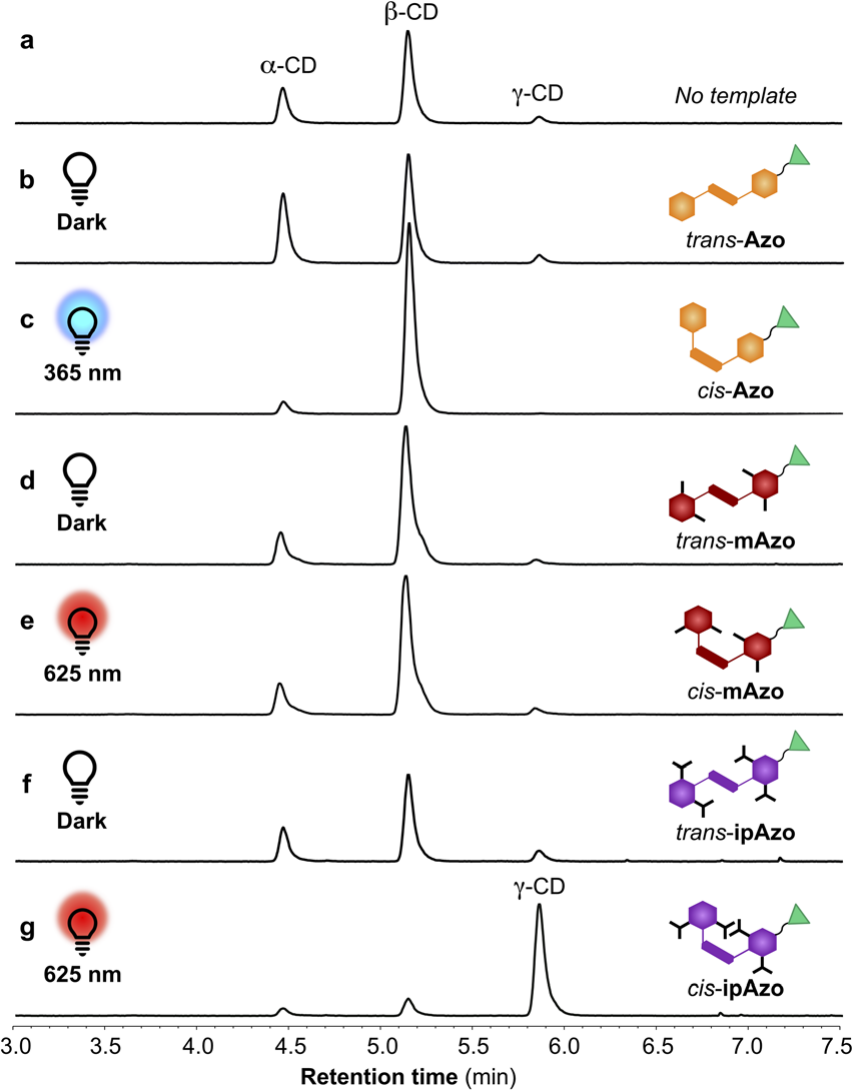
HPLC-ELSD chromatograms showing the relative concentrations of α-, β-, and γ-CD formed after 5 hours of reaction when CGTase acts on α-CD in the absence of template (a) and with templates Azo, mAzo, and ipAzo in darkness or under continuous irradiation with the indicated light (b–g). Cartoons of the templates show the major template isomer under the given conditions.

To explore the template effects of the isomers of Azo, mAzo, and ipAzo on a CGTase-mediated DCL of cyclodextrins, a series of libraries were set up. The DCLs were prepared by dissolving α-CD (10 mg mL^−1^) and the desired template (10 mM) in sodium phosphate buffered water (pH 7.5, 100 mM). Before starting the enzymatic reaction, these solutions were either kept in darkness overnight or irradiated with light (30 min at 365 nm for Azo, overnight at 625 nm for mAzo, and 1.5 hours at 625 nm for ipAzo). CGTase (65 μL stock solution pr mL reaction mixture) was then added, and irradiation (if any) was continued during the reaction. The library compositions were monitored over time using hydrophilic interaction liquid chromatography (HILIC) with an evaporative light scattering detector (ELSD) (Fig. S17–S20†). Fig. 4 shows the CD distributions obtained for the untemplated, templated and irradiated libraries after 5 hours when an approximately steady product mixture had been obtained. The untemplated libraries (both irradiated and non-irradiated) evolved in the same way to give a *pseudo*-equilibrium distribution of approximately 31% α-CD, 56% β-CD, and 13% γ-CD, calculated as % by weight of CDs present (Fig. 4a). In the presence of *trans*-Azo, a modest amplification of α-CD was observed, while a more significant amplification of β-CD was observed when the DCL was irradiated at 365 nm to generate *cis*-Azo (Fig. 4b and c). Both when mAzo was used as a template in the dark (*trans*-mAzo) and when irradiated at 625 nm (mostly *cis*-mAzo) only a small amplification of β-CD was observed compared to the untemplated library (Fig. 4d and e). While the *trans*-ipAzo influenced the DCL very little (Fig. 4f), when irradiated at 625 nm to generate the *cis*-ipAzo, a remarkable amplification of γ-CD was observed (Fig. 4g).

**Fig. 5:**
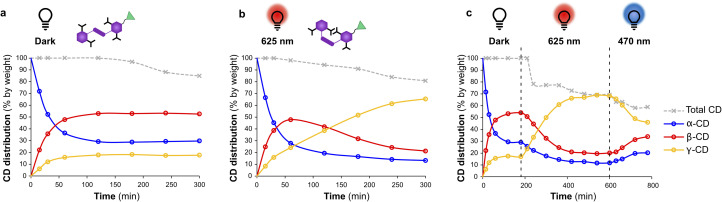
Distribution of α-, β-, and γ-CD as function of time for enzymatic reactions starting from α-CD (10 mg mL^−1^) with CGTase (65 μL mL^−1^ reaction mixture) in sodium phosphate buffer (100 mM, pH 7.5) in the presence of template ipAzo (10 mM). Reaction conditions: (a) in darkness having primarily the *trans* isomer, (b) under continuous red light irradiation (625 nm) promoting *trans* to *cis* isomerisation and (c) starting in darkness, then under irradiation with red light and finally under irradiation with blue light (470 nm) to promote *cis* to *trans* isomerisation.

Fig. 5 shows the changing product distribution in the DCLs templated with ipAzo overtime. When carried out in the dark, β-CD was obtained as the major CD product (50% selectivity) within 2 hours (Fig. 5a). A gradual decrease in total CD yield (grey line) is seen due to the build-up of short linear α-1,4-glucans.^7*a*^ In the DCL irradiated at 625 nm, β-CD accumulated during the first hour, but over the following hours the dynamic system adapted to produce primarily γ-CD, which reached 65% of the CD yield after 5 hours (Fig. 5b). The system was doubly dynamic and adaptive, as the product distribution could be switched back and forth by alternate irradiation with 625 nm and 470 nm light (Fig. 5c). An ipAzo-templated DCL was started in the dark and kept in darkness for 3 hours, then irradiated with red light at 625 nm for 7 hours to promote *trans* to *cis* isomerisation, and finally irradiated with blue light at 470 nm to favour *cis* to *trans* back-isomerisation. Initially α-CD and β-CD were the primary products when the template was *trans*-ipAzo. Then these were converted to γ-CD upon isomerisation of *trans*-ipAzo to *cis*-ipAzo. When the light irradiation was switched from red to blue, the yield of γ-CD decreased, and α-CD and β-CD concentrations increased again due to the conversion of *cis*-ipAzo to *trans*-ipAzo. Blue light does not induce complete *cis* to *trans* isomerisation of ipAzo (a 24 : 76 ratio of *cis* : *trans* is obtained in the photostationary state, see Table 1), and so conversion of γ-CD to α- and β-CD was reduced compared with the reaction in the dark, where the ratio of *cis* : *trans*ipAzo was 9 : 91.

### NMR binding studies

To understand the template-effects the DCLs under varying light conditions, binding studies with the isolated CDs were carried out using ^1^H NMR spectroscopy. The titrations were performed at low azobenzene concentrations (0.1–0.5 mM), as particularly the *trans* isomers were found to aggregate above this concentration (Fig. S8–S10†). The quantification of binding of photoswitches to a host is not straightforward if photoisomerisation does not give complete conversion between isomers or if thermal back relaxation is rapid. For the *trans* isomers of Azo, mAzo and ipAzo, sufficiently high ratios of the *trans* isomer (>90%) could be obtained by thermal back-isomerisation in the dark, such that association constants could be obtained from standard NMR titrations and fitting to a 1 : 1 binding isotherm. For the *cis* isomers, we used the methodology of simultaneous determination of association constants for a host to several guests in a mixture, which we have previously reported^19^ and applied to determine binding constants for photoswitches.^17^

Solutions of all three templates (in 100 mM sodium phosphate buffered D_2_O at pH 7.5) were, therefore, each titrated with α-CD, β-CD, and γ-CD under two different sets of conditions. The first set was performed in darkness after heating the template solutions at 30 °C overnight prior to the experiment to ensure maximum *cis* to *trans* thermal relaxation. Fitted binding isotherms for each *trans* isomer's binding to each CD are shown in Fig. S11–S13.† The second set of titrations was performed after irradiating Azo with UV light at 365 nm and mAzo and ipAzo with red light at 625 nm to obtain a mixture of *cis* and *trans* isomers (Fig. S14–S16†). The changes in chemical shifts for each isomer (Δ*δ*_(*cis*)_ and Δ*δ*_(*trans*)_) observed upon increasing the concentrations of CD were plotted against one another and fitted according to the equation below wherein *K*_a(*trans*)_ and Δ*δ*_max(*trans*)_ are known from the titrations performed in the dark, and *K*_a(*cis*)_ and Δ*δ*_max(*cis*)_ are determined from the fit.
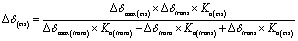
In a few cases, binding by one isomer was so weak, or its concentration so low in the mixture, that competitive binding could be ignored and a 1 : 1 fitting applied for the major isomer (see Section S4.3† for details). The calculated association constants are summarised in Table 2.

**Table 2:** Association constants, *K*_a_ (M^−1^), for *trans* and *cis* isomers of templates Azo, mAzo, and ipAzo with α-, β-, and γ-CD

	*K*_a_ a (M^−1^)		
	α-CD	β-CD	γ-CD
*Trans*-Azo	8100 ± 600	1800 ± 100	260 ± 30
*Cis*-Azo	500 ± 100	2800 ± 100	180 ± 20
*Trans*-mAzo	140 ± 40	420 ± 150	b
*Cis*-mAzo	50 ± 30	270 ± 100	b
*Trans*-ipAzo	170 ± 20	74 ± 9	b
*Cis*-ipAzo	33 ± 13	220 ± 80	2000 ± 300

a Determined at 25 °C in 100 mM sodium phosphate buffer in D_2_O at pH 7.5.

b The binding affinity was too weak to quantify.

Given these binding constants, the observed amplifications in the DCLs can be rationalised. As we have seen for a similar system,^17^ a small amplification of α-CD is observed for the DCL with Azo carried out in the dark, due to a stronger binding between *trans*-Azo and α-CD than with *trans*-Azo and β-CD. Meanwhile, upon irradiation with UV light, amplification of CD is observed due to a stronginteraction between *cis*-Azo and β-CD and only a weak interaction between *cis*-Azo and α-CD. The weak affinity of both isomers of mAzo for the CDs, and the only mild selectivity for β-CD, explain why only a small amplification of β-CD was observed in the DCLs in the presence of *trans*-mAzo or *cis*-mAzo. The dramatic amplification of γ-CD in the presence of ipAzo with irradiation at 625 nm is a consequence of a much stronger binding interaction occurring between *cis*-ipAzo and γ-CD compared with α- and β-CD. *Trans*-ipAzo does not bind to γ-CD, and so this amplification only occurs when the sample is irradiated.

### Template recovery and recycling

**Fig. 6:**
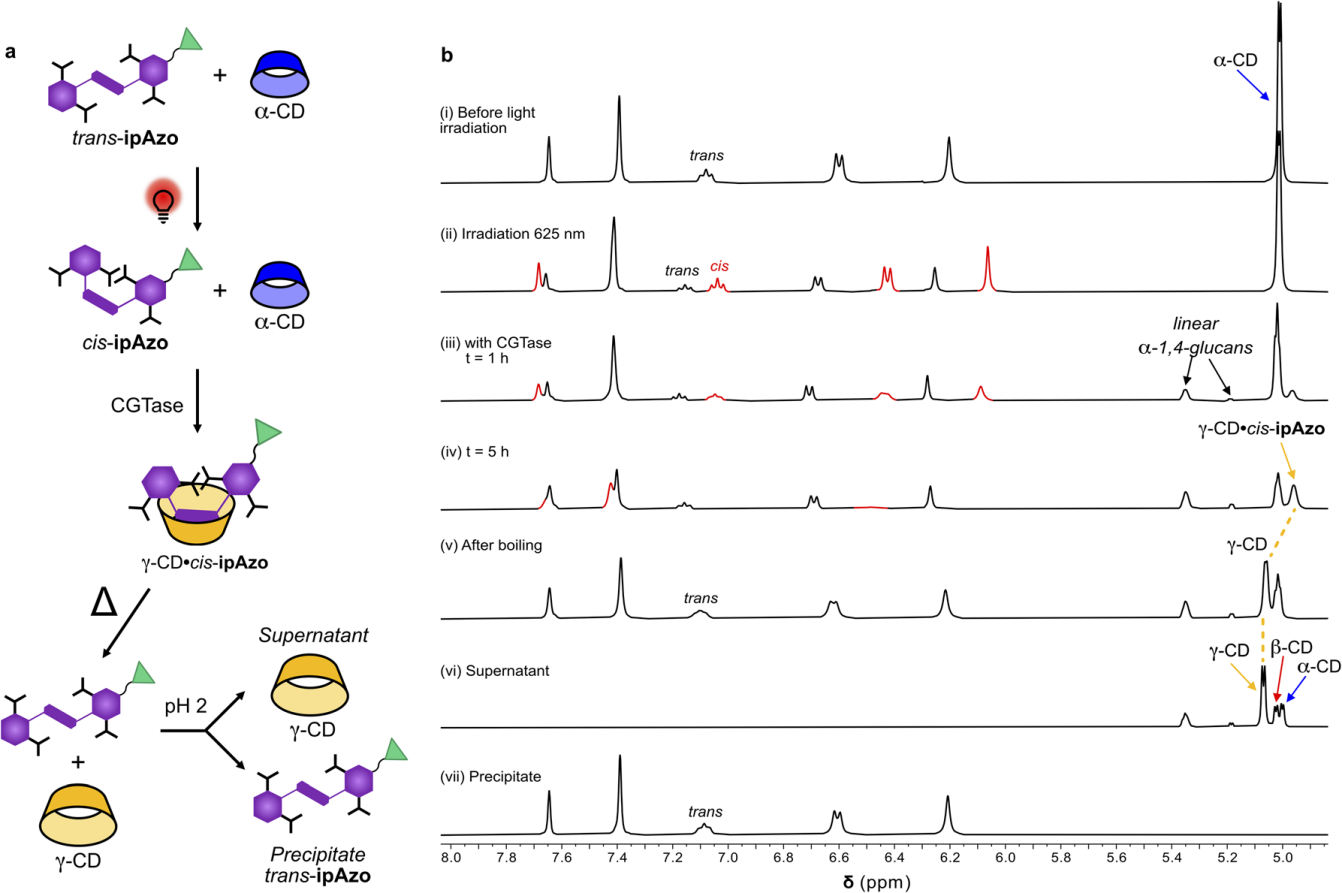
Templated enzymatic synthesis of γ-CD using *cis*-ipAzo and subsequent recovery of ipAzo. (a) Schematic representation of the workflow to synthesise γ-CD and re-isolate the template. (b) Partial ^1^H NMR spectra showing the aromatic protons of template ipAzo and the anomeric protons of the CDs before and after red light irradiation (i–ii), during enzymatic reaction (iii–iv), after boiling the reaction mixture to denature the enzyme, isomerise and unbind the template (v), and finally of the supernatant (vi) and precipitate (vii) after acidification.

For templated enzymatic synthesis to be of practical value for upscaled synthesis of cyclodextrins, the template should be recoverable and reusable. We have previously advocated the advantage of using a photoswitch as a template for synthesis of γ-CD, in that the light-induced dissociation of the template allows for easier isolation of the synthesised CD.^20^ipAzo has a further advantage, which is that it is a tetracarboxylic acid, and thus lends itself to precipitation and recovery upon acidification. We sought, therefore, to demonstrate, in a proof-of-principle NMR experiment, that ipAzo can not only be used to dramatically increase the yield of γ-CD in the enzymatic synthesis of CDs, it can also be easily separated from the CD products and recovered for further use (Fig. 6).

A solution of template ipAzo (10 mM) and α-CD (10 mg mL^−1^) in phosphate buffered D_2_O (100 mM) at pH 7.5 was irradiated at 625 nm overnight to obtain a mixture of *cis*- and *trans*-ipAzo (65 : 35) (Fig. 6b(ii)). The reaction was then started by addition of CGTase and monitored over time (Fig. 6b(iii–iv)). It was seen that the doublet due to the anomeric proton of α-CD (5.01 ppm) decreased and turned into an unresolved peak due to overlapping signals from the formed β-CD. At the same time, a broad signal (4.96 ppm) appeared corresponding to the anomeric signal of the γ-CD·*cis*-ipAzo complex. The signals from the aromatic protons of *cis*-ipAzo broadened and then disappeared, due to complexation with γ-CD occurring in an intermediate exchange regime on the NMR chemical shift timescale. Broadening of these aromatic signals upon complexation of *cis*-ipAzo with γ-CD was also observed in the titration (Fig. S16†). After 5 hours, the reaction was stopped by denaturing the enzyme with heat (95 °C for 15 min), which also led to thermal back-isomerisation of the template to obtain *trans*-ipAzo and unbound γ-CD as indicated by a downfield change in chemical shift of the anomeric signal from γ-CD (Fig. 6b(v)). The enzyme was removed by centrifugation and the supernatant was acidified by addition of trifluoroacetic acid (1% v/v) leading to immediate precipitation of a red solid. The precipitate was isolated and washed with water. Analysis of the precipitate and the supernatant, using ^1^H NMR spectroscopy (Fig. 6b(vi and vii)) and HPLC (Fig. S21†) showed that acidification is an efficient method to separate ipAzo from the CDs.

To further showcase the utility of ipAzo for templated enzymatic synthesis of γ-CD, the same template was used in five consecutive preparative–scale reaction cycles (starting from 23 mg α-CD and employing 22 mg of template). Each cycle was performed as just described. The five supernatants containing CDs and linear α-1,4-glucans were combined, and γ-CD was isolated by preparative HPLC in 32% yield (Fig. S22†). ipAzo was recovered with high purity (Fig. S23†) and in 93% yield after the five reaction cycles, thus demonstrating how this template can be readily recycled.

## Conclusions

In summary, we have shown that *ortho*-substituted azobenzenes can be used as photo-responsive templates to direct the selective synthesis of α-, β-, or γ-CD. In particular, when irradiated with red light, the template ipAzo caused a dramatic 5-fold increase in the yield of γ-CD compared to the untemplated reaction. Here, the observed amplification of γ-CD in the DCL confirmed the strong and selective interaction between the *cis* isomer of the tetra-*ortho*-isopropoxy-substituted azobenzene and γ-CD, as reported by Wu and co-workers^18^ and was in agreement with the binding affinities determined by our NMR titrations. Importantly, we showed that not only can ipAzo be used to amplify the production of γ-CD, but ipAzo also dissociates upon thermally-induced *cis*–*trans* isomerisation, and can then be precipitated by acidification allowing template recycling. We suggest that this photo-removable template strategy coupled with template recycling provides an industrially relevant scalable solution to address the current challenges in γ-CD production.

## Author contributions

DL and SRB conceptualised, designed and supervised the research. JS and ELH performed the experiments, including synthesis, characterisation, photoswitching and enzymatic reactions. MAE, AB and LF contributed to the development of the synthetic methodology. JS and SRB wrote the manuscript.

## Conflicts of interest

There are no conflicts to declare.

## Supplementary Material

SC-014-D3SC01997G-s001
