# Supplementary material for: Light-controlled enzymatic synthesis of γ-CD using a recyclable azobenzene template
Source: Chem Sci. 2023 Jun 29;14(28):7725–32. doi: 10.1039/d3sc01997g (PMC10355107; doi:10.1039/d3sc01997g)
Supplement: SC-014-D3SC01997G-s001 [file SC-014-D3SC01997G-s001.pdf]

Electronic Supplementary Material (ESI) for Chemical Science.
This journal is © The Royal Society of Chemistry 2023

Electronic supplementary information

Light-controlled enzymatic synthesis of γ-CD using a recyclable azobenzene template

Juliane Sørensen, Emilie Ljungberg Hansen, Dennis Larsen, Mathias Elmquist, Andreas Buchleithner, Luca Florean, and Sophie R. Beeren*

Contents

S1. Experimental details S2
S1.1 Materials S2
S1.2 Instruments S2
S2. Synthetic procedures S4
S2.1 Synthetic procedures towards template Azo S4
S2.2 Synthesis of template mAzo S7
S2.3 Synthesis of template ipAzo S10
S3. UV-vis experiments S14
S4. NMR experiments S16
S4.1 Determination of photostationary states S16
S4.2 Aggregation studies S19
S4.3 ^{1}H NMR titrations S22
S5. CGTase-mediated dynamic combinatorial libraries of cyclodextrins S31
S5.1 Sampling and analysis method S31
S5.2 DCL distributions obtained from HPLC chromatography S33
S5.3 Template recovery S34
S6. NMR spectra of synthesised compounds S37
S7. Supporting references S56

S1. Experimental details

S1.1 Materials

α-Cyclodextrin, buffer salts, deuterated NMR solvents and reagents used for synthesis of templates were purchased from commercial suppliers and used as received. Dry column vacuum chromatography (DCVC) was performed using silica gel 15–40 μm purchased from Merck. Flash column chromatography was performed using silica gel 40–63 μm purchased from Merck. Standard-Supercel-Z Celite® was used for dry loading compounds. HPLC grade solvents or better were used from commercial suppliers for all chromatographic analyses. Anhydrous solvents were obtained from an Innovative Technology PS-MD-7 Pure-solve solvent purification system. Molecular sieves (VWR Chemicals) were activated using microwave at 800 W for 4 × 1 minutes and subsequently dried under high vacuum. High purity water (Milli-Q) was obtained by filtering deionised water through a commercial water purification system (Merck Millipore Synergy UV) and used for the preparation of all aqueous solutions. Colourless Fisherbrand Microcentrifuge Tubes 0.6 mL were used for sample preparation (acidification and centrifugation) of HPLC samples, while colourless 2 mL glass vials with PTFE-lined screw-cap septa with a 0.2 mL glass insert were used for short term sample storage and injection on chromatographic equipment.

A stock solution of CGTase derived from *Bacillus macerans* was kindly gifted to our group by Amano Enzyme, Inc., Nagoya, Japan, and stored at 5 °C. According to specifications from the supplier, the stock solution contains approximately 20% glycerol, which was removed by performing a 160-fold solvent exchange with water using a Pall MicroSep Advance Centrifugal Device (0.5–5 ml) with a 10 kDa Omega Membrane according to the procedure described by the manufacturer. The final volume after solvent exchange was kept constant and the enzyme activity was tested.[S1] The glycerol-free stock solution of CGTase was used in all experiments except the preparative scale enzymatic synthesis of γ-CD, where the commercial enzyme solution was used without purification.

All reactions were monitored either by reversed-phased ultra-performance liquid chromatography mass spectrometry (RP-UPLC-MS) or by thin-layer chromatography (TLC) using Merck aluminium sheets covered with silica (C60). Visualisation of the TLC plates was conducted under UV light or by staining with a developing agent. All new compounds were characterised using NMR, RP-UPLC-MS (ESI), HRMS (ESI), and melting point where appropriate. NMR samples were measured in capped standard 5 mm borosilicate glass NMR tubes. Assignments of ^{1}H NMR spectra were achieved by the use of standard 2D NMR techniques: ^{1}H–^{1}H COSY, ^{1}H–^{13}C HSQC and ^{1}H–^{13}C HMBC.

S1.2 Instruments

HPLC chromatographic analysis was performed on a Thermo Scientific Dionex UltiMate 3000 HPLC (ultra-high pressure) system with a Waters Acquity UPLC BEH Amide 1.7 μm 2.1 × 150 mm column maintained at 30 °C. The system was equipped with an autosampler which was maintained at 20 °C. Injection volumes were 10 μL. For detection, the chromatographic system was connected to an Agilent Technologies 1260 Infinity ELSD, operating at evaporator and nebulizer temperatures of 90 and 70 °C, respectively, and an N_2 gas flow of 1.0 L/min.

NMR spectra were acquired on a Bruker Avance III 400 MHz NMR spectrometer equipped with Prodigy broadband observe (BBO) probe, except for ^{1}H NMR spectra acquired for the NMR titrations, which were acquired on a Bruker Avance III 400 MHz NMR spectrometer equipped with a BBO SmartProbe. Chemical shifts (δ) are quoted in ppm and coupling constants (*J*) are presented

in Hz. NMR spectra were referenced against residual solvent peaks. Measurements were performed at 298 K. When quantification of relative concentrations was required, ^{1}H NMR spectra were acquired using a delay of 10 seconds between pulses.

Analytical RP-UPLC-MS (ESI) analysis was performed on an S2 Waters AQUITY RP-UPLC system equipped with a diode array detector using a Thermo Accucore C18 column 2.6 µm, 2.1 x 50 mm; column temp: 50 °C; flow: 0.6 mL/min. Solvent A: 0.1% formic acid in water. Solvent B: 0.1% formic acid in MeCN. Gradient for short run: 5% B to 100% B in 2.4 min., hold 0.1 min., total run time 2.6 min. Gradient for long run: 5% B to 100% B in 3 min., hold 0.1 min., total run time 5 min. The LC system was coupled to an SQD mass spectrometer operating in both positive and negative electrospray modes. The temperature for all recordings was approximately 20 °C.

UV-vis absorption spectra were measured with a double beam Specord 210 Plus spectrometer from analytik jena. All measurements were performed in a 3.5 mL Hellma Quartz cuvette (labelled QS 282) with a 10 mm path length at room temperature.

ESI-HRMS were performed on a SolariX ESI FTMS spectrometer with dithranol as matrix. External calibration of the spectrometer was conducted with sodium trifluoroacetate cluster ions.

Melting points were measured on a Stuart SMP 30 melting point apparatus and are uncorrected. A Heraeus Biofuge Pico centrifuge was used for the preparation of samples for HPLC analysis. An Eppendorf Centrifuge 5810R was used during the synthesis of the **mAzo** and **ipAzo** templates, and for the preparative scale synthesis of γ-CD. For experiments involving light irradiation, all the LEDs were of the brand ThorLabs and driven by an adjustable power supply (0-1.2 A), and they were operated at their highest permitted power input (as defined by the supplier). Unless stated otherwise, the LEDs were operated at 1000 mA for the nominal wavelengths of 625 nm, 530 nm, and 470 nm, and for the nominal wavelength of 365 nm the LEDs were operated at 700 mA.

S2. Synthetic procedures

S2.1 Synthetic procedures towards template Azo

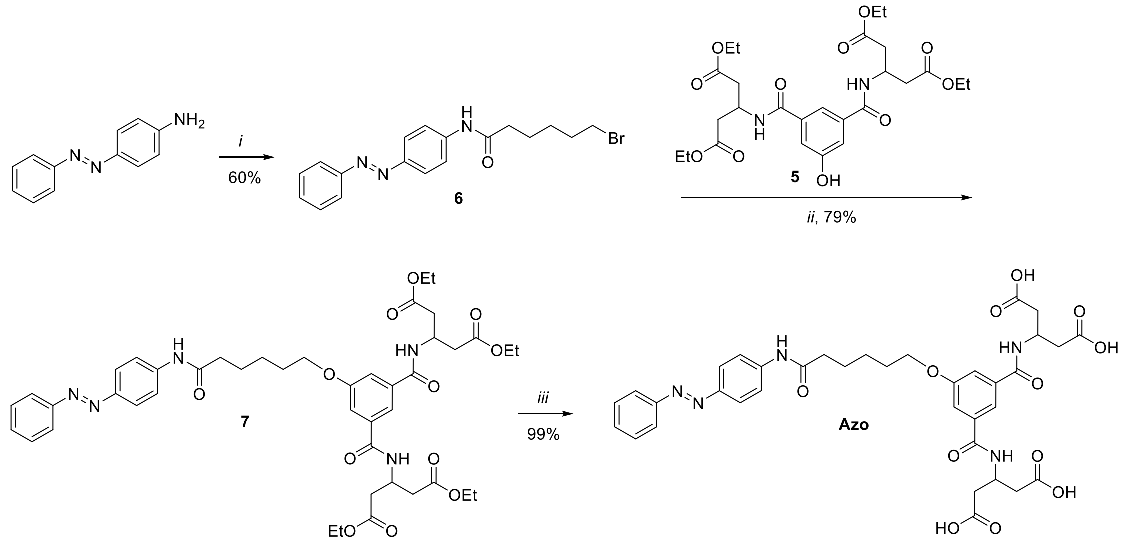

Scheme S1: Synthetic pathway towards template **Azo**. *i)* 6-Bromohexanoic acid, EDC, DMAP, CH_2Cl_2. *ii)* K_2CO_3, KI, 60 °C. *iii)* NaOH, THF, MeOH

Synthesis of compound **6**

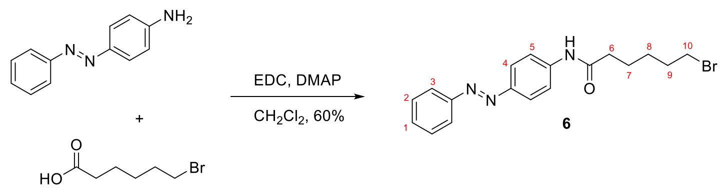

The procedure for the preparation of compound **6** was adapted from protocols reported by Wu et al.[S3] **Procedure:** 6-Bromohexanoic acid (1.99 g, 10.2 mmol), 4-phenyldiazenylaniline (2.05 g, 10.4 mmol), 1-(3-dimethylaminopropyl)-3-ethylcarbodiimide hydrochloride (EDC) (1.94 g, 10.1 mmol), and 4-dimethylaminopyridine (DMAP) (0.247 g, 2.02 mmol) were dissolved in CH_2Cl_2 (470 mL) resulting in an orange solution. The mixture was stirred at room temperature for 4 days. The solvent was removed *in vacuo* and the solid crude was purified by flash column chromatography (diameter: 7.5 cm, isocratic eluent: CH_2Cl_2) to yield the product as an orange solid (2.28 g, 6.09 mmol, 60%). **^{1}H NMR** (400 MHz, Chloroform-*d*) δ 7.96-7.88 (m, 4H, H3, H4), 7.69 (d, J = 8.6 Hz, 2H, H5), 7.54-7.43 (m, 4H, H1, H2), 7.37 (br. s, 1H, NH), 3.43 (t, J = 6.7 Hz, 2H, H10), 2.42 (t, J = 7.4 Hz, 2H, H6), 1.92 (tt, J = 6.7 Hz, J = 7.4 Hz, 2H, H9), 1.79 (tt, J = 7.7 Hz, J = 7.4 Hz, 2H, H7), 1.55 (m, 2H, H8). *Data in agreement with previously published data.*[S3]

Synthesis of compound **4**

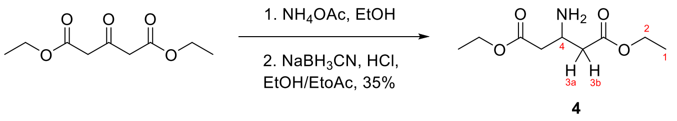

The experimental procedure was inspired by procedures reported by York et al.[S4] and Erichsen et al.[S1]

Procedure: To a solution of diethyl 3-oxoglutarate (20.03 g, 0.10 mol) in absolute ethanol (350 mL) with activated molecular sieves (12.36 g, 3 Å) was added ammonium acetate (24 g, 0.38 mol). The

formed suspension was stirred for 22 hours and then $NaBH_3CN$ (7.16 g, 0.11 mol) and HCl in EtOH/EtOAc* (~ 1 M, 600 mL) were added, resulting in a suspension with pH ~ 3. The suspension was stirred for 1.5 hours, then filtered and the resulting filtrate was concentrated *in vacuo*. The resulting oil was dissolved in water (200 mL) and K_2CO_3 (20 g) was added. Then aqueous NaOH (2 M, 200 mL) was added to adjust the pH to > 13 and the aqueous phase was extracted with ethyl acetate (5 × 200 mL). The combined organic extracts were washed with brine (3 × 160 mL) and dried over Na_2SO_4, filtered and concentrated *in vacuo* to leave a crude yellow oil. The crude oil was purified by flash column chromatography (diameter: 8.5 cm, length: 15 cm, ~ 7.5 g of crude oil, isocratic eluent: MeOH : CH_2Cl_2, 5 : 95) to yield the product as a yellow oil (7.09 g, 0.034 mol, 35%). **^{1}H NMR** (400 MHz, Chloroform-*d*) δ 4.15 (q, *J* = 7.2 Hz, 4H, H2), 3.63 (tt, *J* = 8.0, 4.7 Hz, 1H, H4), 2.51 (dd, *J* = 15.8, 4.7 Hz, 2H, H3a/H3b), 2.40 (dd, *J* = 15.8, 8.0 Hz, 2H, H3a/H3b), 1.26 (t, *J* = 7.2 Hz, 6H, H1). **^{13}C NMR** (101 MHz, Chloroform-*d*) δ 171.9, 60.7, 45.4, 41.8, 14.3. **HRMS** (ESI+) *m/z*: $[M+H]^+$ calculated for $C_9H_{18}NO_4$ 204.1231; found 204.1235. *Data in agreement with previously published data.*[S1,S4]

* Note: The ~1 M solution of HCl in EtOH/EtOAc was made by the slow addition of acetyl chloride (60 mL) to a cooled (0 °C) solution of absolute ethanol (800 mL, from a newly opened bottle). The reaction was stirred at 0 °C for one hour and then at room temperature for two hours.

<u>Synthesis of compound **5**</u>

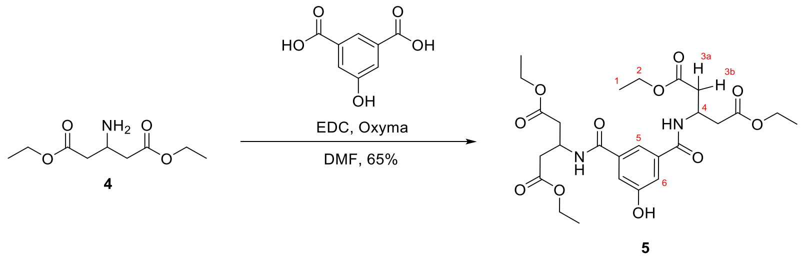

Procedure: To a solution of diethyl 3-aminoglutarate (**4**) (4.70 g, 23.0 mmol) and 5-hydroxyisophthalic acid (2.04 g, 11.0 mmol) in DMF (120 mL) was added ethyl 2-cyano-2-(hydroxyimino)acetate (Oxyma) (3.31 g, 23.0 mmol) and EDC (4.41 g, 23.0 mmol). The solution was left stirring at room temperature for 24 hours, after which the reaction mixture was diluted with water (600 mL) and extracted with EtOAc (5 × 160 mL). The combined organic phases were washed with aqueous HCl (1 M, 200 mL), aqueous saturated $NaHCO_3$ (4 × 160 mL) and brine (3 × 160 mL) before the combined organic phases were dried over $MgSO_4$, filtered and concentrated *in vacuo* to remove excess solvent. The crude product was purified by DCVC on silica gel (diameter: 6.5 cm, fraction volume: 100 mL, crude product adsorbed on Celite®, gradient elution from 0%–100% ethyl acetate in heptane in 7.5% increments then isocratic elution with ethyl acetate). Fractions 11-24 were collected, and the solvent removed *in vacuo* to yield the product as a white solid (4.13 g, 7.47 mmol, 65%). **M.P.:** 113.7 - 114.0 °C. **^{1}H NMR** (400MHz, Chloroform-*d*) δ 7.53 (t, *J* = 1.4 Hz, 1H, H5), 7.48 (d, *J* = 8.5 Hz, 2H, NH), 7.39 (d, *J* = 1.4 Hz, 2H, H6), 4.82 (dtt, *J* = 8.5, 6.1, 6.1 Hz, 2H, H4), 4.15 (q, *J* = 7.1 Hz, 8H, H2), 2.82 (dd, *J* = 16.1, 6.1 Hz, 4H, H3a/H3b), 2.78 (dd, *J* = 16.1, 6.1 Hz, 4H, H3a/H3b), 1.24 (t, *J* = 7.1 Hz, 12H, H1). **^{13}C NMR** (101 MHz, Chloroform-*d*) δ 171.5, 166.5, 157.5, 135.9, 117.7, 116.8, 61.1, 44.0, 38.1, 14.3. **HRMS** (ESI+) *m/z*: $[M+H]^+$ calculated for $C_{26}H_{37}N_2O_{11}$ 553.2392; found 553.2416.

Synthesis of compound **7**

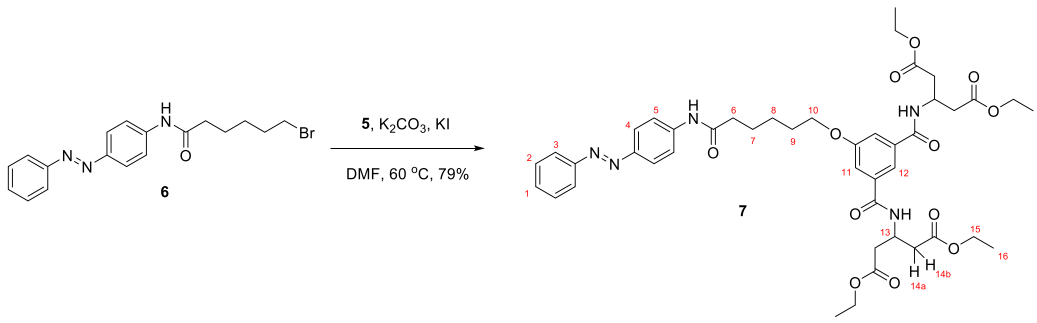

Procedure: Compound **6** (300 mg, 0.802 mmol) and compound **5** (478 mg, 0.865 mmol) were dissolved in dry DMF (10 mL) resulting in an orange solution. Oven dried K_2CO_3 (277 mg, 2.00 mmol) and a catalytic amount of KI (63 mg, 0.38 mmol) were added, and the reaction mixture was stirred at 60 °C for 22 hours. The black mixture was concentrated *in vacuo* to a few mL. The residue was diluted with water (10 mL) and extracted with ethyl acetate (15 mL then 3 × 10 mL). The combined organic extracts were successively washed with 1 M NaOH (10 mL), 0.1 M HCl (10 mL), and brine (10 mL), then dried over $MgSO_4$, filtered, and concentrated *in vacuo*. The crude product was purified by flash column chromatography (diameter: 3 cm, isocratic eluent: heptane/EtOAc, 1:2) to obtain the product as a pale orange solid (535 mg, 0.632 mmol, 79%). **M.P.**: 113.1 - 114.5 °C. **^{1}H NMR** (400 MHz, Chloroform-*d*) δ 8.06 (br s, 1H, NH), 7.90 (m, 4H, H3, H4), 7.76 (br d, *J* = 8.7 Hz, 2H, H5), 7.67 (t, *J* = 1.4 Hz, 1H, H12), 7.47 (m, 3H, H1, H2), 7.39 (d, *J* = 1.4 Hz, 2H, H11), 7.37 (d, *J* = 8.6 Hz, 2H, NH), 4.83 (dtt, *J* = 8.6, 6.2, 5.8 Hz, 2H, H13), 4.15 (q, *J* = 7.1 Hz, 8H, H15), 3.99 (t, *J* = 6.3 Hz, 2H, H10), 2.82 (dd, *J* = 16.0, 5.8 Hz, 4H, H14a/H14b), 2.73 (dd, *J* = 16.0, 6.2 Hz, 4H, H14a/H14b), 2.45 (t, *J* = 7.5 Hz, 2H, H6), 1.80 (m, 4H, H7, H9), 1.54 (m, 2H, H8), 1.25 (t, *J* = 7.1 Hz, 12H, H16). **^{13}C NMR** (101 MHz, Chloroform-*d*) δ 171.8, 171.4, 165.9, 159.4, 152.7, 148.9, 141.2, 136.0, 130.9, 129.2, 124.2, 122.9, 119.9, 117.4, 116.4, 77.4, 68.1, 61.0, 43.9, 37.9, 28.6, 25.6, 25.2, 14.3. **HRMS** (ESI+) *m/z*: $[M+H]^+$ calculated for $C_{44}H_{56}N_5O_{12}$ 846.3920; found 846.3930.

Synthesis of template **Azo**

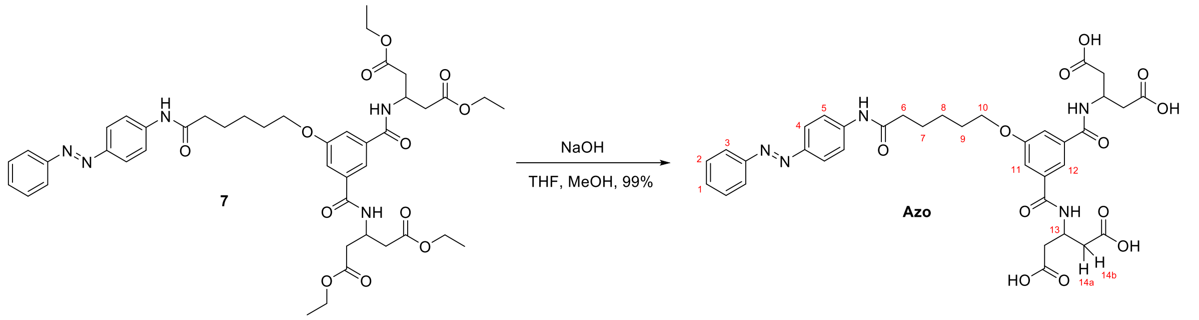

Procedure: Compound **7** (360 mg, 0.426 mmol) was dissolved in THF (25 mL) and MeOH (50 mL) to form an orange solution. Aqueous NaOH (1 M, 25 mL) was added, and the reaction mixture was stirred at room temperature for 2 hours. The solution was concentrated *in vacuo* until approximately 7 mL remained and 6 M HCl was added to adjust the pH to 1. The resulting precipitate was collected by suction filtration, washed successively with 0.1 M HCl and water, and dried under high vacuum to obtain the product as a red solid (310 mg, 422 mg, 99%). **M.P.**: 212.4 - 214.9 °C. **^{1}H NMR** (400 MHz, DMSO-d_6) δ 10.36 (br s, 1H, NH), 8.53 (d, *J* = 8.0 Hz, 2H, NH), 7.86 (m, 7H, H3, H4, H5, H12), 7.54 (m, 5H, H1, H2, H11), 4.58 (m, 2H, H13), 4.07 (t, *J* = 6.2 Hz, 2H, H10), 2.61 (dd, *J* = 15.8, 7.4 Hz, 4H, H14a/H14b), 2.53 (dd, *J* = 15.8, 6.4 Hz, 4H, H14a/H14b), 2.42 (t, *J* = 7.4 Hz, 2H,

H6), 1.75 (m, 4H, H7, H9), 1.51 (m, 2H, H8). **^{13}C NMR** (101 MHz, DMSO-d_6) δ 172.2, 171.8, 165.0, 158.3, 152.1, 147.6, 143.2, 136.0, 131.0, 129.4, 124.1, 122.3, 119.2, 118.9, 115.7, 67.8, 44.7, 38.1, 36.5, 28.5, 26.2, 24.8. **HRMS** (ESI+) *m/z*: $[M+H]^+$ calculated for $C_{36}H_{40}N_5O_{12}$ 732.2522; found 732.2536.

S2.2 Synthesis of template mAzo

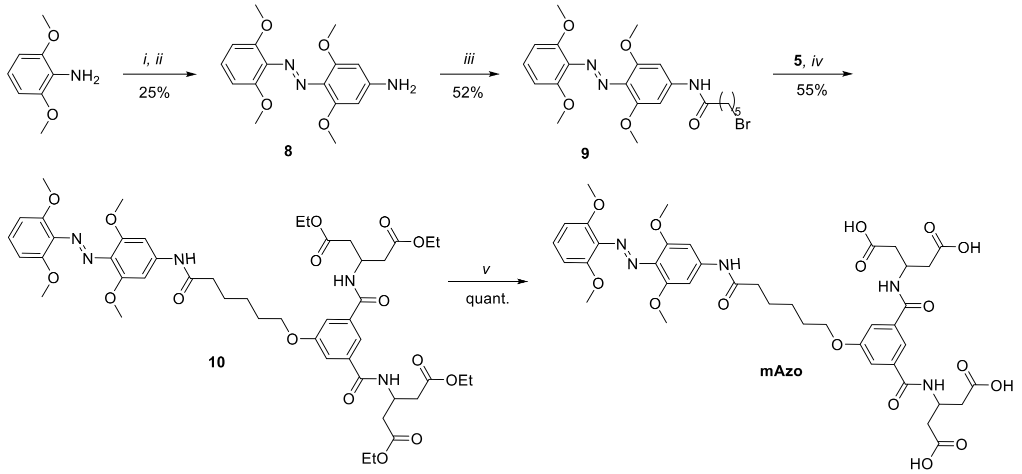

Scheme S2: Synthetic pathway towards **mAzo**. *i)* HCl, $NaNO_2$, 0 ºC. *ii)* 3,5-dimethoxy-aniline, K_2CO_3, 0 ºC. *iii)* 6-Bromohexanoic acid, EDC, DMAP, CH_2Cl_2. *iv)* K_2CO_3, KI, DMF, 60 ºC. *v*) NaOH (aq.), THF.

Synthesis of compound **8**

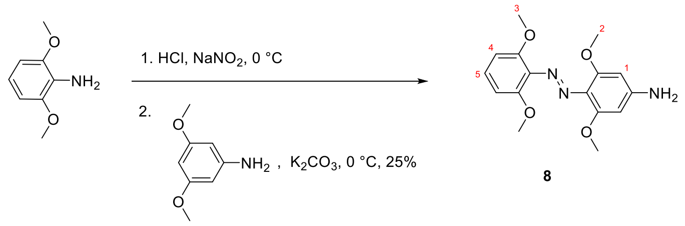

The procedure for the preparation of compound **8** was inspired by protocols reported by Wu et al.[S6] **Procedure:** 2,6-Dimethoxyaniline (0.543 g, 3.50 mmol) was added to a solution of H_2O (0.66 mL) and concentrated HCl (0.86 mL) resulting in a black suspension. The mixture was cooled to approximately 0 °C using an ice bath and $NaNO_2$ (0.241 g, 3.50 mmol) dissolved in H_2O (2.5 mL) was added dropwise. The mixture, consisting of black solids in a dark yellow solution, was stirred at approximately 0 °C for 25 minutes, during which the black solid dissolved. This solution was added dropwise to a suspension of 3,5-dimethoxyaniline (0.543 g, 3.50 mmol) in H_2O (20 mL) kept at ca. 0 °C, after which 3,5-dimethoxyaniline went into solution. The pH was adjusted to 8-9 by adding aqueous saturated $NaHCO_3$ and a red solid precipitated. The mixture was stirred overnight at room temperature. The crude slurry was filtered with suction and the precipitate was collected. The solid was dissolved in ethyl acetate, and purified by flash column chromatography (diameter: 4.5 cm, length: 10 cm, gradient elution from 50%–80% methanol in ethyl acetate) to yield the product as a red solid (0.230 g, 0.720 mmol, 25%). **M.P.**: 193.0 - 196.2 °C. **^{1}H NMR** (400 MHz, DMSO-d_6) δ 7.13 (t, 1H, J = 8.4 Hz, H5), 6.70 (d, J = 8.4 Hz, 2H, H4), 5.98 (br s, 2H, NH_2), 5.93 (s, 2H, H1), 3.66 (s, 6H, H2/H3), 3.65 (s, 6H, H2/H3). *Data in agreement with previously published data.*[S6]

Synthesis of compound **9**

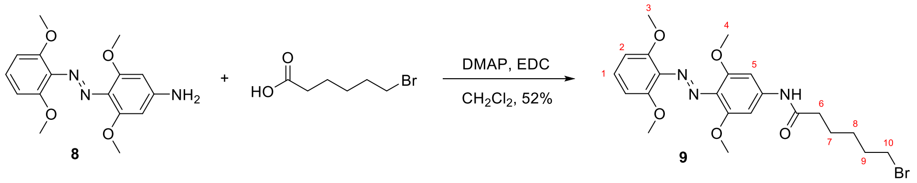

The procedure for the preparation of compound **9** was inspired by protocols reported by Wu et al.[S6]
Procedure: 6-Bromohexanoic acid (0.153 g, 0.780 mmol), EDC (0.170 g, 0.890 mmol) and DMAP (0.0147 g, 0.120 mmol) was dissolved in CH_2Cl_2 (37 mL). The colourless solution was stirred at room temperature for 30 min. Compound **8** (0.260 g, 0.820 mmol) was added and the solution turned dark red. The solution was stirred overnight at room temperature, then diluted with CH_2Cl_2 (50 mL) and washed with aqueous HCl (0.1 M, 4 × 60 mL) and aqueous saturated $NaHCO_3$ (3 × 60 mL). The organic phase was dried over $MgSO_4$ and filtered. The filtrate was concentrated onto Celite® and purified by flash column chromatography (diameter: 2.5 cm, length: 10 cm, gradient elution from 1:1 heptane/ethyl acetate to 100% ethyl acetate) to yield the product as a black solid (0.198 g, 0.400 mmol, 52%). **M.P.**: > 400 °C. **^{1}H NMR** (400 MHz, DMSO-d_6) δ 10.13 (br s, 1H, NH (trans)), 9.87 (br s, 1H, NH (cis)), 7.26 (t, J = 8.2 Hz, 1H, H1 (trans)), 7.15 (s, 2H, H5 (trans)), 7.06 (t, J = 8.2 Hz, 1H, H1 (cis)), 6.86 (s, 2H, H5 (cis)), 6.77 (d, J = 8.2 Hz, 2H, H2 (trans)), 6.54 (d, J = 8.2 Hz, 2H, H2 (cis)), 3.71 (2 s, 12H, H3, H4 (trans)), 3.56 (t, J = 6.7 Hz, 4H, H10), 3.54 (s, 6H, H3/H4 (cis)), 3.48 (s, 6H, H3/H4 (cis)), 2.36 (t, J = 7.5 Hz, 2H, H6 (trans)), 2.27 (t, J = 7.5 Hz, 2H, H6 (cis)), 1.84 (m, 4H, H9), 1.62 (m, 4H, H7), 1.43 (m, 4H, H8). *Data in agreement with previously published data.*[S6]

Synthesis of compound **10**

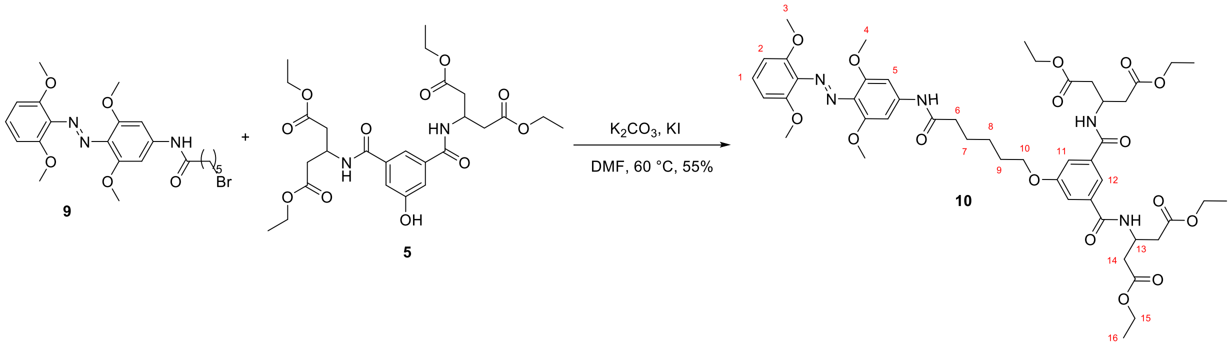

Procedure: Compound **9** (1.37 mg, 0.28 mmol) and compound **5** (0.169 g, 0.310 mmol) were dissolved in DMF (4.5 mL). Oven dried K_2CO_3 (96 mg, 0.70 mmol) was added to the solution. KI (46 mg, 0.28 mmol) was added and the solution was heated to 60 °C and the red solution was stirred for 24 hours. The solution was then concentrated *in vacuo* until ca. 0.5 mL solvent remained. Water (20 mL) was added, and the resulting aqueous solution was extracted with CH_2Cl_2 (2 × 20 mL, then 1 × 10 mL). The combined organic phases were washed with aqueous NaOH (1 M, 2 × 10 mL), aqueous HCl (0.2 M, 20 mL) and brine (10 mL). The organic solution was dried over Na_2SO_4, filtered, and evaporated *in vacuo* to yield the crude product. The crude product was purified using flash column chromatography eluting with a gradient from 0%–3% MeOH in CH_2Cl_2, to yield the product as a red solid (149 mg, 154 mmol, 55%). **M.P.**: 69.1 - 71.2 °C. **^{1}H NMR** (400 MHz, DMSO-d_6) δ 10.16 (br s, 1H, NH (trans)), 9.89 (br s, 1H, NH (cis)), 8.54 (d, J = 8.0 Hz, 4H, NH), 7.82 (t, J = 1.2 Hz, 2H, H12), 7.47 (d, J = 1.2 Hz, 2H, H11 (trans)), 7.44 (d, J = 1.2 Hz, 2H, H11 (cis)), 7.24 (t, J = 8.4 Hz, 1H, H1 (trans)), 7.15 (s, 2H, H5 (trans)), 7.05 (t, J = 8.4 Hz, 1H, H1 (cis)), 6.87 (s, 2H, H5 (cis)), 6.76 (d, J = 8.4 Hz, 2H, H2 (trans)), 6.55 (d, J = 8.4 Hz, 2H, H2 (cis)), 4.65 (m, 4H, H13),

4.10-3.99 (m, 20H, H10, H15), 3.73-3.69 (2 s, 12H, H3, H4 (trans)), 3.53 (s, 6H, H3/H4 (cis)), 3.48 (s, 6H, H3/H4 (cis)), 2.63 (m, 16H, H14), 2.39 (t, $J = 7.2$ Hz, 2H, H6 (trans)), 2.30 (t, $J = 7.2$ Hz, 2H, H6 (cis)), 1.84-1.63 (m, 8H, H9, H7), 1.51 (m, 4H, H8), 1.12 (t, $J = 7.2$ Hz, 24H, H16). **^{13}C NMR** (101 MHz, DMSO-d_6) δ 172.3, 170.9, 165.5, 158.8, 153.5, 151.7, 142.2, 136.4, 134.5, 134.4, 129.0, 119.2, 116.1, 105.8, 96.1, 68.4, 60.5, 56.5, 56.4, 44.5, 39.0, 37.0, 28.9, 25.7, 25.6, 14.5.* **HRMS** (ESI+) *m/z*: $[M+H]^+$ calculated for $C_{48}H_{64}N_5O_{16}$ 966.4343; found 966.4349. *Note: Only signals from the *trans* isomer are listed, as determined from an ^{1}H–^{13}C HSQC experiment.

<u>Synthesis of template **mAzo**</u>

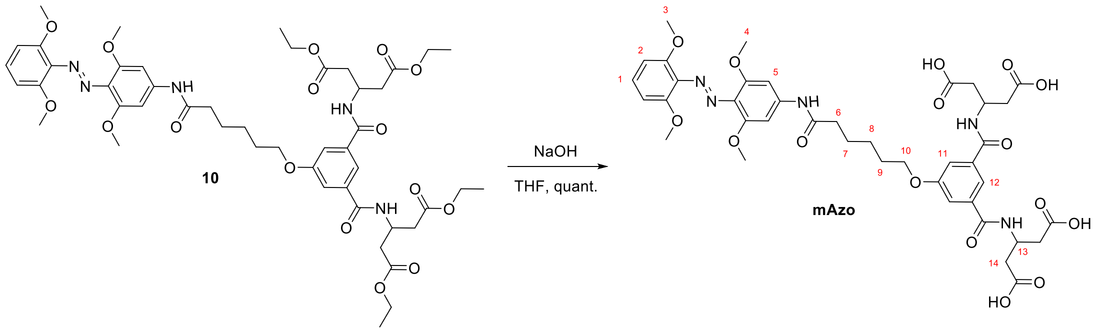

Procedure: Compound **10** (32 mg, 0.033 mmol) was dissolved in THF (3 mL) to form a dark red solution. Aqueous NaOH (1 M, 0.75 mL) was added and the solution was stirred 4 hours at room temperature. THF was evaporated under reduced pressure and aqueous HCl (6 M, 2 mL) was added after which a red precipitate immediately formed. The suspension was centrifuged at 12000 rpm, and the supernatant was discarded. The red precipitate was washed with water 2 times by suspending in 2 mL water and centrifuging at 12000 rpm. The water was discarded, and the precipitate was dried *in vacuo* to yield the product as a red solid (28 mg, 0.033 mmol, quant..). **M.P.**: 168.2–170.5 °C. **^{1}H NMR** (400 MHz, 100 mM sodium phosphate buffered D_2O at pH 7.5) δ 7.64 (m, 2H, H12), 7.42 (d, $J = 1.0$ Hz, 4H, H11), 7.38 (t, $J = 8.4$ Hz, 1H, H1 (trans)), 7.20 (t, $J = 8.4$ Hz, 1H, H1 (cis)), 6.93 (s, 2H, H5 (trans)), 6.84 (d, $J = 8.4$ Hz, 2H, H2 (trans)), 6.73 (s, 2H, H5 (cis)), 6.61 (d, $J = 8.4$ Hz, 2H, H2 (cis)), 4.64 (quint, $J = 6.9$ Hz, 4H, H13), 4.12 (t, $J = 6.4$ Hz, 4H, H10), 3.84 (2s, 12H, H4 (trans), H3 (trans)), 3.59 (s, 6H, H4 (cis)/H3 (cis)), 3.55 (s, 6H, H4 (cis)/H3 (cis)), 2.50 (m, 16H, H14), 2.44 (t, $J = 7.4$ Hz, 4H, H6), 1.86-1.69 (m, 8H, H9, H7), 1.51 (m, 4H, H8). **^{13}C NMR** (101 MHz, DMSO-d_6) δ 173.0, 171.7, 164.8, 159.1, 153.4, 151.1, 141.5, 136.4, 133.6, 128.5, 128.4, 118.6, 115.6, 105.2, 95.6, 67.7, 56.0, 55.8, 43.9, 38.3, 36.5, 28.4, 25.2, 24.6.* **HRMS** (ESI+) *m/z*: $[M+H]^+$ calculated for $C_{40}H_{48}N_5O_{16}$ 854.3091; found 854.3102.

*Note: Only signals from the trans isomer are listed, as determined from an ^{1}H–^{13}C HSQC experiment.

S2.3 Synthesis of template ipAzo

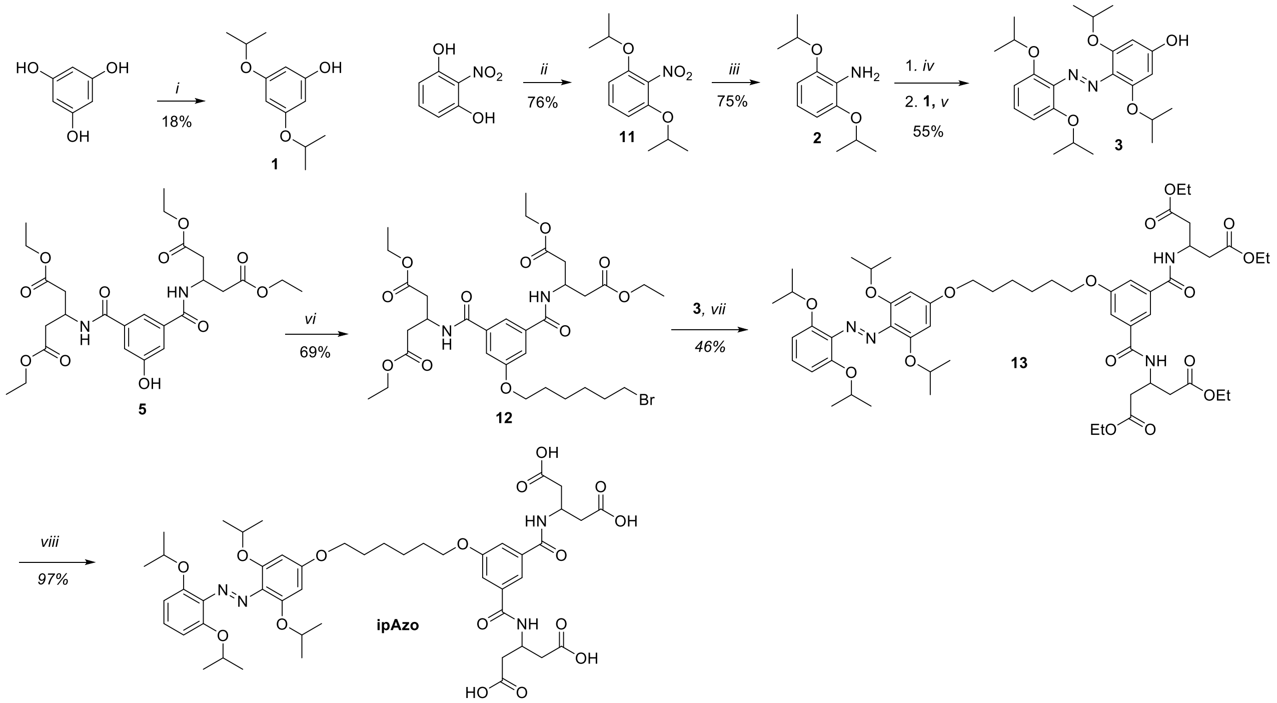

Scheme S3: Synthetic pathway for template **ipAzo**. *i)* 2-iodopropane, K_2CO_3, DMF, 50 ºC. *ii)* 2-iodopropane, K_2CO_3, DMF, 90 ºC. *iii)* $SnCl_2$, HCl, EtOH, reflux. *iv)* HCl, $NaNO_2$, 0 ºC. *v)* NaOH, 0 ºC, *vi)* 1,6-dibromohexane, K_2CO_3, DMF, 60 ºC. *vii*) NaH, DMF. *viii)* NaOH (aq.), THF.

Synthesis of compound **1**

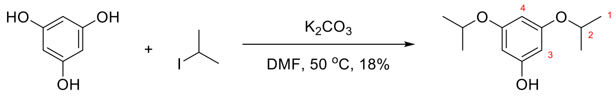

1

Procedure: To a suspension of benzene-1,3,5-triol (2.00 g, 15.9 mmol) and K_2CO_3 (11.0 g, 79.6 mmol) in DMF (100 mL) was added 2-iodopropane (6.35 mL, 63.5 mmol). The reaction mixture was heated to 50 °C and left to stir for 5 hours. The reaction mixture was cooled to room temperature, diluted with water (100 mL) and extracted with ethyl acetate (3 × 50 mL). The combined organic extracts were washed with brine (50 mL), dried with $MgSO_4$, filtered, and concentrated *in vacuo*. The brown, crude oil was purified by flash column chromatography (diameter: 4.5 cm, 1.3 g crude, gradient elution from 5%–30% ethyl acetate in heptane) to yield the product as a viscous, brown-tinted oil (0.606 g, 2.9 mmol, 18 %). **^{1}H NMR** (400 MHz, Chloroform-*d*) δ 6.04 (t, *J* = 2.0 Hz, 1H, H4), 5.98 (d, *J* = 2.0 Hz, 2H, H3), 4.46 (septet, *J* = 6.1 Hz, 2H, H2), 1.31 (d, *J* = 6.1 Hz, 12H, H1). *Data in agreement with previously published data.*[S3]

Synthesis of compound **11**

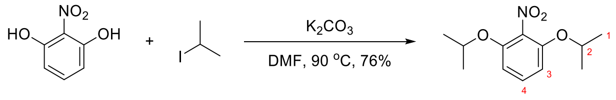

11

Procedure: To a solution of 2-nitroresorcinol (1.55 g, 9.99 mmol) and K_2CO_3 (4.15 g, 30.0 mmol) in DMF (100 mL) was added 2-iodopropane (2.3 mL, 23 mmol). The reddish black solution was heated to 90 °C and stirred at this temperature for 17 hours. The DMF was removed

in vacuo and H_2O (50 mL) was added to the residue. The aqueous phase was extracted with ethyl acetate (3 × 25 mL) and the combined organic extracts were dried with $MgSO_4$, filtered, and concentrated *in vacuo* to yield the crude as a brown oil. The crude was purified by DCVC (diameter: 4.0 cm, length: 7 cm, crude product adsorbed on Celite®, fraction volume: 50 mL, gradient elution from 0%–30% ethyl acetate in heptane in 3% increments). The product eluted in fractions 5-6, which were collected and the solvent was removed *in* vacuo to obtain the product as a pale yellow solid (1.81 g, 7.56 mmol, 76%). **M.P.**: 50.2 – 51.1 °C. **^{1}H NMR** (400 MHz, DMSO-d_6) δ 7.39 (t, J = 8.5 Hz, 1H, H4), 6.86 (d, J = 8.5 Hz, 2H, H3), 4.72 (septet, J = 6.1 Hz, 2H, H2), 1.23 (d, J = 6.1 Hz, 12H, H1). *Data in agreement with previously published data.*[S3]

Synthesis of compound **2**

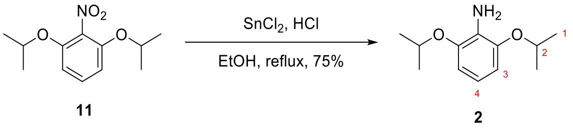

Procedure: 2,6-diisopropoxynitrobenzene (**11**) (1.48 g, 6.19 mmol) and $SnCl_2$ (11.7 g, 61.7 mmol) were dissolved in ethanol (160 mL) and aqueous HCl (3 M, 4.0 mL) was added. The white suspension was stirred at 85 °C under reflux for 23 hours. The reaction mixture was cooled to room temperature, and aqueous NaOH (3 M) was added to adjust the pH to 11. The white precipitate formed ($Sn(OH)_2$) was removed by suction filtration through a pad of compressed Celite®. The filtrate was extracted by CH_2Cl_2 (3 × 50 mL), and the combined organic extracts were dried with $MgSO_4$, filtered and concentrated *in vacuo*. The brown crude oil was purified by DCVC (diameter: 4.0 cm, crude product adsorbed on Celite®, fraction volume: 50 mL, gradient elution from 0%–10% ethyl acetate in heptane in 1% increments). Fractions 6-8 were collected and the solvent was removed *in vacuo* to obtain the product as a yellow-tinted liquid (0.97 g, 4.6 mmol, 75%). **^{1}H NMR** (400 MHz, DMSO-d_6) δ 6.49 (m, 3H, H3, H4), 4.46 (septet, J = 6.1 Hz, 2H, H2), 1.25 (d, J = 6.1 Hz, 12H, H1). *Data in agreement with previously published data.*[S3]

Synthesis of compound **3**

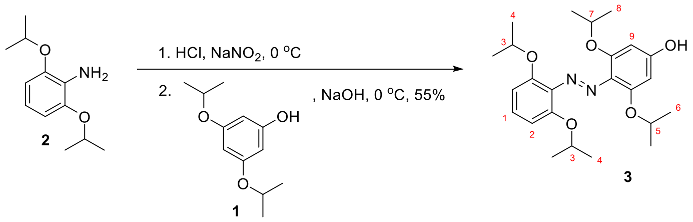

The experimental procedure for compound **3** was inspired by protocols reported by Wang et al.[S3]
Procedure: A solution of 2,6-diisopropoxyaniline (**2**) (700 mg, 3.34 mmol) in water (0.64 mL) and concentrated HCl (0.82 mL) was cooled to 0 - 5 °C in an ice bath. $NaNO_2$ (242 mg, 3.51 mmol) was dissolved in water (3.4 mL) and added dropwise to the cold solution, which immediately turned orange, and the resulting mixture was stirred for 20 minutes in an ice bath. Meanwhile, 3,5-diisopropoxyphenol (**1**) (703 mg, 3.34 mmol) and NaOH (227 mg, 5.69 mmol) were dissolved in water (2.5 mL) resulting in a clear, pale brown solution. The phenolate mixture was cooled to 0-5 °C in an ice bath and then added dropwise to the orange diazonium solution. The reaction was left to stir for 16 hours at room temperature. The formed precipitate was collected, dissolved in ethyl acetate and concentrated *in vacuo*. The crude product was purified by flash column chromatography (diameter: 3 cm, gradient elution from 2%–6% methanol in

ethyl acetate). The product was obtained as an orange solid (793 mg, 1.84 mmol, 55%). **M.P.**: 118.3 – 119.5 °C. **^{1}H NMR** (400 MHz, Chloroform-*d*) δ 11.64 (br s, 1H, OH), 7.03 (t, *J* = 8.4 Hz, 1H, H1), 6.59 (d, *J* = 8.4 Hz, 2H, H2), 5.73 (s, 2H, H9), 4.66-4.53 (m, 4H, H3, H5, H7), 1.48 (d, *J* = 6.1 Hz, 6H, H6), 1.37 (d, *J* = 6.1 Hz, 18H, H4, H8). *Data in agreement with previously published data.*[S3]

Synthesis of compound **12**

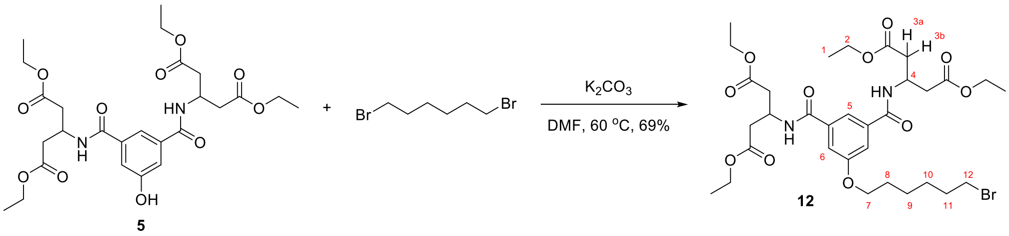

Procedure: Compound **5** (500 mg, 0.905 mmol) and oven dried K_2CO_3 (315 mg, 2.28 mmol) were dissolved in DMF (15 mL). 1,6-Dibromohexane (0.70 mL, 4.6 mmol) was added, and the solution was stirred at 60 °C for 18 hours. The reaction mixture was concentrated *in vacuo* to a final volume of approximately 3 mL. Water (15 mL) was added, and the aqueous phase was extracted with ethyl acetate (1 × 25 mL then 2 × 20 mL). The combined extracts were washed with aqueous NaOH (0.1 M, 15 mL) and brine (15 mL), dried over Na_2SO_4, and concentrated *in vacuo*. The resulting yellow, oily crude product was purified by flash column chromatography (diameter: 3 cm, length: 14 cm, gradient elution from 50%–67% ethyl acetate in heptane) to obtain the product as a white solid (448 mg, 0.626 mmol, 69%). **M.P.**: 67.8 – 68.9 °C. **^{1}H NMR** (400 MHz, Chloroform-*d*) δ 7.67 (t, *J* = 1.3 Hz, 1H, H5), 7.41 (d, *J* = 1.3 Hz, 2H, H6), 7.25 (d, *J* = 8.5 Hz, 2H, NH), 4.81 (dtt, *J* = 8.5, 6.3, 5.6 Hz, 2H, H4), 4.15 (q, *J* = 7.1 Hz, 8H, H2), 4.03 (t, *J* = 6.4 Hz, 2H, H7), 3.42 (t, *J* = 6.8 Hz, 2H, H12), 2.80 (dd, *J* = 16.2, 5.6 Hz, 4H, H3a/H3b), 2.71 (dd, *J* = 16.2, 6.3 Hz, 4H, H3a/H3b), 1.85 (m, 4H, H8, H11), 1.51 (m, 4H, H9, H11), 1.25 (t, *J* = 7.1 Hz, 12H, H1). **^{13}C NMR** (101 MHz, Chloroform-*d*) δ 171.4, 165.8, 159.5, 136.2, 117.3, 116.3, 68.4, 61.0, 43.7, 37.8, 33.9, 32.8, 29.1, 28.0, 25.4, 14.3. **HRMS** (ESI+) *m/z*: [M+H]$^+$ calculated for $C_{32}H_{48}BrN_2O_{11}$ 715.2436; found 715.2448.

Synthesis of compound **13**

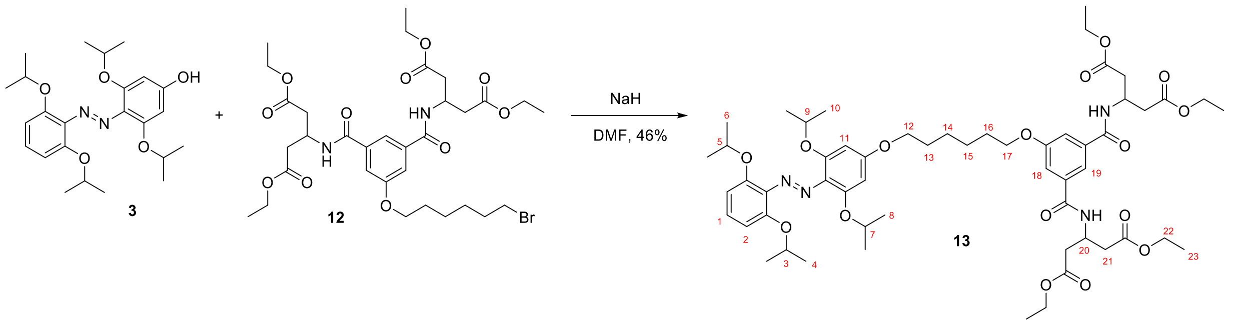

Procedure: Compound **3** (125 mg, 0.290 mmol), compound **12** (204 mg, 0.285 mmol) and NaH (12.2 mg 60% dispersion in mineral oil, 0.305 mmol) were dissolved in dry DMF. The dark red solution was left to stir at room temperature under inert nitrogen atmosphere for 23 hours. The reaction mixture was concentrated *in vacuo* and the residual crude was purified by DCVC (diameter: 2.5 cm, fraction volume: 10 mL, gradient elution from 0%–95% ethyl acetate in heptane in 5% increments). The product eluted in fractions 13-18, which were collected and the solvent was evaporated *in vacuo* to obtain a sticky red solid (140 mg, 0.131 mmol, 46%). **^{1}H NMR**

(400 MHz, DMSO-d_6) δ 8.52 (2 d, J = 8.1 Hz, 4H, NH), 7.80 (d, J = 1.4 Hz, 2H, H19), 7.45 (2 d, J = 1.4 Hz, 4H, H18), 7.13 (t, J = 8.3 Hz, 1H, H1 (trans)), 7.01 (t, J = 8.3 Hz, 1H, H1 (cis)), 6.73 (d, J = 8.3 Hz, 2H, H2 (trans)), 6.50 (d, J = 8.3 Hz, 2H, H2 (cis)), 6.31 (s, 2H, H11 (trans)), 6.07 (s, 2H, H11 (cis)), 4.55 (m, 12H, H3, H5, H7, H9, H20), 4.04 (m, 24H, H12, H17, H22), 2.63 (m, 16H, H21), 1.75 (m, 8H, H13, H16), 1.49 (m, 8H, H14, H15), 1.11 (m, 72H, H4, H6, H8, H10, H23). **^{13}C NMR** (101 MHz, DMSO-d_6) δ 170.5, 165.0, 160.3, 158.4, 152.4, 149.5, 135.9, 130.2, 126.6, 118.7, 115.7, 109.5, 95.9, 71.4, 71.2, 69.7, 69.5, 67.9, 67.7, 60.0, 44.1, 38.6, 28.6, 25.4, 22.0, 21.9, 21.4, 14.0.* **HRMS** (ESI+) *m/z*: $[M+H]^+$ calculated for $C_{56}H_{81}N_4O_{16}$ 1065.5643; found 1065.5675.

*Note: Only signals from the *trans* isomer are listed, as determined from ^{1}H–^{13}C HSQC and ^{1}H–^{13}C HMBC experiments.

<u>Synthesis of template **ipAzo**</u>

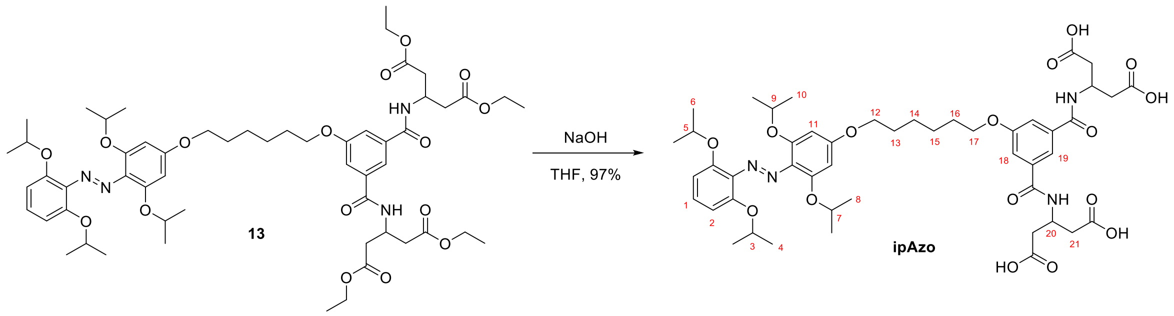

Procedure: Compound **13** (140 mg, 0.131 mmol) was dissolved in THF (5 mL) and aqueous NaOH (1 M, 5 mL) was added. The two-phase solution was stirred at room temperature for 2 hours. The reaction mixture was concentrated *in vacuo* until approximately 2 mL remained, then transferred to a centrifuge tube by use of aqueous NaOH (1 M, ~ 2 mL). The pH was adjusted to 1 by addition of aqueous HCl (6 M, ~ 5 mL), and the formed precipitate was collected and washed with water (2 × 5 mL) upon centrifugation. After drying *in vacuo* the product was obtained as a red solid (121 mg, 0.127 mmol, 97%). **^{1}H NMR** (400 MHz, DMSO-d_6) δ 8.54 (d, J = 8.0 Hz, 2H, NH), 7.88 (t, J = 1.3 Hz, 1H, H19), 7.50 (d, J = 1.3 Hz, 2H, H18), 7.26 (t, J = 8.0 Hz, 1H, H1), 6.80 (d, J = 8.0 Hz, 2H, H2), 6.43 (s, 2H, H11), 4.70 (m, 6H, H3, H5, H7, H9, H20), 4.15 (m, 4H, H12, H17), 2.58 (m, 8H, H21), 1.80 (m, 4H, H13, H16), 1.54 (m, 4H, H14, H15), 1.25 (m, 24H, H4, H6, H8, H10). **^{13}C NMR** (201 MHz, DMSO-d_6) δ 172.2 (**C**OOH), 164.9 (**C**ONH), 158.3 (C17-**O-C**), 150.3 (quaternary),136.0 (C19-**C**-CONH), 128.8 (**C**1), 118.7 (**C**19), 115.7 (**C**18), 108.7 (**C**2), 95.4 (**C**11), 72.1 (**C**H(CH_3), 71.3 (**C**H(CH_3),68.4 (**C**12), 67.9 (**C**17), 44.0 (**C**20), 38.4 (**C**21), 28.6 (**C**H_2), 28.5 (**C**H_2), 25.3 (**C**H_2), 25.2 (**C**H_2), 21.9 (**C**H_3), 21.7 (**C**H_3). *Four quaternary carbons signal from the azobenzene missing due to intermediate exchange on the NMR chemical shift time scale.*

HRMS (ESI+) *m/z*: $[M+H]^+$ calculated for $C_{48}H_{65}N_4O_{16}$ 951.4244; found 951.4263.

S3. UV-vis experiments

All template solutions (50 µM) were prepared in 100 mM sodium phosphate buffered water at pH 7.5 and stored in darkness. In all experiments a reference of air was applied and an absorbance spectrum of the phosphate buffer was subtracted from the sample absorption spectra. At least 1.5 mL of desired solution was added to the cuvette to ensure that the light beam passed though the solution. All UV-vis spectra were measured in the range 220−700 nm with a scan rate of 10 nm/s and a $\Delta\lambda$ of 1 nm. A slit width of 1 nm was applied and lamp change was set at 320 nm.

For photoswitching experiments a general method was applied in all experiments: First, an absorption spectrum was measured before any light treatment. Secondly, the samples were irradiated with light of specific wavelengths until no more change was observed in the absorption spectrum. The cuvette containing the samples were placed at a fixed distance from the light source in a customised box to be shielded from ambient light. The samples were moved directly from the box to the instrument to minimise interference from ambient light, and the measurements were performed instantly.

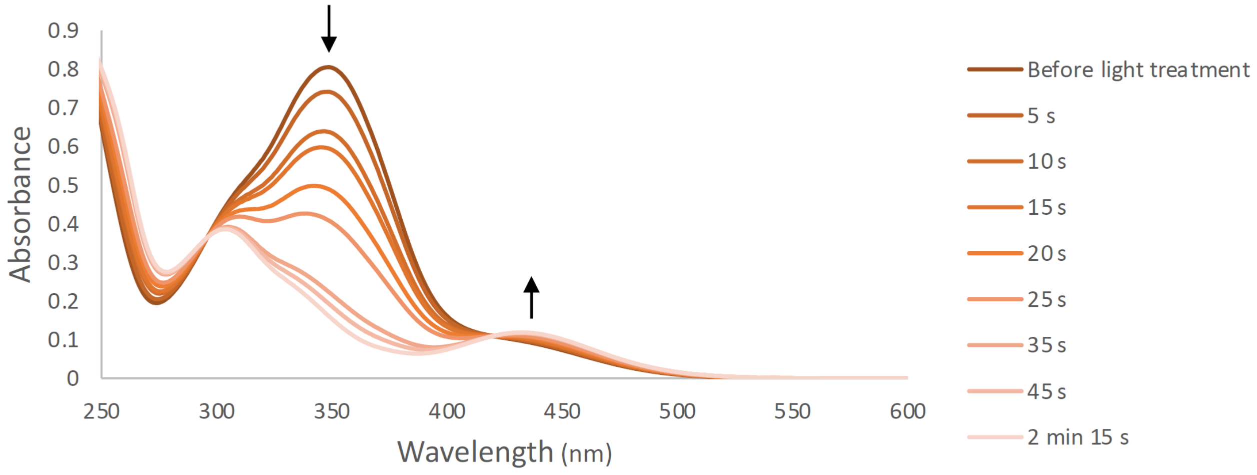

Figure S1: UV-vis absorption spectra of 50 µM **Azo** in 100 mM phosphate buffer, pH 7.5. Before light treatment and after irradiation with light at 365 nm (200 mA) for the times shown.

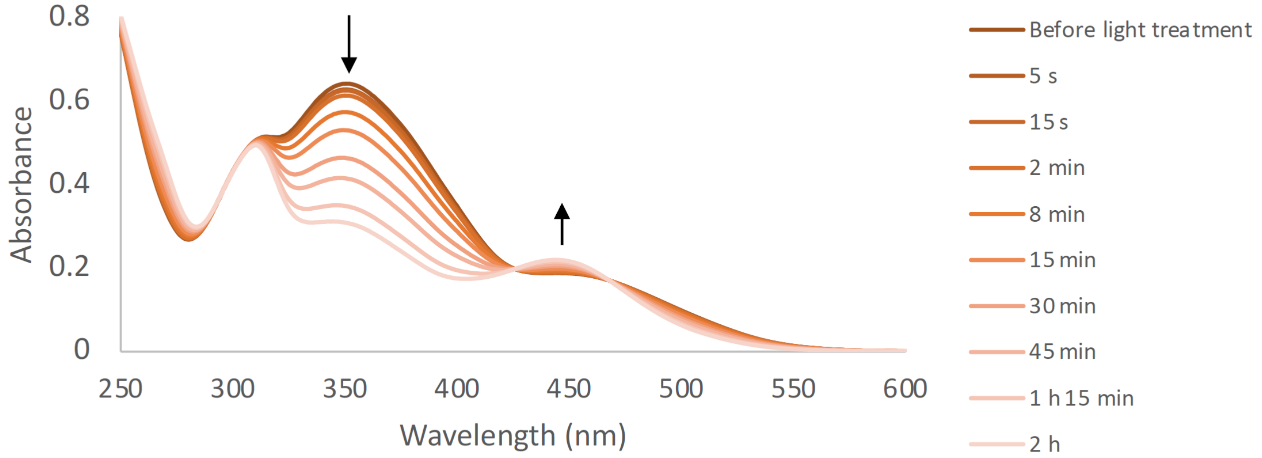

Figure S2: UV-vis absorption spectra of 50 µM **mAzo** in 100 mM phosphate buffer, pH 7.5. Before light treatment and after irradiation with light at 625 nm for the times shown.

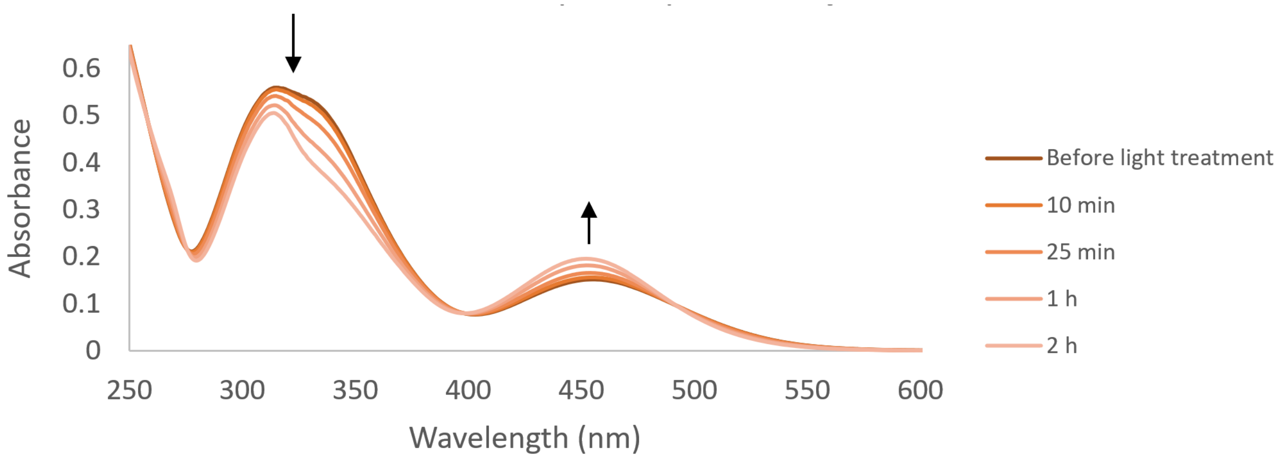

Figure S3: UV-vis absorption spectra of 50 µM **ipAzo** in 100 mM phosphate buffer, pH 7.5. Before light treatment and after irradiation with light at 625 nm for the times shown.

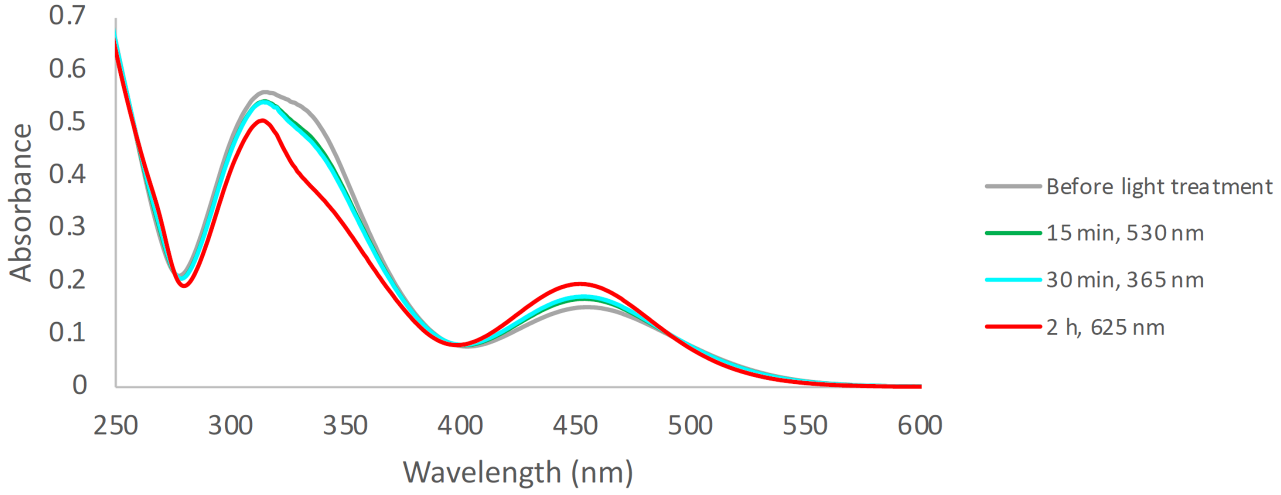

Figure S4: UV-vis absorption spectra of 50 µM **ipAzo** in 100 mM phosphate buffer, pH 7.5. Before light treatment and after irradiation with light at 530 nm, then 365 nm, then 625 nm for the times shown.

S4. NMR experiments

All samples for NMR experiments were prepared in 100 mM phosphate buffered D_2O, pH 7.5. All spectra were acquired at 298 K. Samples were stored in darkness prior to analysis unless otherwise stated. In all irradiation experiments the NMR sample was placed in a customised box with a fixed length from the light source. When handling the NMR samples the tubes were wrapped in foil to avoid irradiation from ambient light. The *cis*/*trans* ratios were determined from integrals of peaks that were assigned exclusively to the *cis*- or *trans*-isomers of the templates.

S4.1 Determination of photostationary states

Photostationary states of the templates were determined at 10 mM for each template. The NMR samples were stored in darkness at 30 °C at least 15 hours prior to analyses. The photostationary states were obtained by irradiating the NMR samples with light of the specific wavelengths until no more change was observed in the obtained NMR spectra.

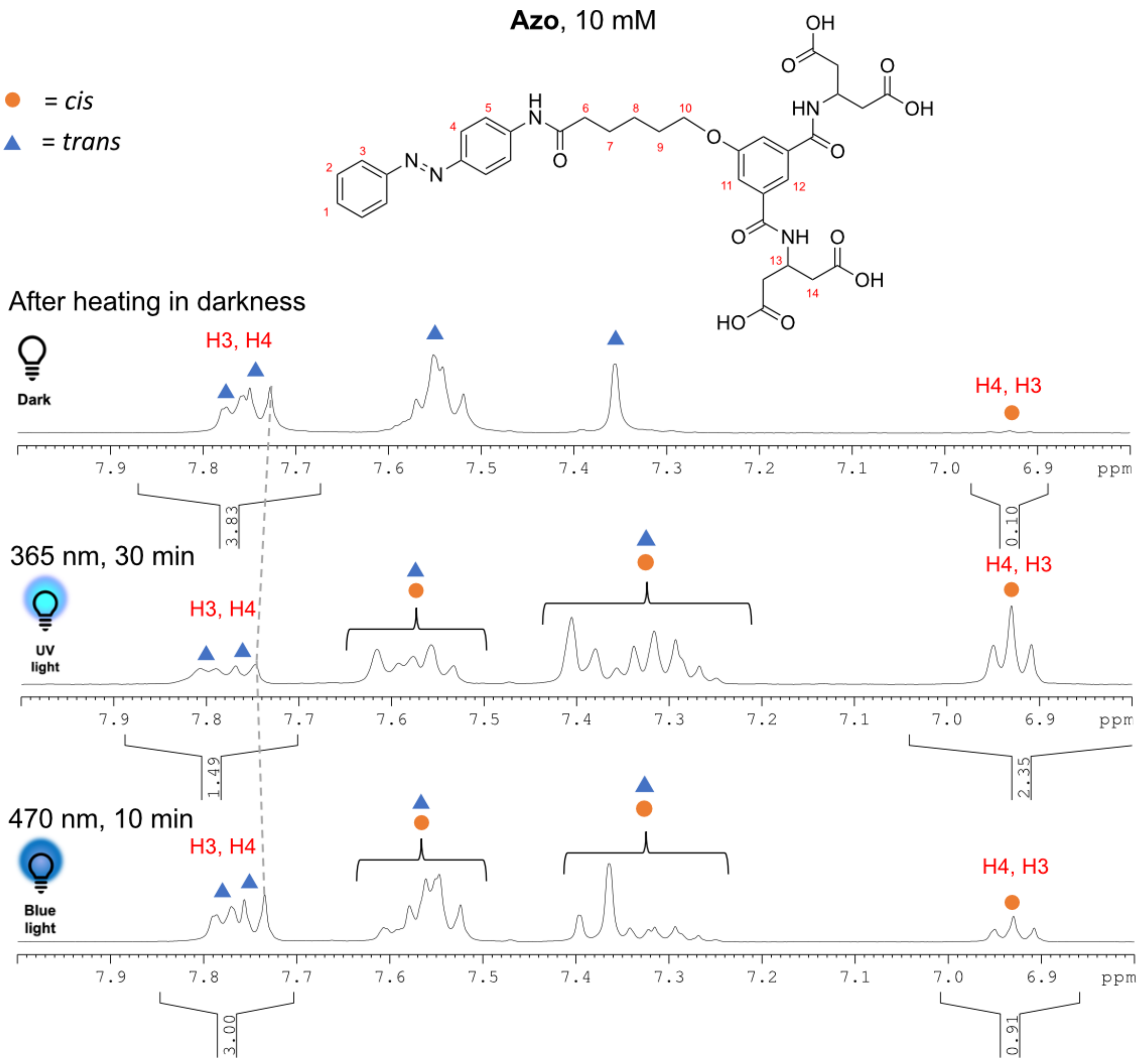

Figure S5: ^{1}H NMR spectra showing the aromatic region for determination of *cis*/*trans* ratios of template **Azo** (10 mM) after heating at 30 °C overnight in the dark, after irradiating with UV light at 365 nm for 30 minutes and after irradiating with blue light for 10 minutes.

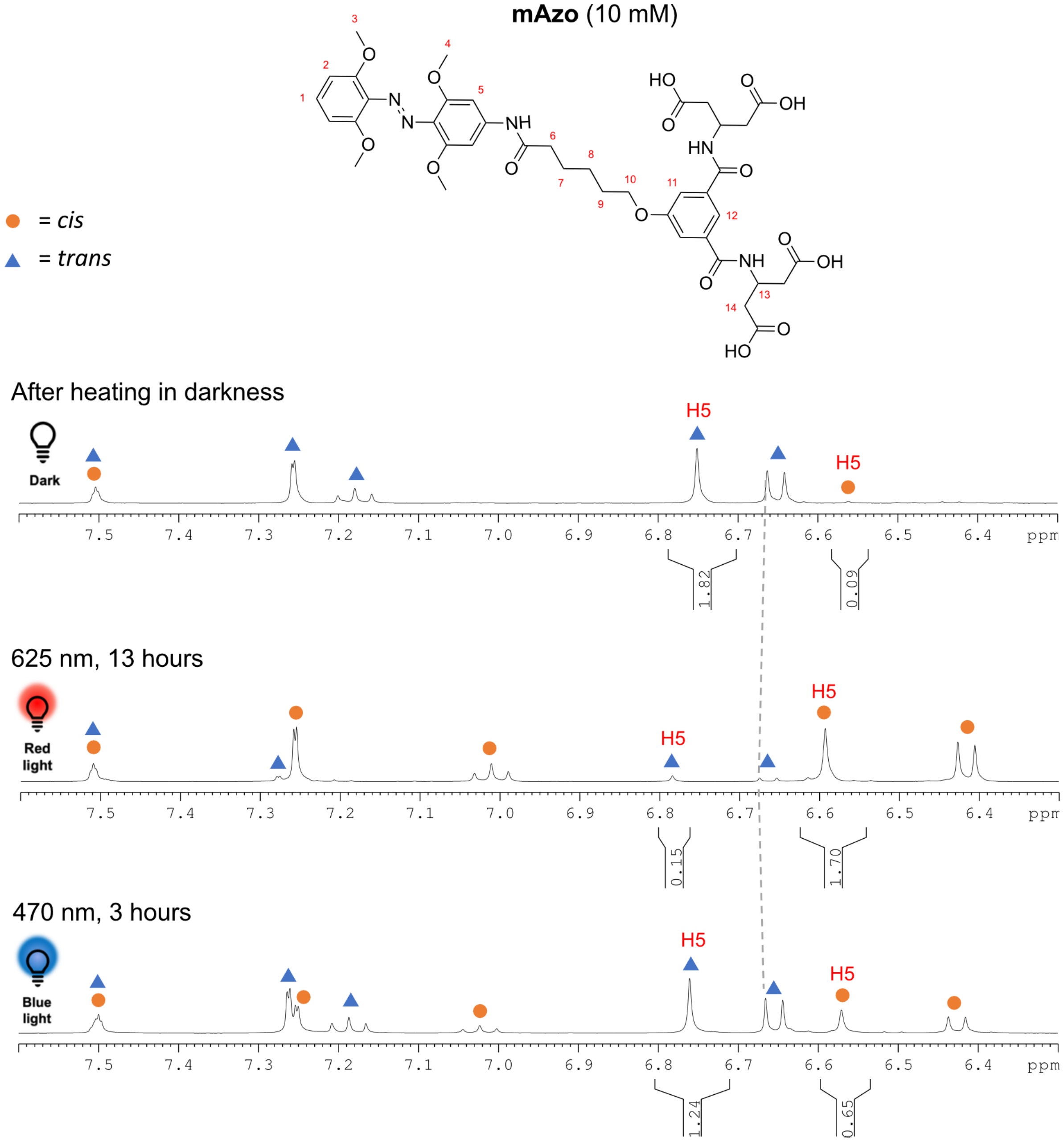

Figure S6: ^{1}H NMR spectra showing the aromatic region for determination of *cis*/*trans* ratios of template **mAzo** (10 mM) after heating at 30 °C overnight in the dark, after irradiating with red light at 625 nm for 13 hours and after irradiating with blue light at 470 nm for 3 hours.

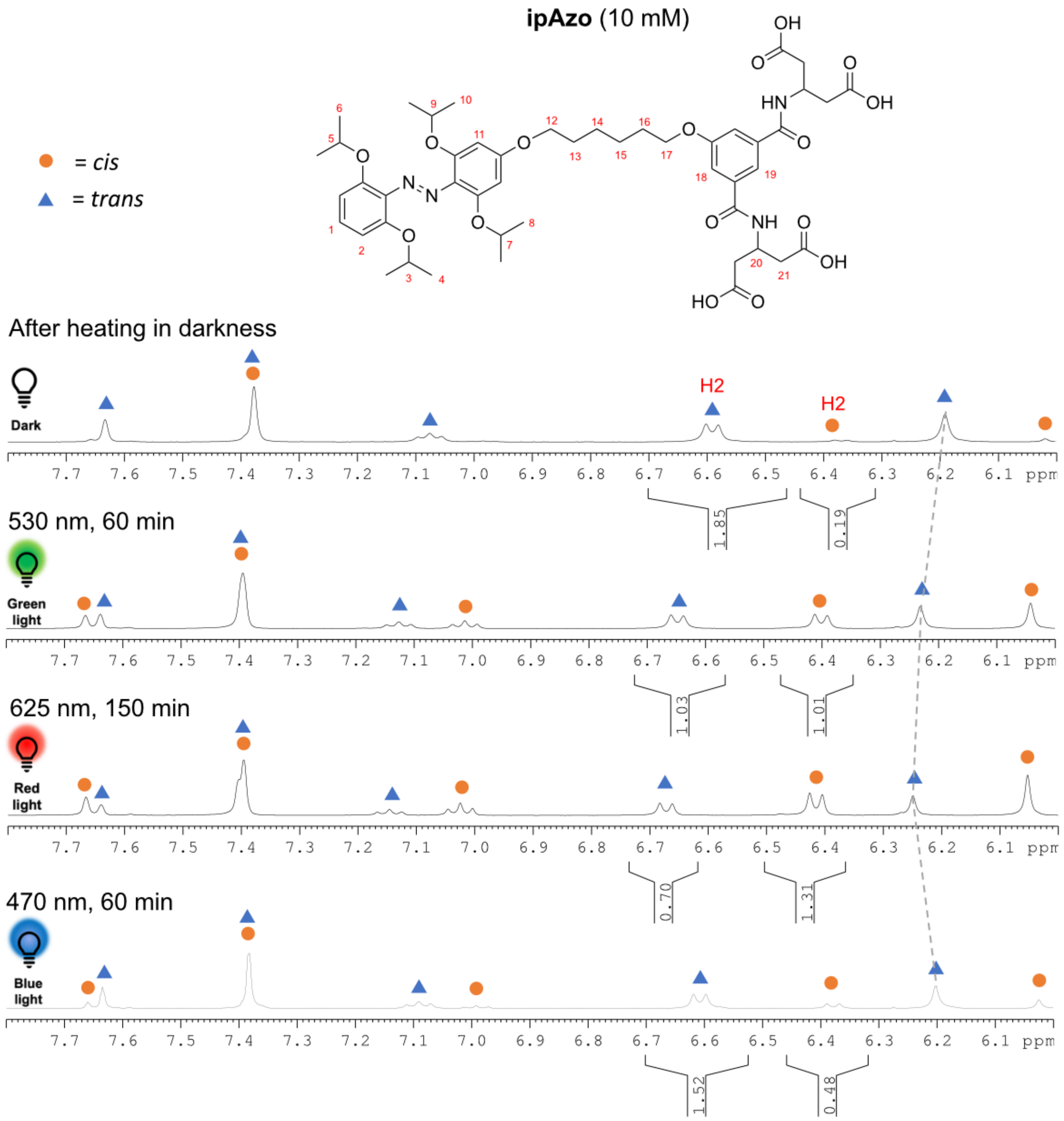

Figure S7: ^{1}H NMR spectra showing the aromatic region for determination of *cis*/*trans* ratios of template **ipAzo** (10 mM) after heating at 30 °C overnight in the dark, after irradiating with green light at 530 nm for 60 minutes, after irradiating with red light at 625 nm for 150 minutes and after irradiating with blue light at 470 nm for 60 minutes.

S4.2 Aggregation studies

To investigate the aggregation of the templates, a dilution series of the templates were analysed using ^{1}H NMR spectroscopy. The samples were investigated before light treatment, since it would be expected that the *trans*-isomers would have the strongest tendency to aggregate. 10 mM stock solutions of each template (**Azo**, **mAzo**, or **ipAzo**) in 100 mM phosphate buffered D_2O, pH 7.5 were prepared and analysed by ^{1}H NMR spectroscopy. By diluting with 100 mM phosphate buffered D_2O, pH 7.5, a dilution series down to 0.05 mM was acquired for each template.

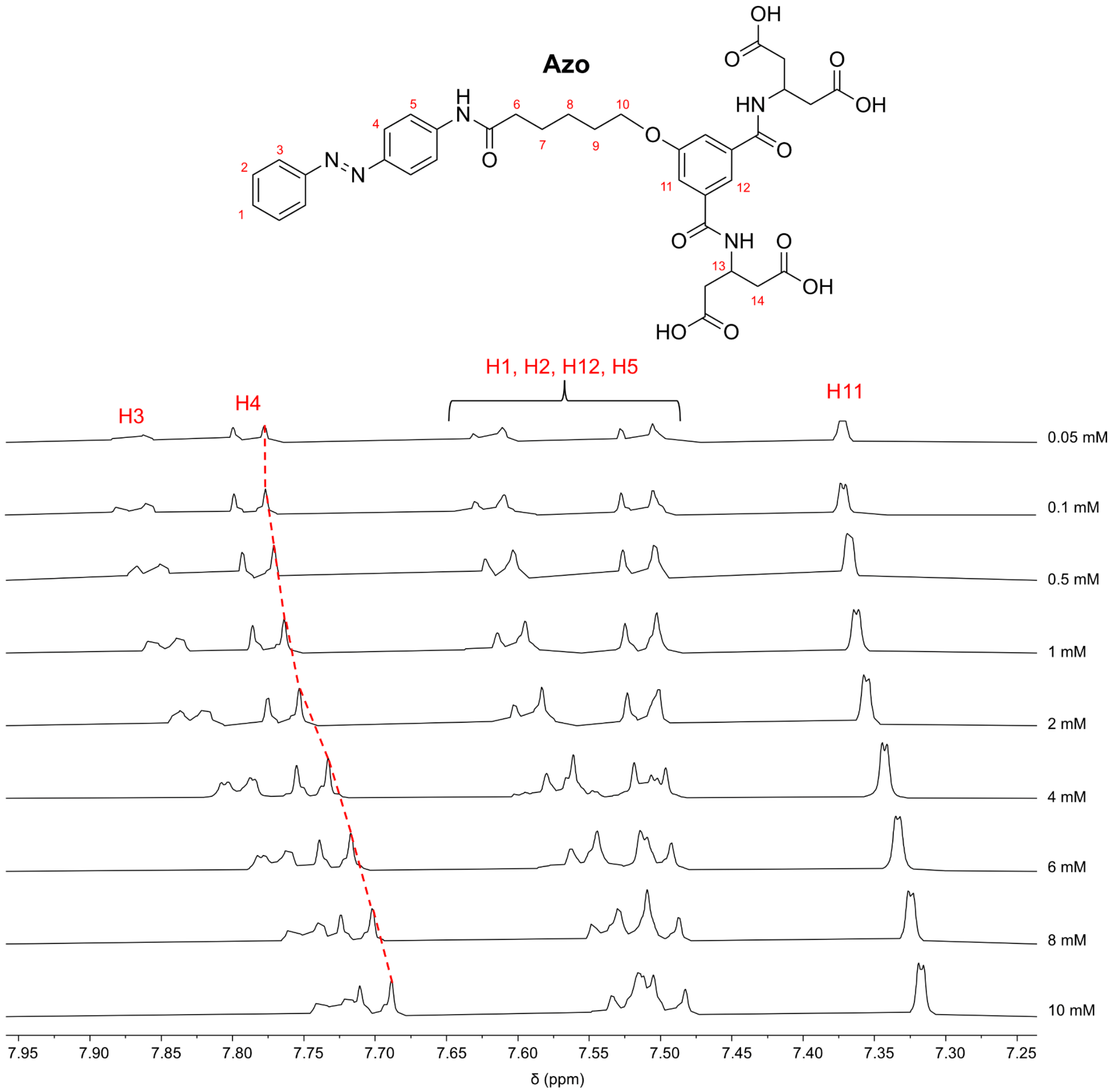

Figure S8: ^{1}H NMR spectra showing the aromatic region for template **Azo** at different concentrations. The dashed red lines indicate how the marked peak moves upon concentration changes.

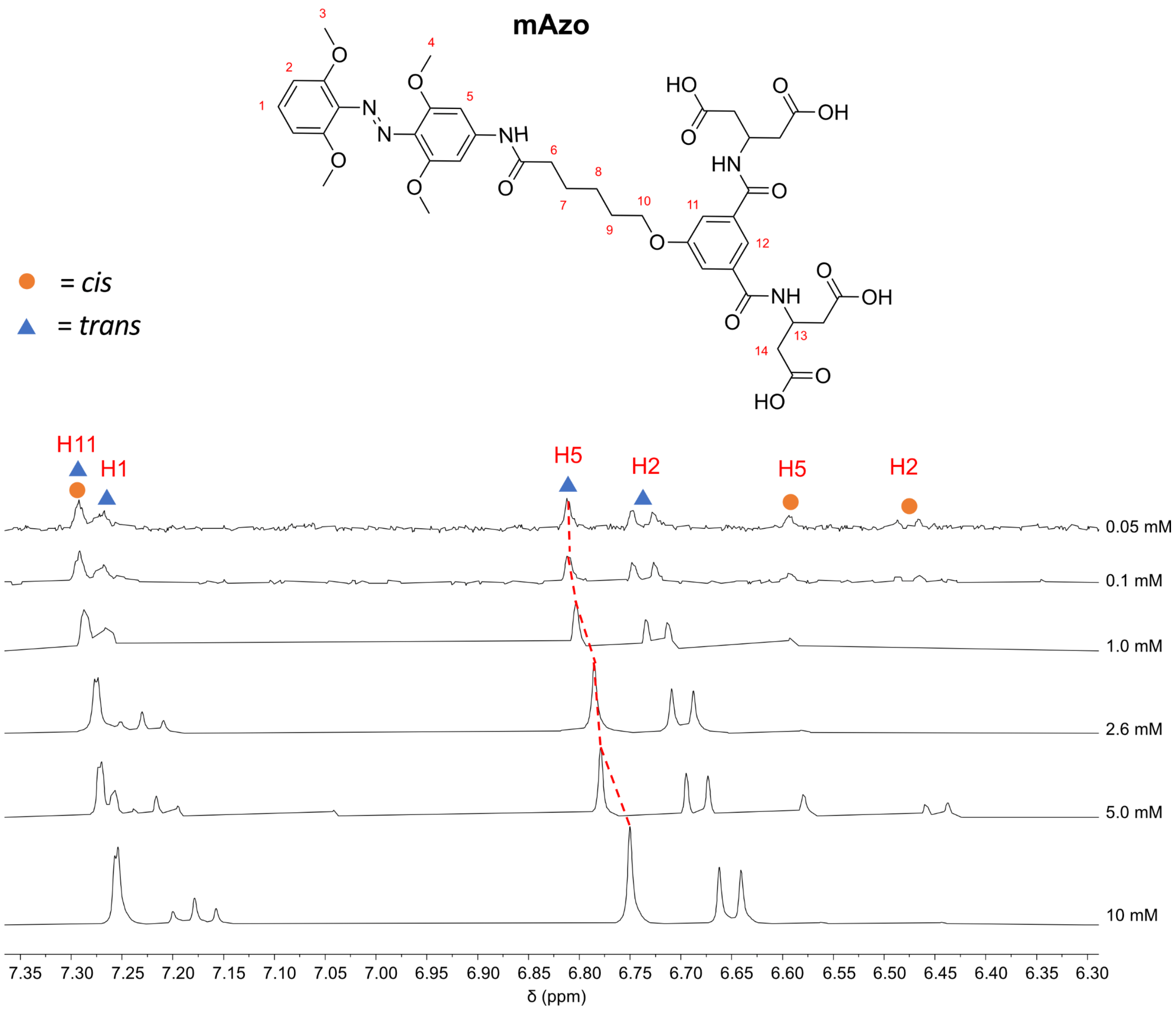

Figure S9: ^{1}H NMR spectra showing the aromatic region for template **mAzo** at different concentrations. The dashed red lines indicate how the marked peak moves upon concentration changes. The changes in *cis/trans* distributions are due to contamination with ambient light.

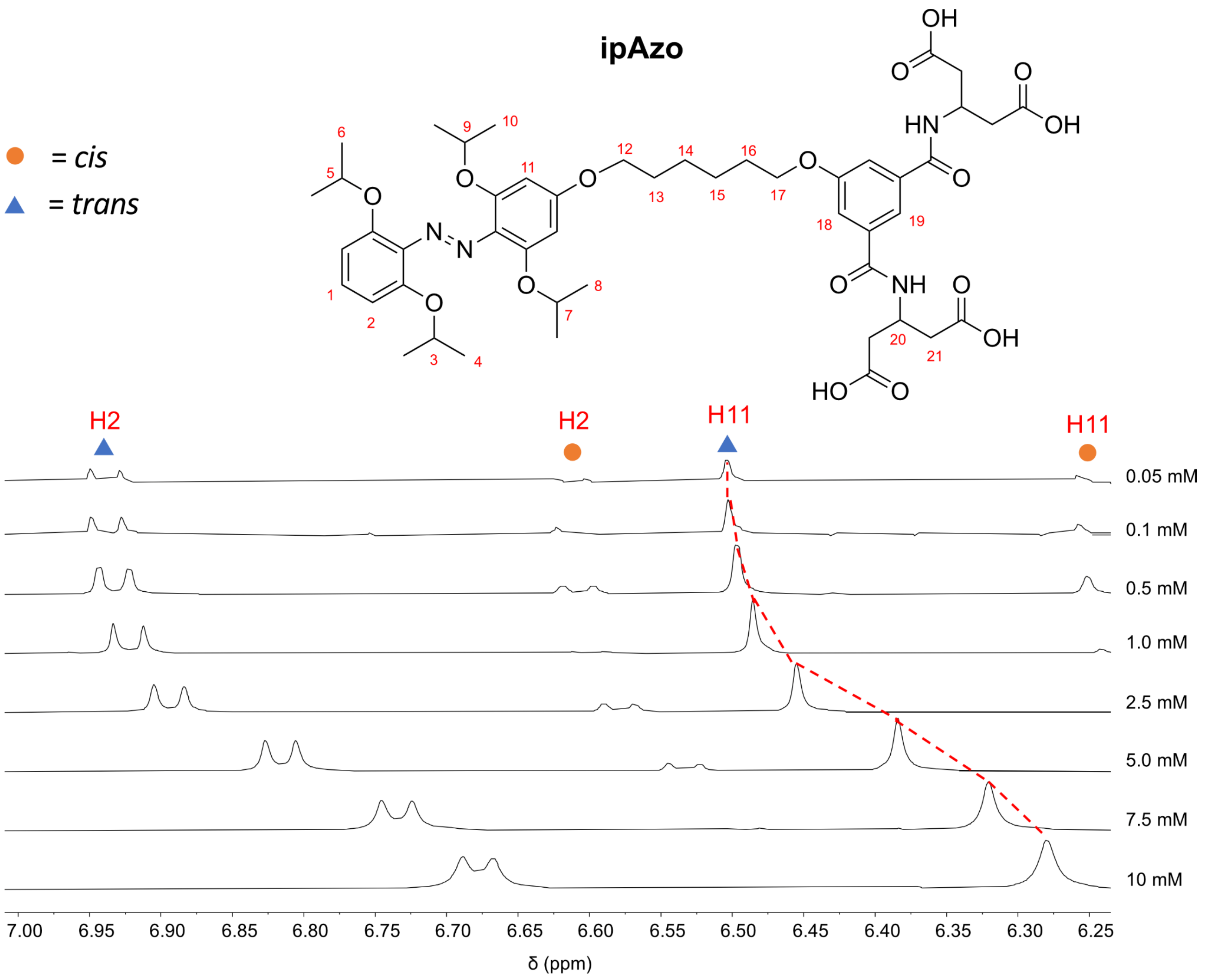

Figure S10: ^{1}H NMR spectra showing the aromatic region for template **ipAzo** at different concentrations. The dashed red lines indicate how the marked peak moves upon concentration changes.

S4.3 ^{1}H NMR titrations

For each titration a ‘host’ solution and a ‘guest’ solution were prepared. A 0.1 mM host solution was prepared by dissolving the desired template **Azo**, **mAzo**, or **ipAzo** in 100 mM phosphate buffered D_2O, pH 7.5. The guest solution was prepared by dissolving the desired amount of guest (α-, β-, or γ-CD) in the 0.1 mM host solution. 500 μL host solution was transferred to an NMR tube. For the titrations of the *trans*-isomers, the NMR sample and guest solution were stored in darkness at 30 °C for at least 15 hours prior to a titration experiment. For the titrations to investigate binding by the *cis*-isomer, the NMR sample was irradiated with light at appropriate wavelengths prior to the titration experiment and for five minutes after each addition of guest solution prior to acquisition. ^{1}H NMR spectra were acquired at increasing concentrations of cyclodextrin guest.

For titration experiments with one host the change in chemical shift of selected proton signals were plotted against the corresponding guest concentrations and the binding isotherm was fitted non-linearly to a 1:1 binding model, as described in the equation below.[S7]

$$\Delta\delta = \frac{\Delta\delta_{max}}{2[H]_0}\cdot\frac{1}{2}\left(\left(\frac{1}{K_a}+[H]_0+[G]_0\right)-\sqrt{\left(\frac{1}{K_a}+[H]_0+[G]_0\right)^2-4\cdot[H]_0\cdot[G]_0}\right)$$

For relevant titration experiments with both the *trans*- and *cis*-hosts in solution the chemical shift changes of a *cis*-proton was plotted against the chemical shift changes of a *trans*-proton. When a binding constant, K_a, and the maximum chemical shift change, $\Delta\delta_{a,max}$, for a *trans*-host-guest complex are known (from the titration performed in the dark), the binding constants for the corresponding *cis*-host-guest complex can be determined by fitting the data to the equation below.[S8]

$$\Delta\delta_b = \frac{\Delta\delta_{b,max}\cdot\Delta\delta_a\cdot K_b}{\Delta\delta_{a,max}\cdot K_a - \Delta\delta_a\cdot K_a + \Delta\delta_a\cdot K_b}$$

When applying this method uncertainties determined for the K_b and $\Delta\delta_{b,max}$ values for the *cis*-isomer were compounded with the fitting errors on the values determined for the *trans*-isomer, since the latter values were used to calculate the former.

For all fitting procedures the data analysis software OriginPro (Version 2021. OriginLab Corporation, Northampton, MA, USA) was applied.

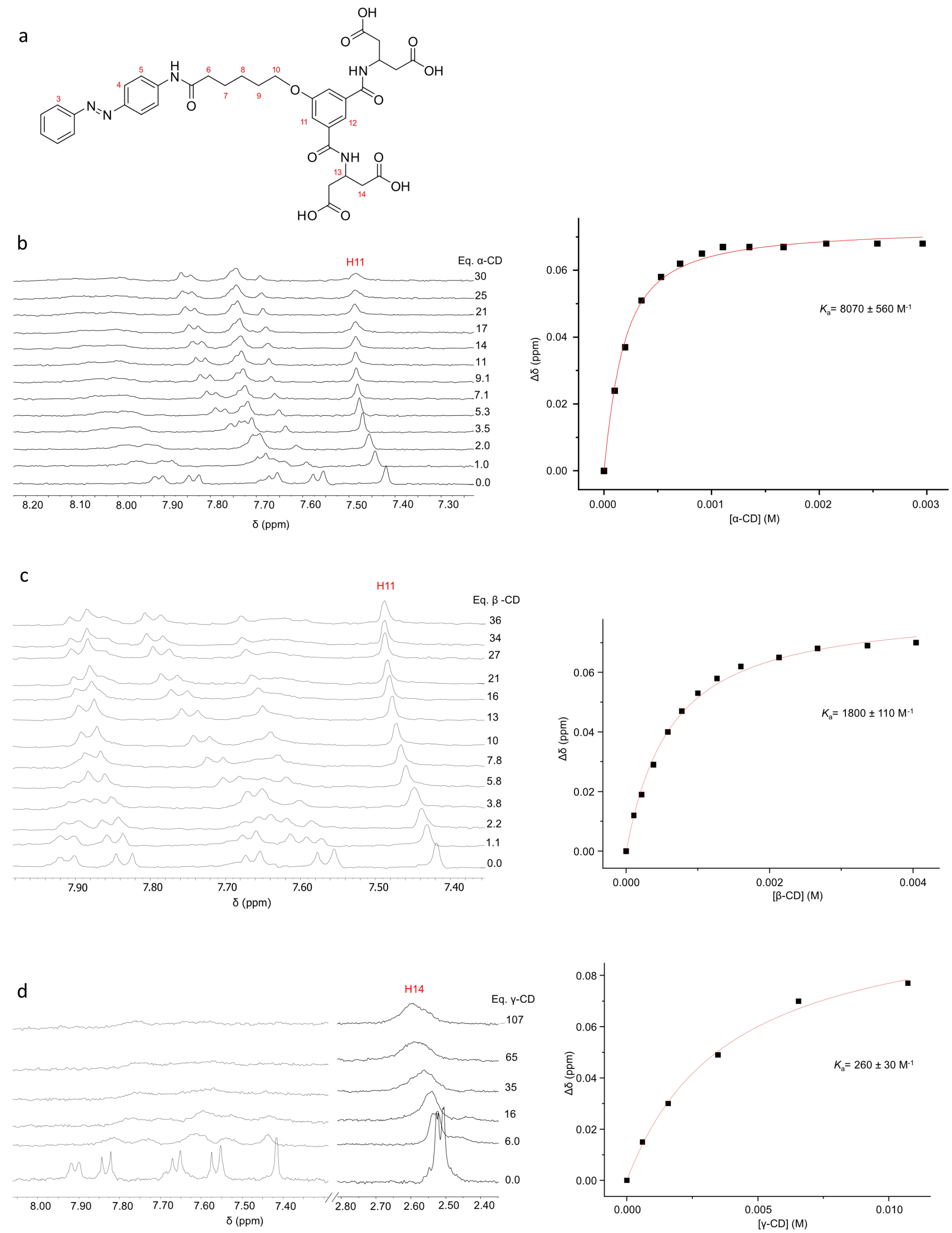

Figure S11: ^{1}H NMR titration of *trans*-**Azo** with α-CD, β-CD and γ-CD. a) *trans*-**Azo** with assigned proton numbers. Partial ^{1}H NMR spectra and fitted 1:1 binding isotherms based on $\Delta\delta_{obs}$ of the indicated protons upon addition of b) α-CD, c) β-CD, and d) γ-CD. Conditions: the titrations were performed with 0.1 mM template solutions in 100 mM phosphate buffer in D_2O, pH 7.5, at 298 K.

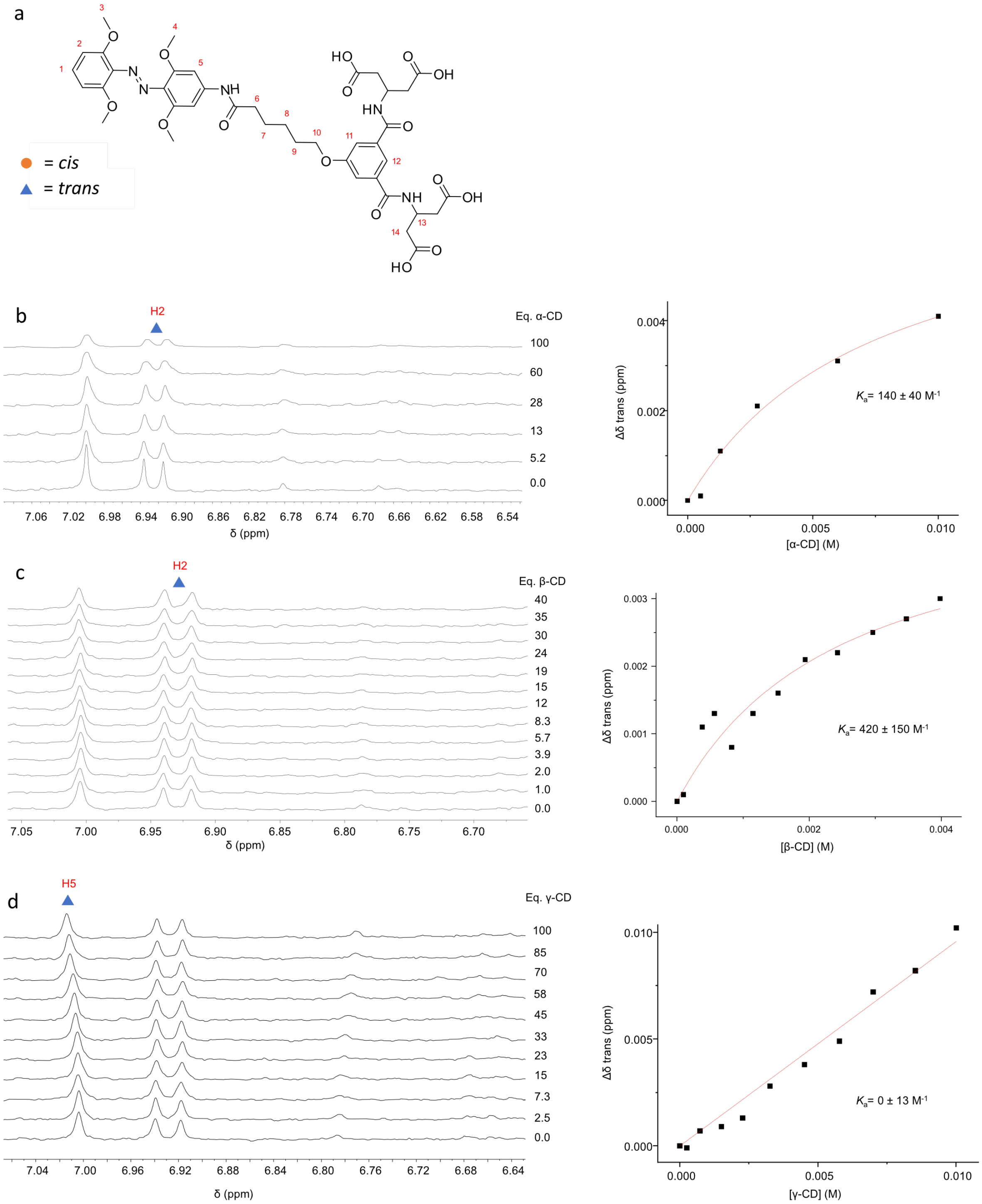

Figure S12: ^{1}H NMR titration of *trans*-**mAzo** with α-CD, β-CD and γ-CD. a) *trans*-**mAzo** template with assigned proton numbers. Partial ^{1}H NMR spectra of *trans*-**mAzo** and fitted 1:1 binding isotherms based on observed chemical shift change for the marked protons upon addition of b) α-CD, c) β-CD, and d) γ-CD. Conditions as in Figure S11.

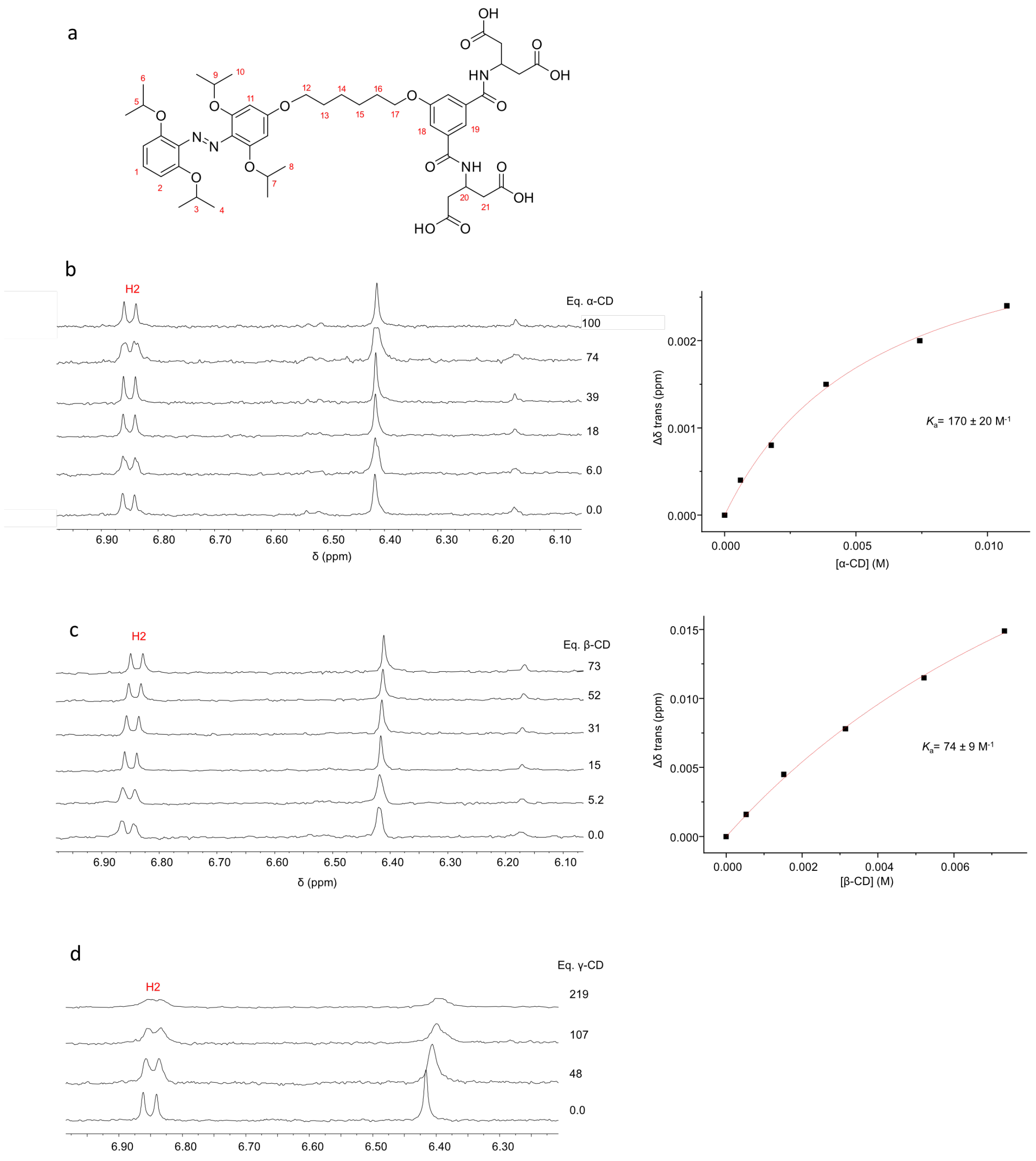

Figure S13: ^{1}H NMR titration of *trans*-**ipAzo** with α-CD, β-CD and γ-CD. a) *trans*-**ipAzo** template with assigned proton numbers. Partial ^{1}H NMR spectra of *trans*-**ipAzo** and where relevant fitted 1:1 binding isotherms based on observed chemical shift change for proton H2 upon addition of b) α-CD, c) β-CD, and d) γ-CD. The binding affinity between *tras*-**ipAzo** and γ-CD was too weak to quantify. Conditions as in Figure S11.

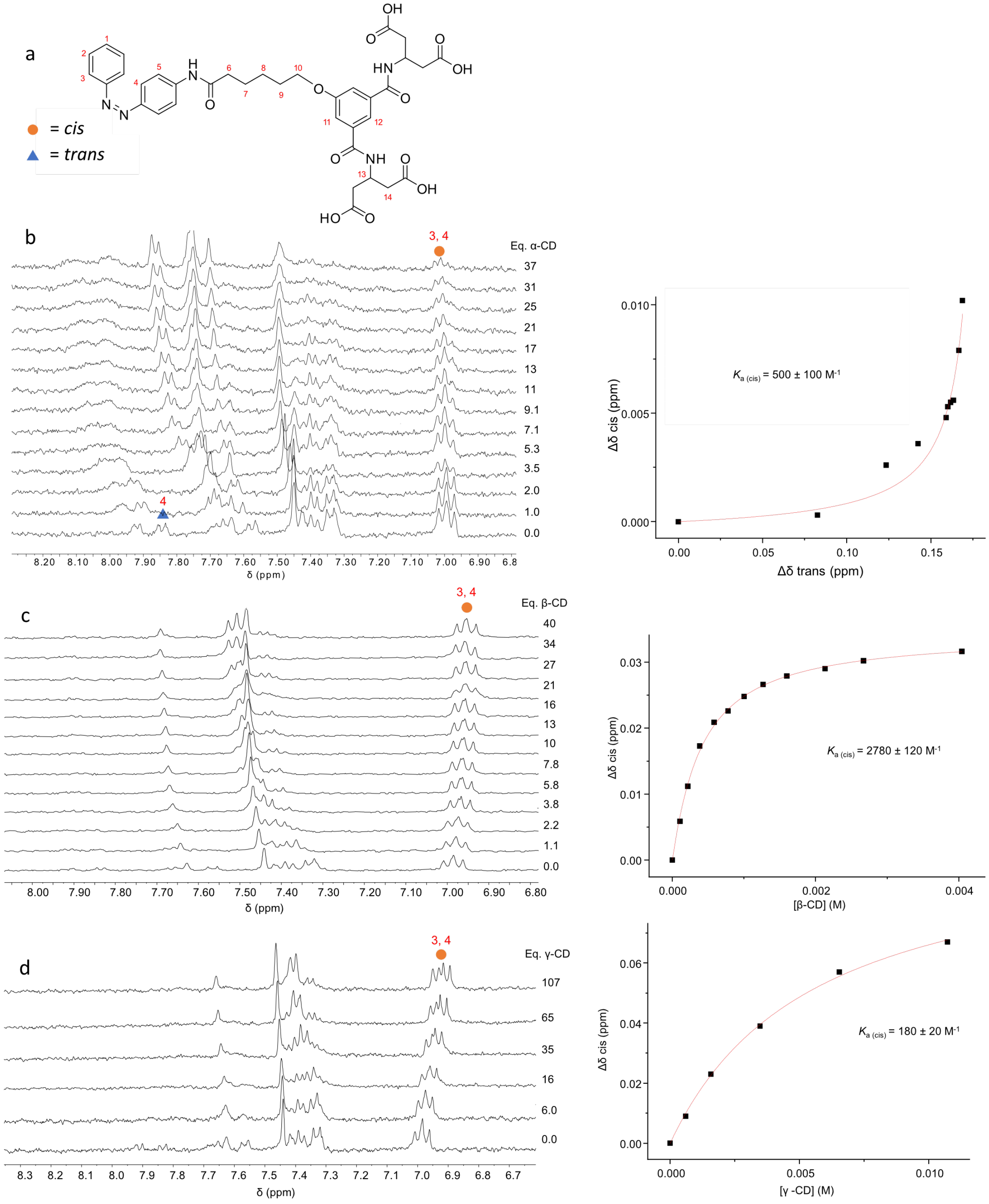

Figure S14: ^{1}H NMR titrations to determine binding constants for *cis*-**Azo** with α-CD, β-CD and γ-CD. a) *cis*-**Azo** template with assigned proton numbers. b) Partial ^{1}H NMR spectra of a mixture of *cis* and *trans* **Azo** and fitted plot of $\Delta\delta_{cis}$ versus $\Delta\delta_{trans}$ based on observed chemical shift changes of the marked signals upon addition of α-CD. Partial ^{1}H NMR spectra of *cis*-**Azo** and fitted 1:1 binding isotherms based on $\Delta\delta_{obs}$ for the right side of the marked signal corresponding to the *cis*-protons H3 and H4 upon addition of c) β-CD and d) γ-CD. Conditions as in Figure S11.

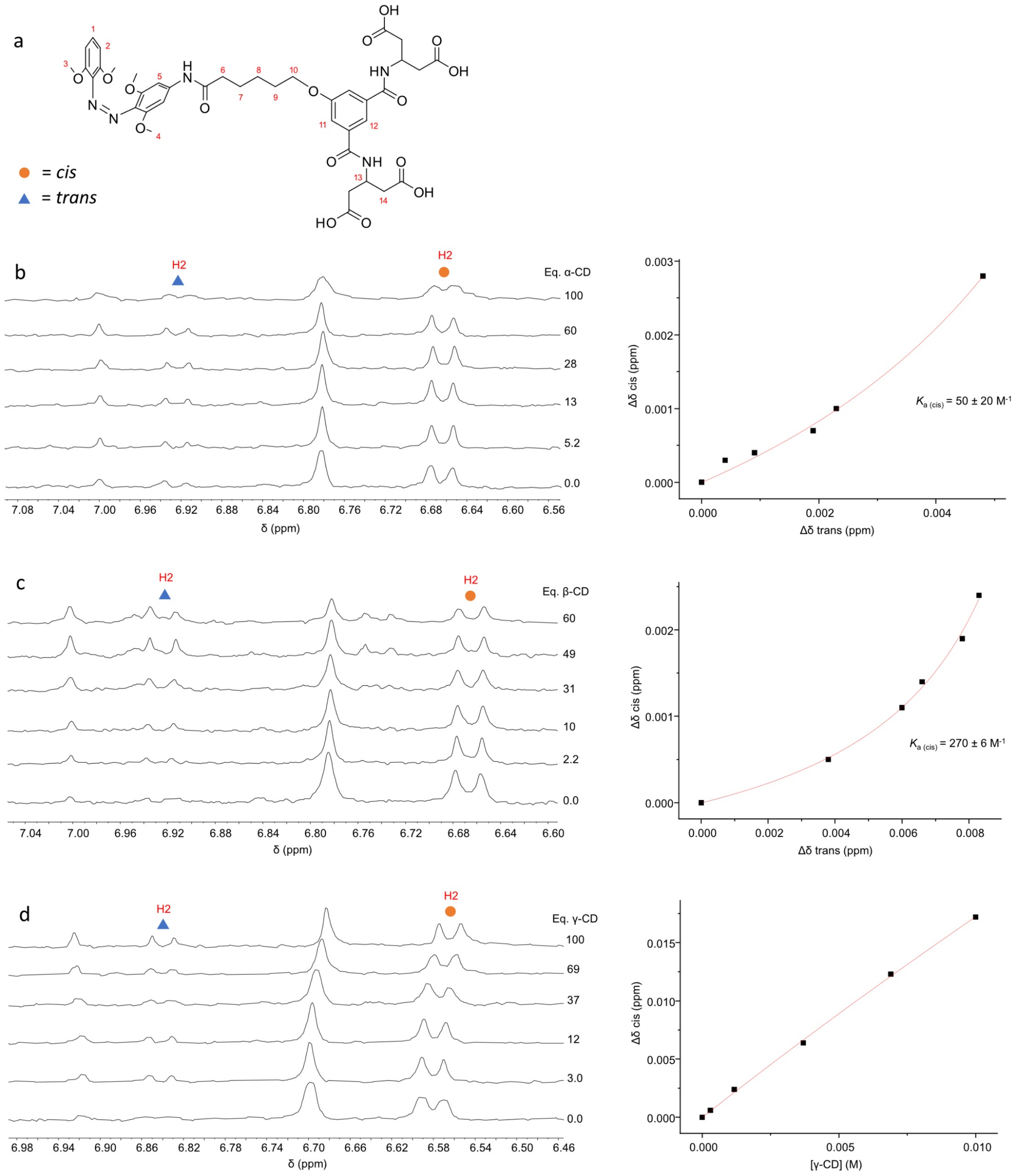

Figure S15: ^{1}H NMR titrations to determine binding constants for *cis*-**mAzo** with α-CD, β-CD and γ-CD. a) *cis*-**mAzo** template with assigned proton numbers. Partial ^{1}H NMR spectra of a mixture of *cis*- and *trans*- **mAzo** and fitted plots of $\Delta\delta_{cis}$ versus $\Delta\delta_{trans}$ based on observed chemical shift changes for the marked signals upon addition of b) α-CD and c) β-CD. d) Partial ^{1}H NMR spectra of a mixture of *cis*- and *trans*- **mAzo** upon addition of γ-CD. Binding was too weak to quantify. Conditions as in Figure S11.

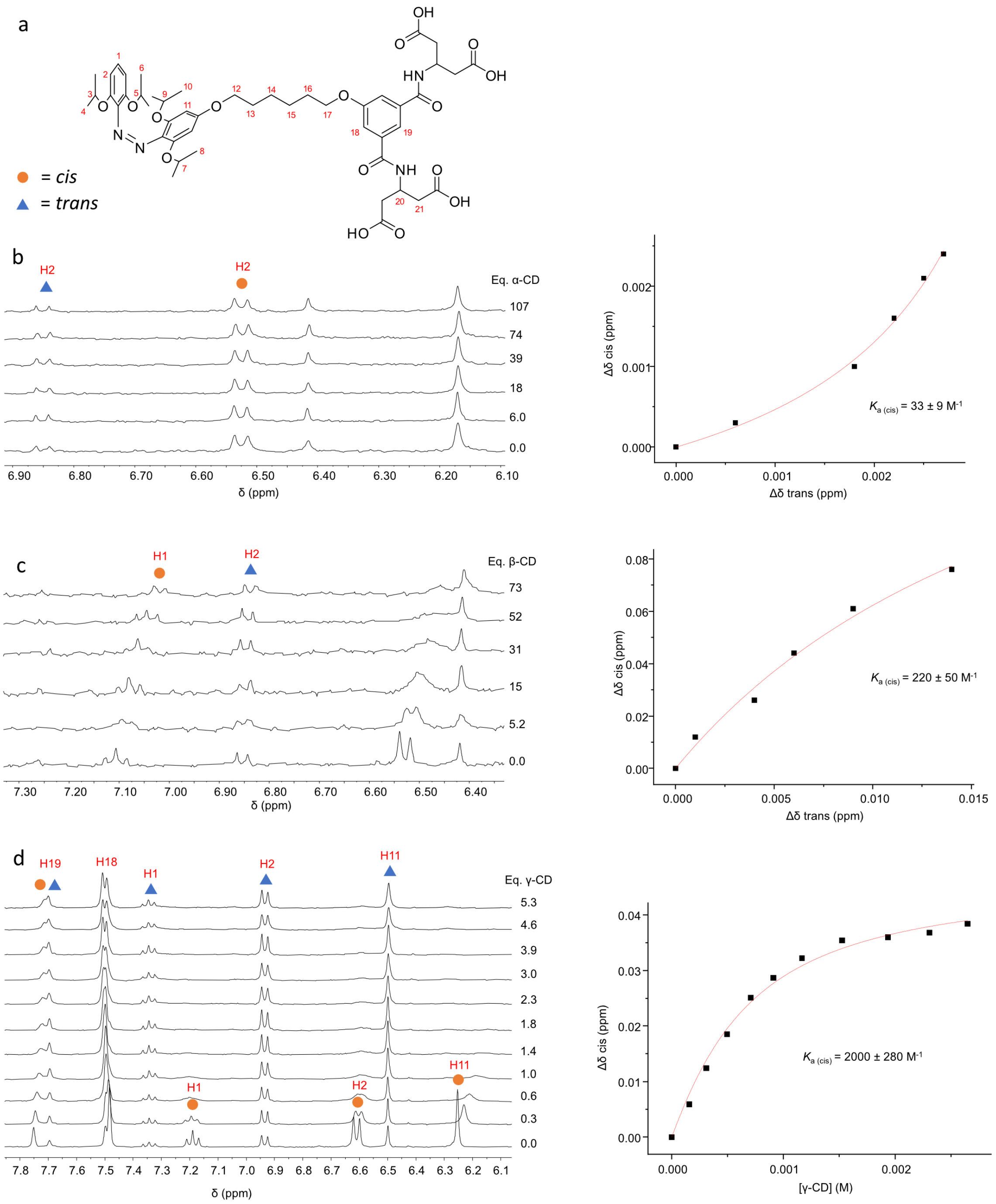

Figure S16: ^{1}H NMR titrations to determine binding constants for *cis*-**ipAzo** with α-CD, β-CD and γ-CD. a) *cis*-**ipAzo** template with assigned proton numbers. Partial ^{1}H NMR spectra of a mixture of *cis*- and *trans*-**ipAzo** and fitted plot of $\Delta\delta_{cis}$ versus $\Delta\delta_{trans}$ based on observed chemical shift changes of the marked signals upon addition of b) α-CD and c) β-CD. d) Partial ^{1}H NMR spectra of a mixture of *cis*- and *trans*- **ipAzo** and fitted 1:1 binding isotherms based on the observed chemical shift change for H19 upon addition of γ-CD. The binding interaction between γ-CD with *trans*-**ipAzo** was judged to be so weak that competitive binding could be disregarded when determining the association constant for the interaction between γ-CD and *cis*-**ipAzo**. Conditions as in Figure S11, except the titration with γ-CD which was performed with 0.5 mM template solution.

Table S1: Fitting parameter for template **Azo** determined for the association constants, K_a (M^{-1}), and the maximum change in chemical shift, $\Delta\delta_{max}$ (ppm) and the corresponding fitting errors.

Azo	Isomer	Fitting parameter	Value from fit	Error	Comp. error
**α-CD**	*trans*	$K_{a\ (trans)}$	8072.22	563.236	-
		$\Delta\delta_{max\ (trans)}$	0.07292	0.00079	-
	cis	$K_{a\ (cis)}$	534.011	105.475	142.532
		$\Delta\delta_{max\ (cis)}$	0.00959	0.00058	-
**β-CD**	*trans*	$K_{a\ (trans)}$	1802.18	111.340	-
		$\Delta\delta_{max\ (trans)}$	0.08212	0.00146	-
	cis	$K_{a\ (cis)}$*	2778.54	122.921	-
		$\Delta\delta_{max\ (cis)}$*	0.03445	0.00038	-
**γ-CD**	*trans*	$K_{a\ (trans)}$	261.155	29.2876	-
		$\Delta\delta_{max\ (trans)}$	0.10667	0.00048	-
	cis	$K_{a\ (cis)}$*	183.408	13.5740	-
		$\Delta\delta_{max\ (cis)}$*	0.10236	0.00351	-

*The fitting values were determined from the best fit to a 1:1 binding isotherm.

Table S2: Fitting parameter for template **mAzo** determined for the association constants, K_a (M^{-1}), and the maximum change in chemical shift, $\Delta\delta_{max}$ (ppm) and the corresponding fitting errors.

mAzo	Isomer	Fitting parameter	Value from fit	Error	Comp. error
**α-CD**	*trans*	$K_{a\ (trans)}$	135.634	40.3628	-
		$\Delta\delta_{max\ (trans)}$	0.00712	0.00112	-
	cis	$K_{a\ (cis)}$	54.0374	13.6208	29.0601
		$\Delta\delta_{max\ (cis)}$	0.00619	0.00096	0.00193
**β-CD**	*trans*	$K_{a\ (trans)}$	417.938	152.469	-
		$\Delta\delta_{max\ (trans)}$	0.00460	0.00084	-
	cis	$K_{a\ (cis)}$	265.494	6.12795	102.98
		$\Delta\delta_{max\ (cis)}$	0.000696	0.000030	0.00016
**γ-CD**	*trans*	$K_{a\ (trans)}$	*Binding affinity not strong enough to quantify*		
		$\Delta\delta_{max\ (trans)}$			
	cis	$K_{a\ (cis)}$*	7.0795	4.6150	-
		$\Delta\delta_{max\ (cis)}$*	0.26116	0.16058	-

*The fitting values were determined from the best fit to a 1:1 binding isotherm.

Table S3: Fitting parameter for template **ipAzo** determined for the association constants, K_a (M^{-1}), and the maximum change in chemical shift, $\Delta\delta_{max}$ (ppm) and the corresponding fitting errors.

ipAzo	Isomer	Fitting parameter	Value from fit	Error	Comp. error
α-CD	*trans*	$K_{a\ (trans)}$	173.900	21.1656	-
		$\Delta\delta_{max\ (trans)}$	0.00365	0.00021	-
	cis	$K_{a\ (cis)}$	33.4633	9.12077	13.1936
		$\Delta\delta_{max\ (cis)}$	0.00691	0.00133	0.00173
β-CD	*trans*	$K_{a\ (trans)}$	74.1428	9.13784	-
		$\Delta\delta_{max\ (trans)}$	0.004208	0.000367	-
	cis	$K_{a\ (cis)}$	221.674	54.0142	77.1142
		$\Delta\delta_{max\ (cis)}$	0.12919	0.01664	0.02791
γ-CD	*trans*	$K_{a\ (trans)}$	*Binding affinity not strong enough to quantify*		
		$\Delta\delta_{max\ (trans)}$			
	cis	$K_{a\ (cis)}$*	2016.49	280.823	-
		$\Delta\delta_{max\ (cis)}$*	0.04721	0.00190	-

*The fitting values were determined from the best fit to a 1:1 binding isotherm.

S5. CGTase-mediated dynamic combinatorial libraries of cyclodextrins

All reactions were set up in clear 2 mL glass vials. For libraries under irradiation with light, the vials were placed in a fitted chamber in the exact same distance from the light source to ensure consistency. The libraries were irradiated with light at all times during the studies, except when taking out samples for analysis. The libraries were irradiated before starting the library for a period of time that ensured full conversion to photostationary state (30 mins at 365 nm for **Azo**, overnight at 625 nm for **mAzo** and 90 mins at 625 nm for **ipAzo**). The libraries performed in darkness were stored in darkness a minimum of 15 hours before starting the library and were kept in darkness at all time during the studies, except when taking out samples for analysis. The spin-filtered CGTase enzyme stock solution was used with addition of 65 µL enzyme mixture per mL library reaction mixture in all cases to ensure the desired activity. All libraries were performed at room temperature.

The reference libraries (untemplated) were prepared from a solution of 10 mg/mL α-CD in 100 mM sodium phosphate buffer at pH 7.5. Libraries were started by addition of CGTase stock solution. The templated libraries were prepared from a solution of 10 mg/mL α-CD with the desired template(s) at 10 mM in 100 mM sodium phosphate buffer at pH 7.5. Libraries were started by addition of CGTase stock solution.

S5.1 Sampling and analysis method

The libraries were analysed over time by taking out 3 µL samples from the library which were added to a 120 µL quenching mixture consisting of 10 mM ammonium chloride in acetonitile/water (3:1) with 1% TFA. The 123 µL mixture was centrifuged at 10000 rpm for 4 minutes in a Heraeus Biofuge Pico. After centrifugation, the top 110 µL fraction were transferred to an HPLC insert in a HPLC vial capped with screw caps equipped with PTFE lined rubber septa for injection in the HPLC-ELSD system. An injection volume of 10 µL was used in all cases. The eluent system was water (A) and acetonitrile (B), both with 0.1% formic acid. For separation, a linear gradient program running from 25 to 45% eluent A over 8.00 minutes with a flow rate of 0.60 mL/min was used, after which the column was washed for three minutes with 100% eluent A. Before each injection, at least five column volumes of the starting eluent were applied to condition the column. The resulting chromatograms were analysed and the peak area was converted to a concentration (by weight) through calculations based on calibrations performed individually for the short linear α-1,4-glucans glucose (G1), maltose (G2), and maltotriose (G3) as well as α-CD, β-CD, and γ-CD by analysing stock solutions using the above-mentioned chromatographic method. The concentrations of the longer linear α-1,4-glucans (G4−G8) were calculated based on the G3 calibration. The calibrations were based on µg injected in the 0.018−3.66 µg range, and the resulting response curves were fitted non-linearly (using OriginPro 2019b from OriginLab Corp.) to a simple power equation.

$$M = k \cdot A^{p\cdot}$$

where M is the injected mass of the compound, A is the area under the peak in the chromatogram, while the fitted parameters k and p are referred to as the coefficient and the exponent, respectively. By knowing the exact dilution factor of the sampled aliquot and the injection volume used on the

chromatographic equipment, the calculated injected mass of a compound can easily be converted to a concentration by weight in the actual reaction mixture.

The values of *k* and *p* obtained from these fits, along with the errors on the fits and the resulting adjusted R^2-values, are listed in Table S4 below.

Table S4: Values obtained from fit for the calibration of the HPLC-ELSD method.

Species	*k*	*p*	*k err. on fit*	*p err. on fit*	Adj. R2 on fit
***α*-CD**	0.229	0.557	0.002	0.003	0.998
***β*-CD**	0.237	0.557	0.003	0.004	0.997
***γ*-CD**	0.257	0.551	0.002	0.002	0.999
**G1**	0.241	0.556	0.002	0.002	0.999
**G2**	0.300	0.543	0.002	0.002	1.000
**G3**	0.283	0.553	0.002	0.002	0.999
**G4**	0.283	0.553			
**G5**	0.283	0.553			
**G6**	0.283	0.553			
**G7**	0.283	0.553			
**G8**	0.283	0.553			

S5.2 DCL distributions obtained from HPLC chromatography

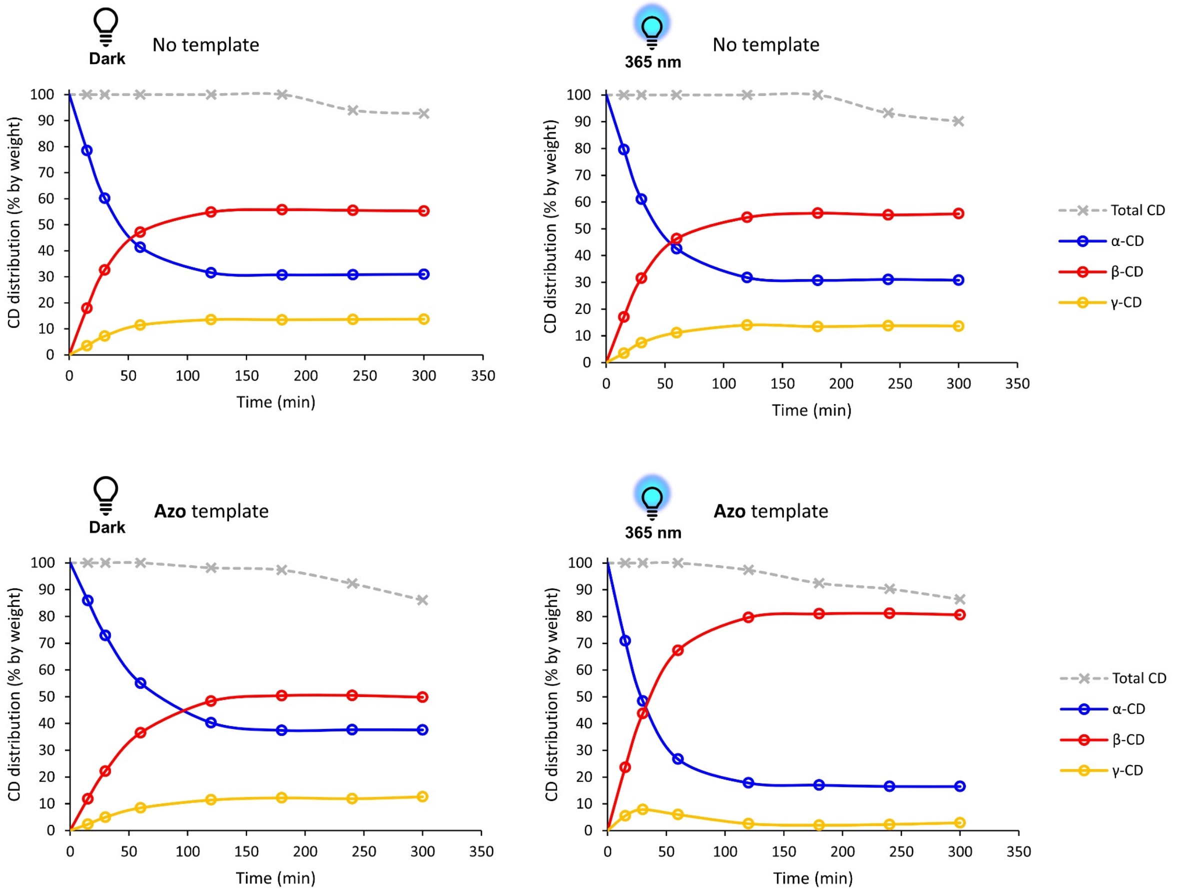

Figure S17: Top: distribution of α-, β-, and γ-CD and total CD yield over 5 hours for the untemplated DCL performed in darkness (left) and under irradiation with at 365 nm (right). Bottom: distribution of α-, β-, and γ-CD and total CD yield over 5 hours for the DCL templated with **Azo** performed in darkness (left) and under irradiation with UV light at 365 nm (right). Conditions: the DCLs were performed using 10 mg/mL α-CD with and without 10 mM template in 100 mM phosphate buffer at pH 7.5 and at room temperature. For reactions performed in darkness the reaction mixture was stored overnight at 30 °C in the dark prior to starting the reaction by addition of enzyme. For the irradiated reactions, the reaction mixture was pre-irradiated to obtain the photostationary state of the template before starting the reaction by addition of enzyme.

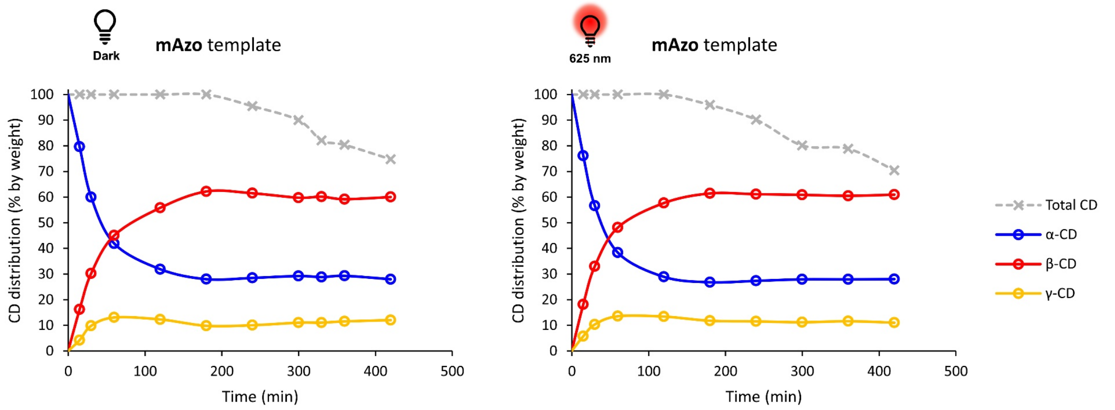

Figure S18: Distribution of α-, β-, and γ-CD and total CD yield over 6 hours for the DCLs templated with **mAzo** performed in darkness (left) and under irradiation with red light at 625 nm. Conditions as in Figure S17.

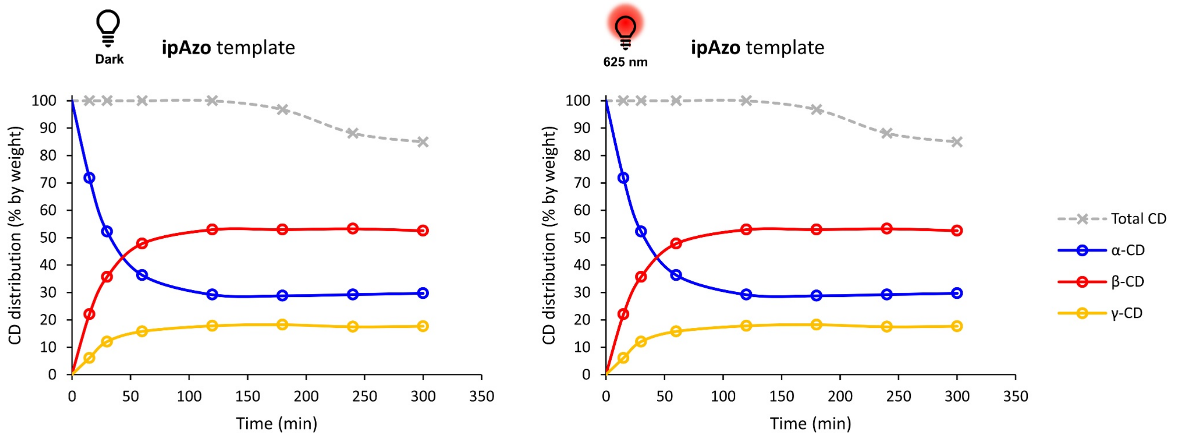

Figure S19: Distribution of α-, β-, and γ-CD and total CD yield over 5 hours for the DCLs templated with **ipAzo** performed in darkness (left) and under irradiation with red light at 625 nm. Conditions as in Figure S17.

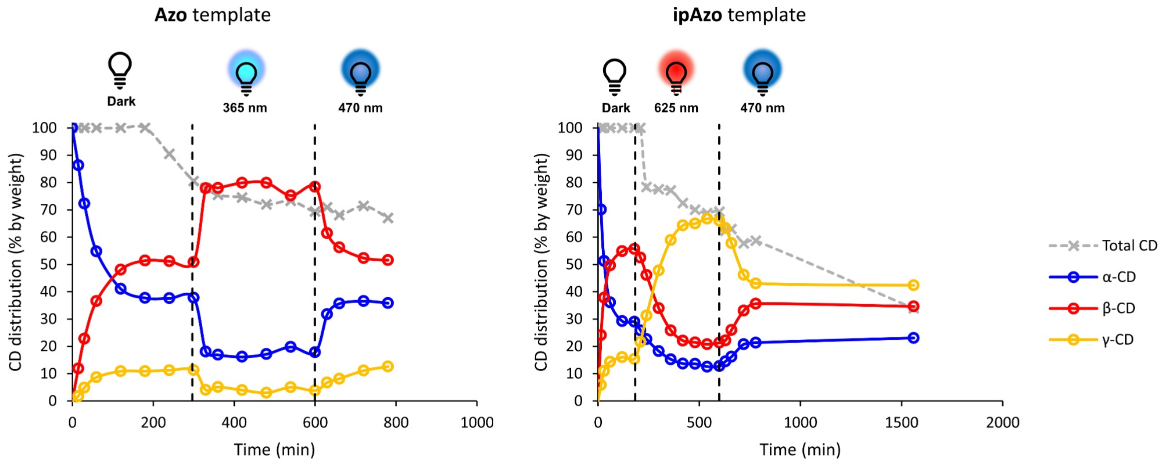

Figure S20: Left**:** distribution of α-, β-, and γ-CD and total CD yield for a DCL templated with **Azo** started in darkness (97% *trans*-**Azo**), then irradiated at 365 nm (to promote *trans* to *cis* isomerisation), and finally irradiated with blue light at 470 nm (to promote *cis* to *trans* isomerisation). Right: distribution of α-, β-, and γ-CD and total CD yield for a DCL templated with **ipAzo** started in darkness (91% *trans*-**ipAzo**), then irradiated at 625 nm (to promote *trans* to *cis* isomerisation), and finally irradiated with at 470 nm (to promote *cis* to *trans* isomerisation). Conditions as in Figure S17.

S5.3 Template recovery and preparative scale templated enzymatic synthesis of γ-CD

Template recovery from enzyme-mediated synthesis of γ-CD monitored by *in situ* NMR spectroscopy

A solution of α-CD (10 mg/mL) and **ipAzo** (10 mM) in sodium phosphate buffered (100 mM, pH 7.5) D_2O was prepared in a colorless 2 mL glass vial with a screw-cap and irradiated with light at 625 nm for 15 hours. CGTase enzyme stock solution (65 µL/mL) was added and the reaction was kept under red light irradiation. To monitor the reaction by ^{1}H NMR spectroscopy, the reaction mixture was transferred to a standard 5 mm borosilicate glass NMR tube. Water suppression was applied using presaturation to obtain useful spectra, since addition of the enzyme stock solution introduces a significant amount of non-deuterated water. After acquisition, the reaction mixture was transferred

back to the glass vial and irradiated with red light. After 5 hours, the enzyme was denatured by installing the NMR tube in a specially fitted aluminium block thermostatically heated to 95 °C for 15 minutes. After cooling to room temperature, an ^{1}H NMR spectrum was acquired. The mixture was transferred to a centrifuge tube and centrifuged at 10000 rpm for 8 minutes in a Heraeus Biofuge Pico to remove enzyme. The supernatant was transferred to another centrifuge tube with 100 μL buffer and 1% (v/v) TFA was added leading to immediate precipitation of a red solid. After centrifugation as just described, the filtrate was transferred to an NMR tube and analysed. The precipitate was suspended in MilliQ water (0.5 mL), centrifuged, the water was decanted off and the precipitate was dried *in vacuo*. The precipitate was redissolved in sodium phosphate buffered (100 mM, pH 7.5) D_2O for NMR analysis. Signals corresponding to α-CD, β-CD, and γ-CD was assigned using reference spectra of α-CD, β-CD, and γ-CD in sodium phosphate buffered (100 mM, pH 7.5) D_2O. Additionally, the supernatant and precipitate were analysed by HPLC (sample preparation as described in section S5.1). The chromatograms are shown below in Figure S21.

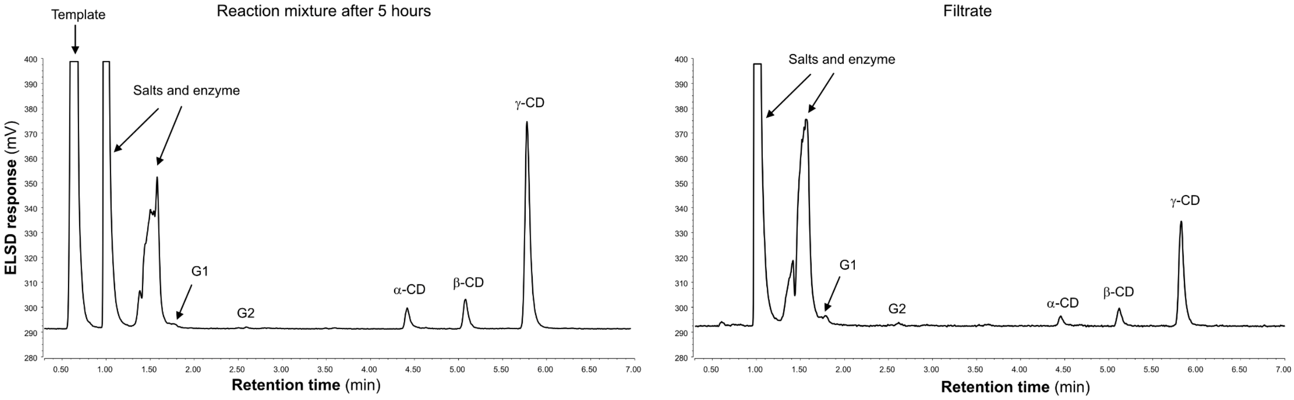

Figure S21: Chromatograms obtained using HPLC-ELSD of (left) the reaction mixture after 5 hours and (right) the filtrate after acidification.

<u>Preparative scale templated enzymatic synthesis of γ-CD</u>

A stock solution of α-CD (11 mg/mL) in sodium phosphate buffered water (100 mM, pH 7.5) was prepared. **ipAzo** (22.0 mg, 0.023 mmol) was dissolved in the α-CD stock solution (2099 μL) to obtain a concentration of 11 mM. This solution was diluted with 94.5 μL buffer and divided equally between two colourless 2 mL glass screw-cap vials. The two vials were irradiated with light at 625 nm for a least 2 hours. With continuous irradiation the enzymatic reaction was started by addition of unfiltered commercial CGTase stock solution (115.5 μL) which was divided equally into the glass vials. In the reaction mixtures a final concentrations were 10 mM **ipAzo**, 10 mg/mL α-CD concentration and 50 μL CGTase stock solution per mL reaction mixture was obtained. After 5 hours, the enzyme was denatured by installing the glass vials in a specially fitted aluminium block thermostatically heated to 95 °C for 15 minutes. After cooling to room temperature, the reaction mixtures were transferred to a 15 mL centrifuge tube and centrifuged at 10000 rpm for 8 minutes in an Eppendorf Centrifuge 5810R to remove enzyme. The supernatant was transferred to another centrifuge tube with 200 μL buffer and 1% (v/v) TFA was added leading to immediate precipitation of the template. The mixture was centrifuged and the supernatant containing glucans was collected by decanting. The sediment was washed with MilliQ water (1 mL), centrifuged again, the supernatant removed, and the sediment dried *in vacuo*. The thus isolated template was used again in another cycle of the same reaction, ensuring the same final concentrations in the reaction mixture. After 5 reaction cycles **ipAzo** was recovered (20.4 mg, 0.021 mmol, 93%, high purity confirmed by ^{1}H NMR, Figure S23). The collected

supernatants containing the glucans from the 5 cycles were combined and concentrated to approximately 1 mL by blowing nitrogen over the surface. The solution was filtered through a syringe filter and injected on a HILIC type HPLC column (XBridge BEH Prep OBD Amide 5 µm from Waters, 19×150 mm) using a Büchi Pure C-850 FlashPrep chromatography system equipped with an ELS detector. Gradient elution from 25% water in acetonitrile to 35% water in acetonitrile over 10 minutes and then from 35% acetonitrile in water to 45% acetonitrile in water over 35 minutes was used. Fractions eluting from 36 to 39 minutes contained only γ-CD, and these were combined, concentrated *in vacuo* and lyophilised to yield γ-CD (36.2 mg, 32% yield of glucan starting material). The reaction cycles and the isolation of the γ-CD were analysed by HPLC and a shown in Figure S22.

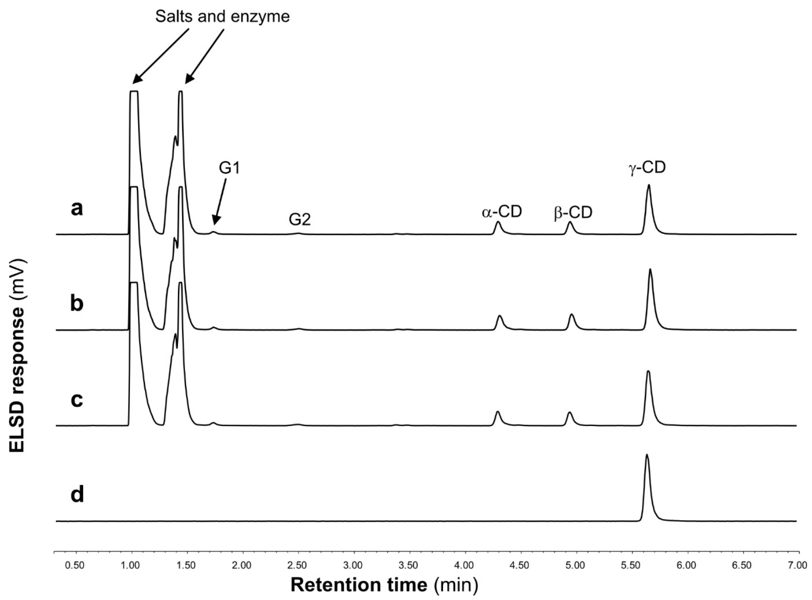

Figure S22: HPLC-ELD chromatograms from the templated enzymatic synthesis of γ-CD. a) Supernatant from reaction cycle 1. b) Supernatant from reaction cycle 3. c) Supernatant from reaction cycle 5. d) Isolated γ-CD.

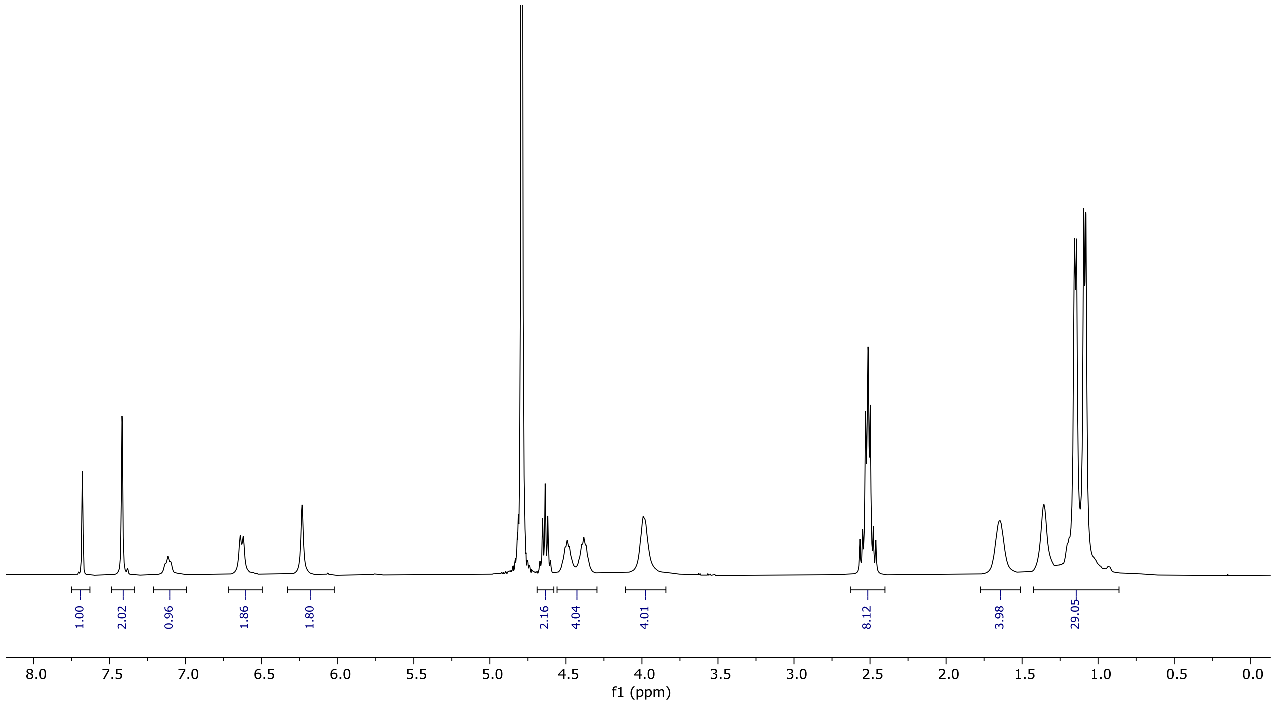

Figure S23: ^{1}H NMR spectrum (400 MHz) of template **ipAzo** in phosphate buffered D_2O (100 mM, pH 7.5) after five reaction cycles of templated enzymatic synthesis of γ-CD.

S6. NMR spectra of synthesised compounds

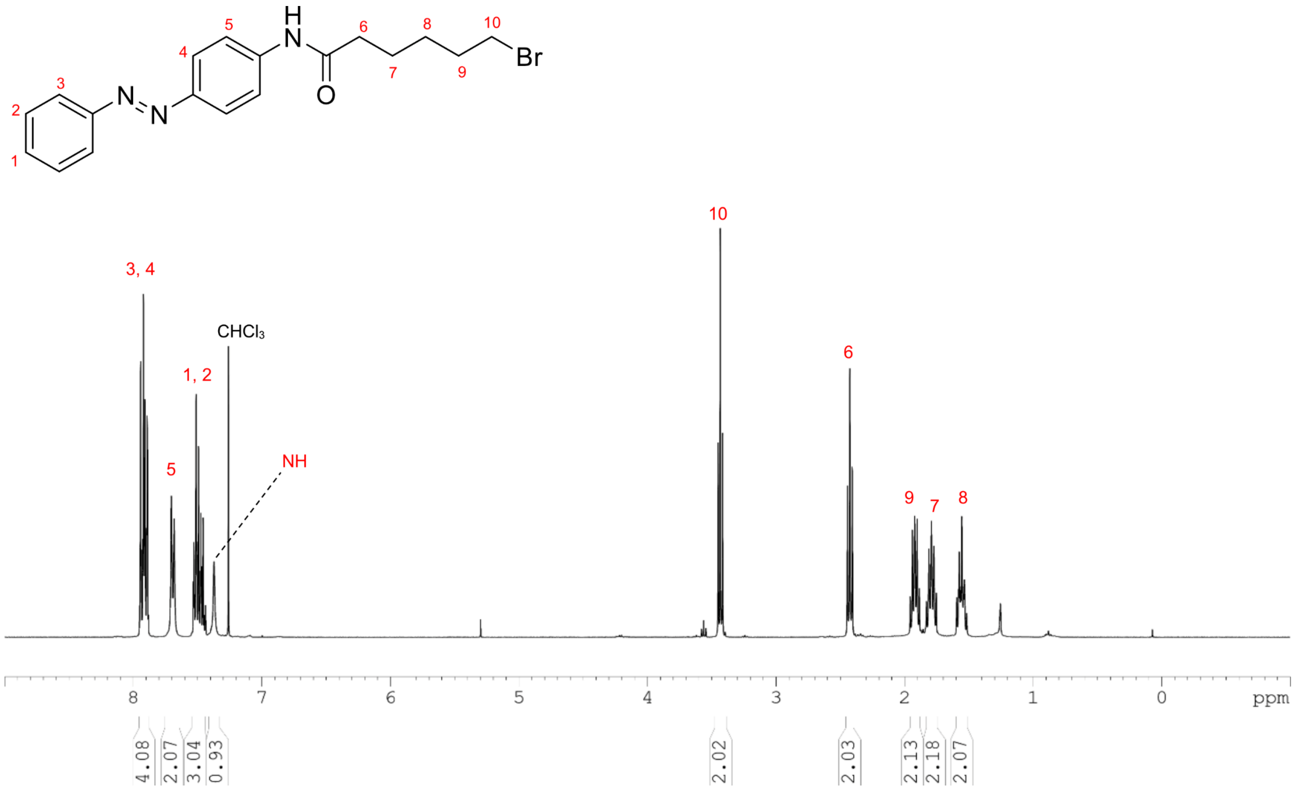

Figure S24: ^{1}H NMR spectra (400 MHz) of compound **6** in chloroform-*d* at 298 K.

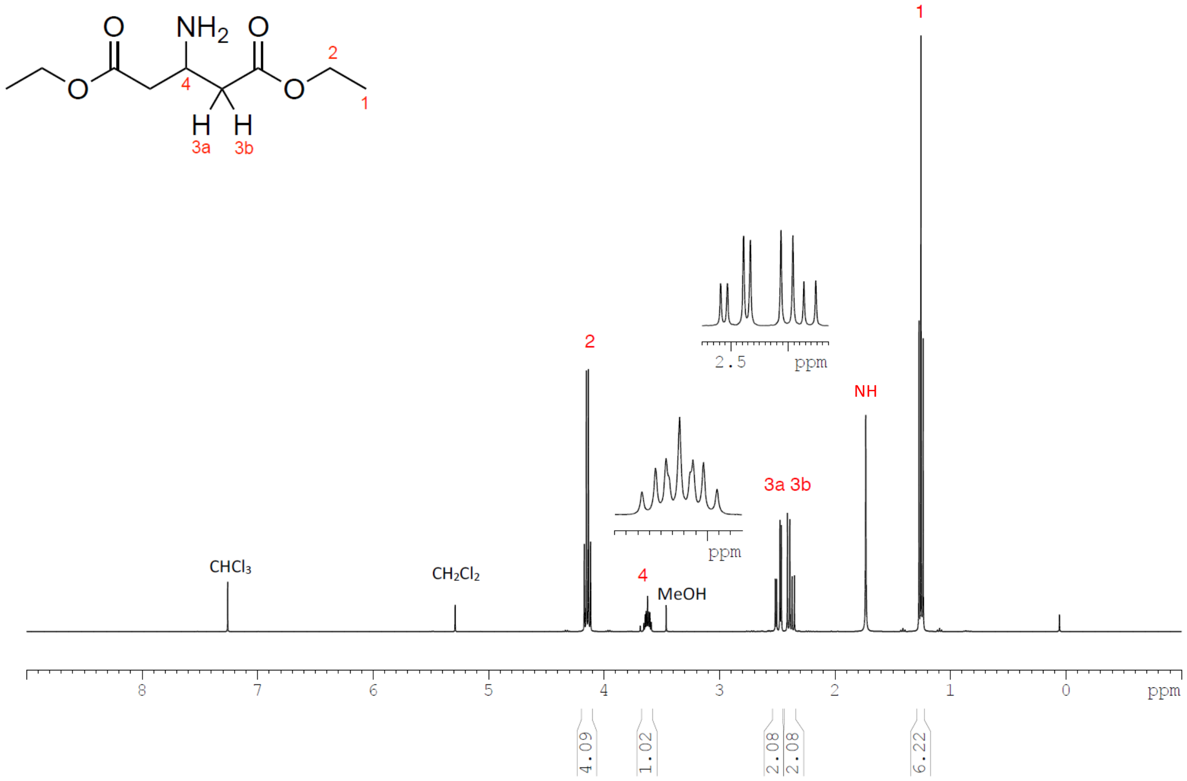

Figure S25: ^{1}H NMR spectrum of compound **4** in chloroform-*d* at 298 K.

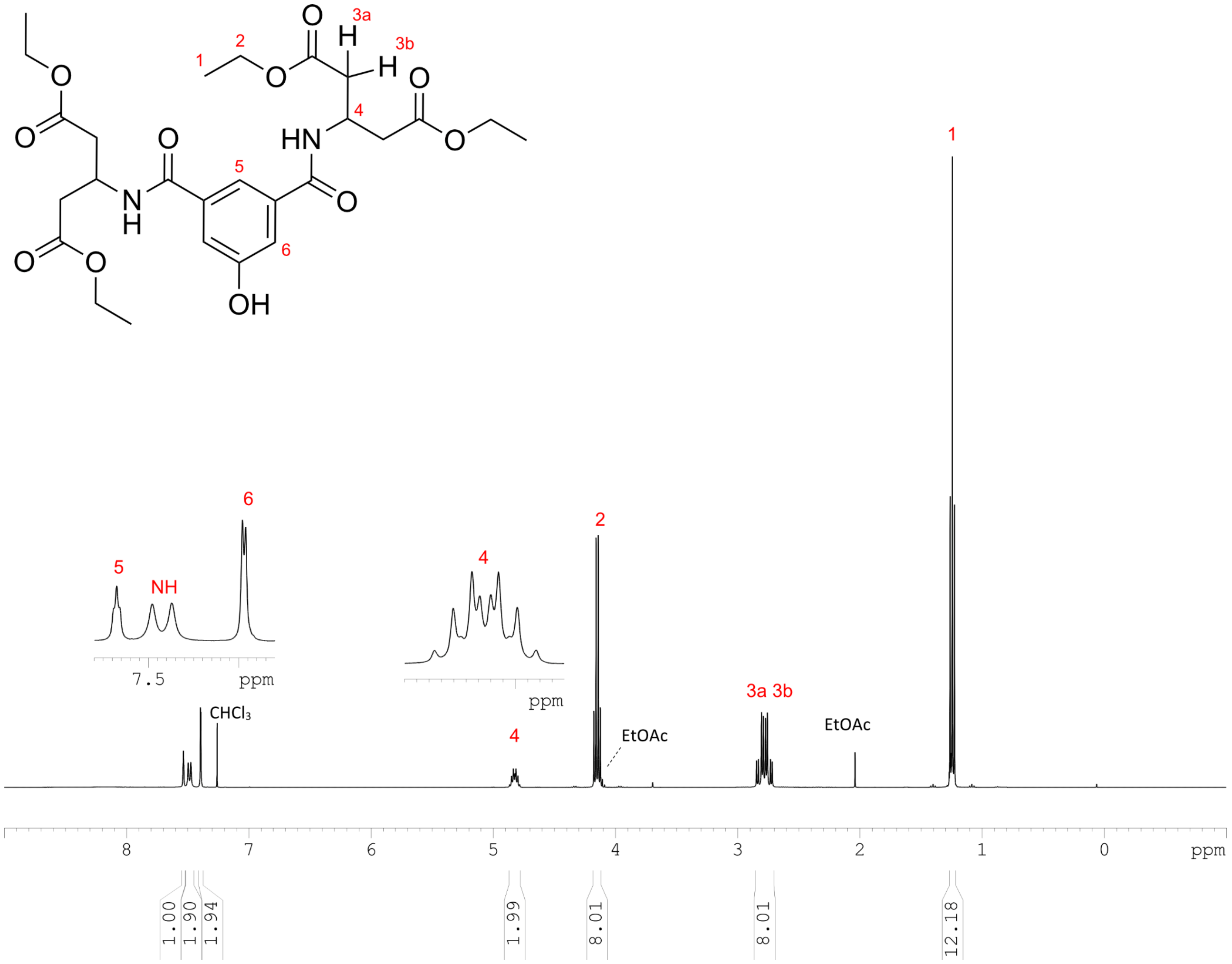

Figure S26: ^{1}H NMR spectrum of compound **5** in chloroform-*d* at 298 K.

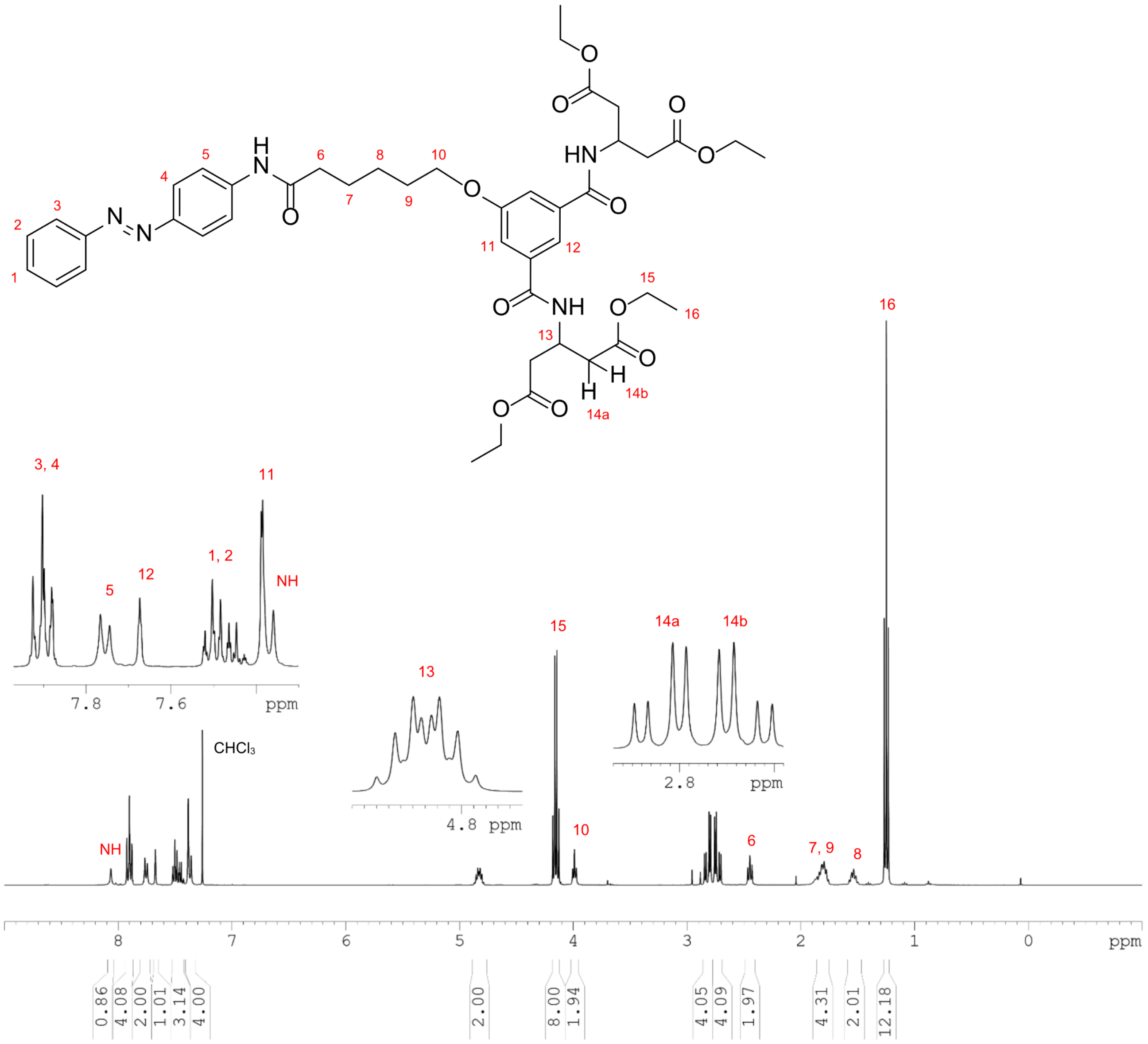

Figure S27: ^{1}H NMR spectrum of compound **7** in chloroform-*d* at 298 K.

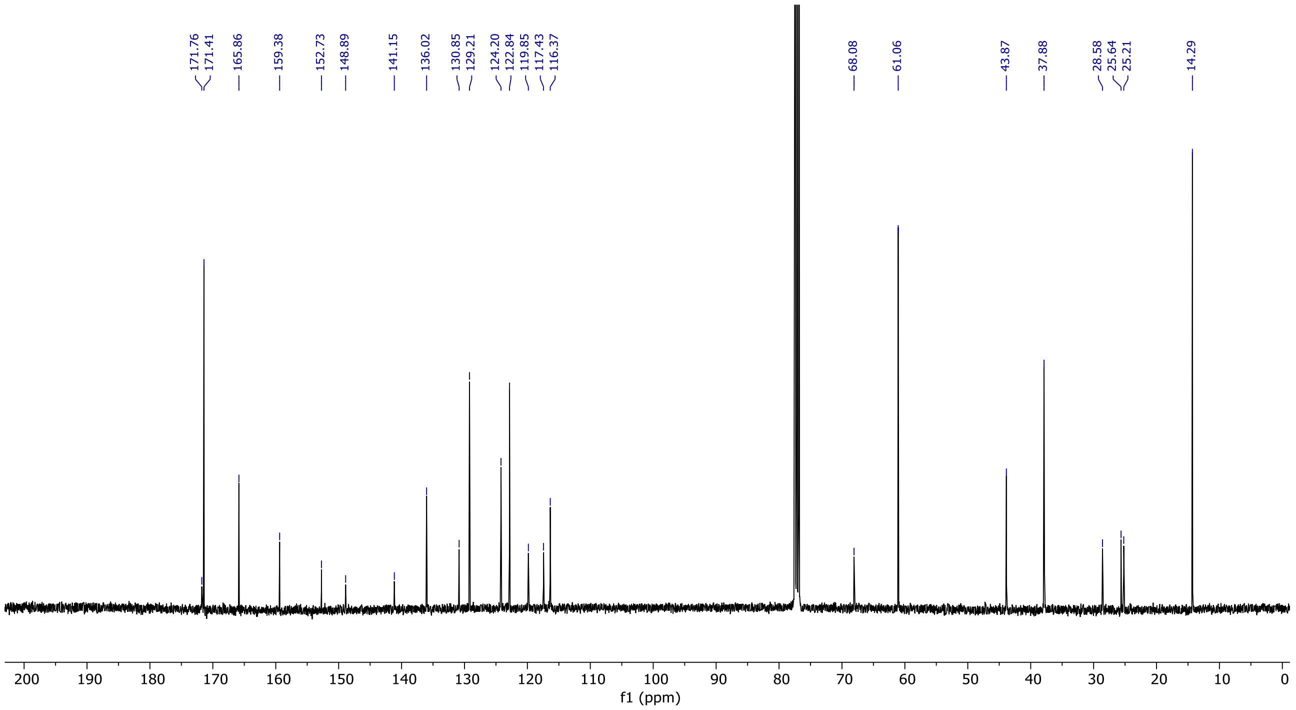

Figure S28: ^{13}C NMR spectrum of compound **7** in chloroform-*d* at 298 K.

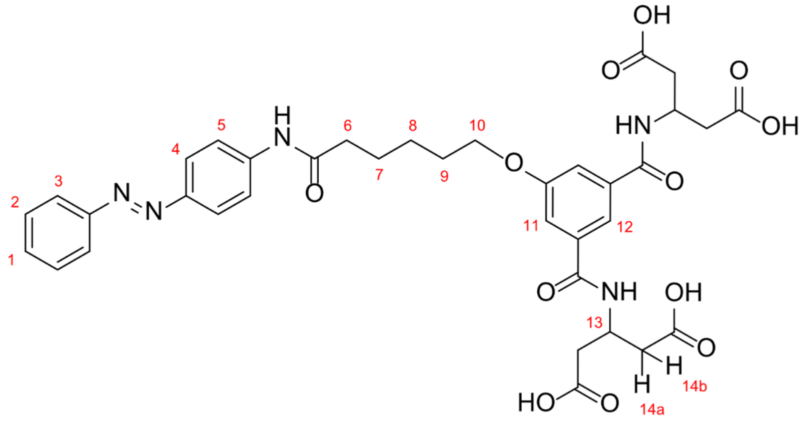

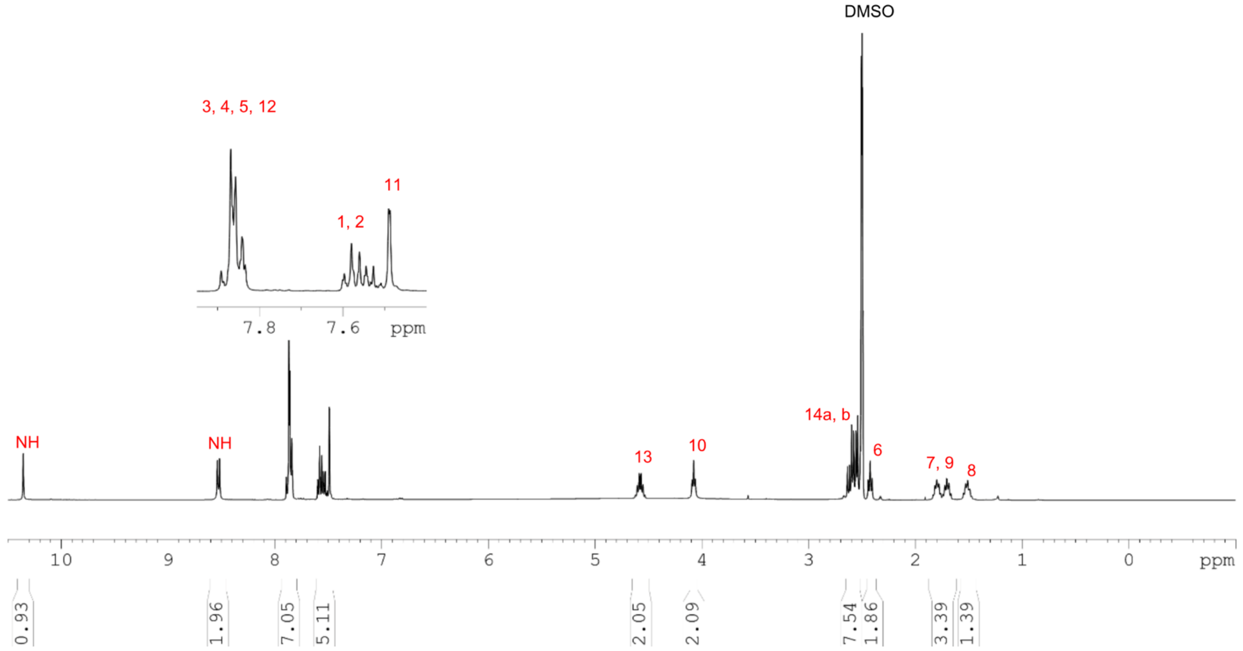

Figure S29: ^{1}H NMR spectrum of template **Azo** in DMSO-d_6 at 298 K.

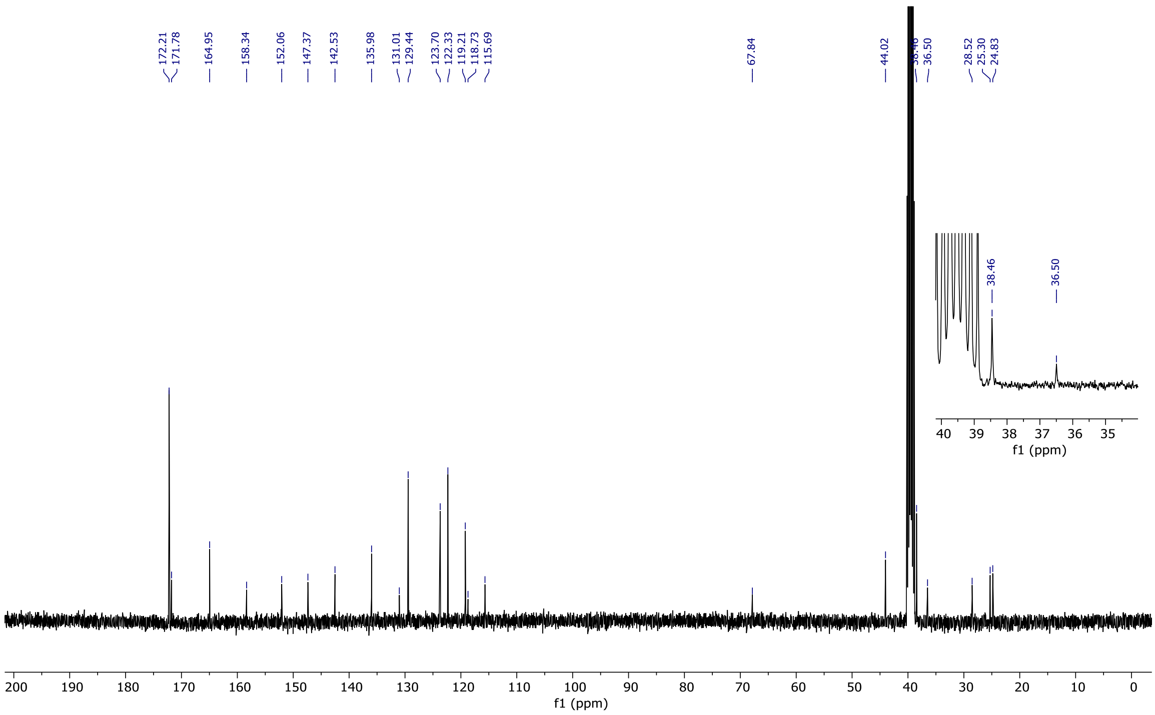

Figure S30: ^{13}C NMR spectrum of template **Azo** in DMSO-d_6 at 298 K.

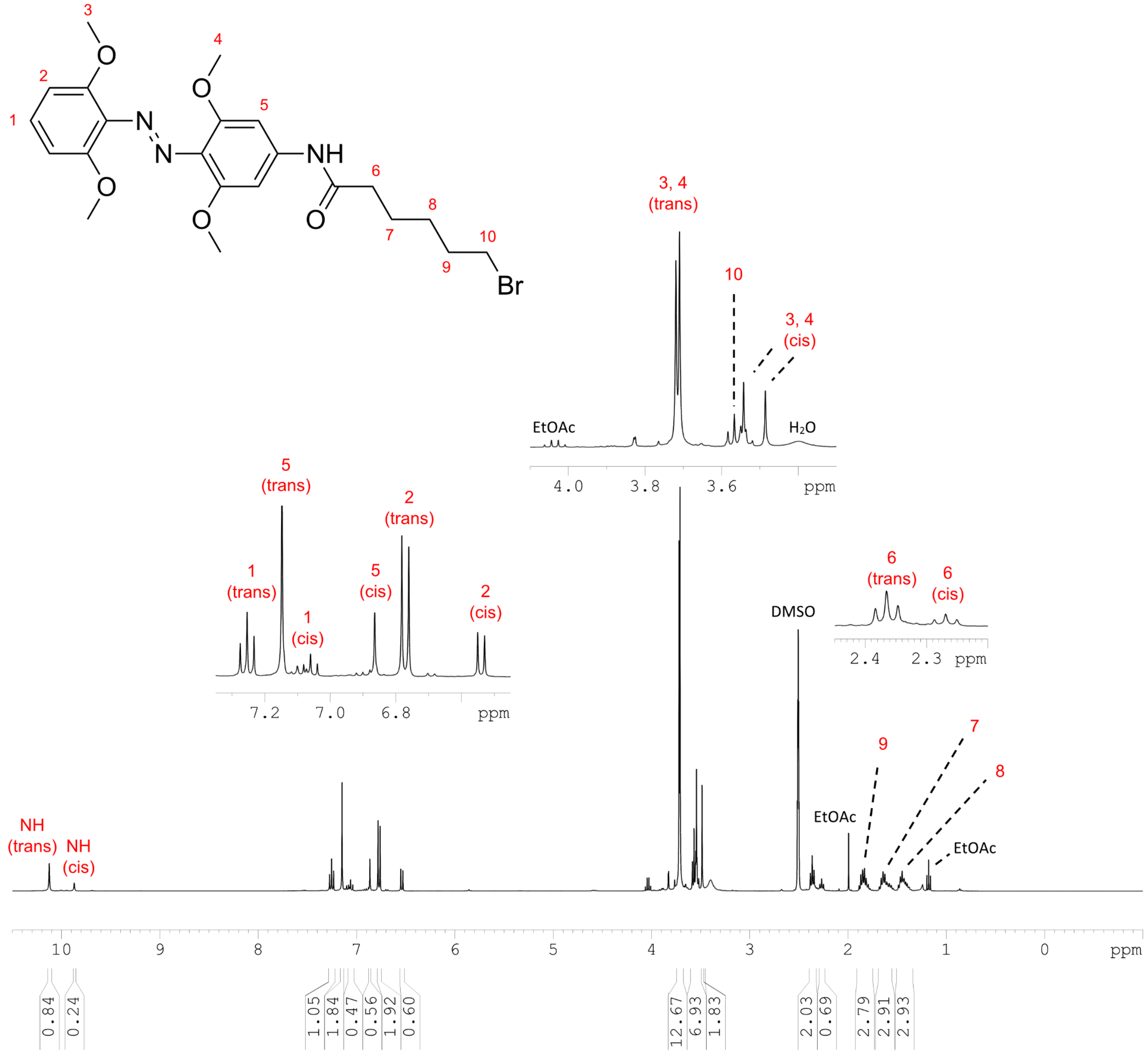

Figure S31: ^{1}H NMR spectrum of compound **9** in DMSO-d_6 at 298 K.

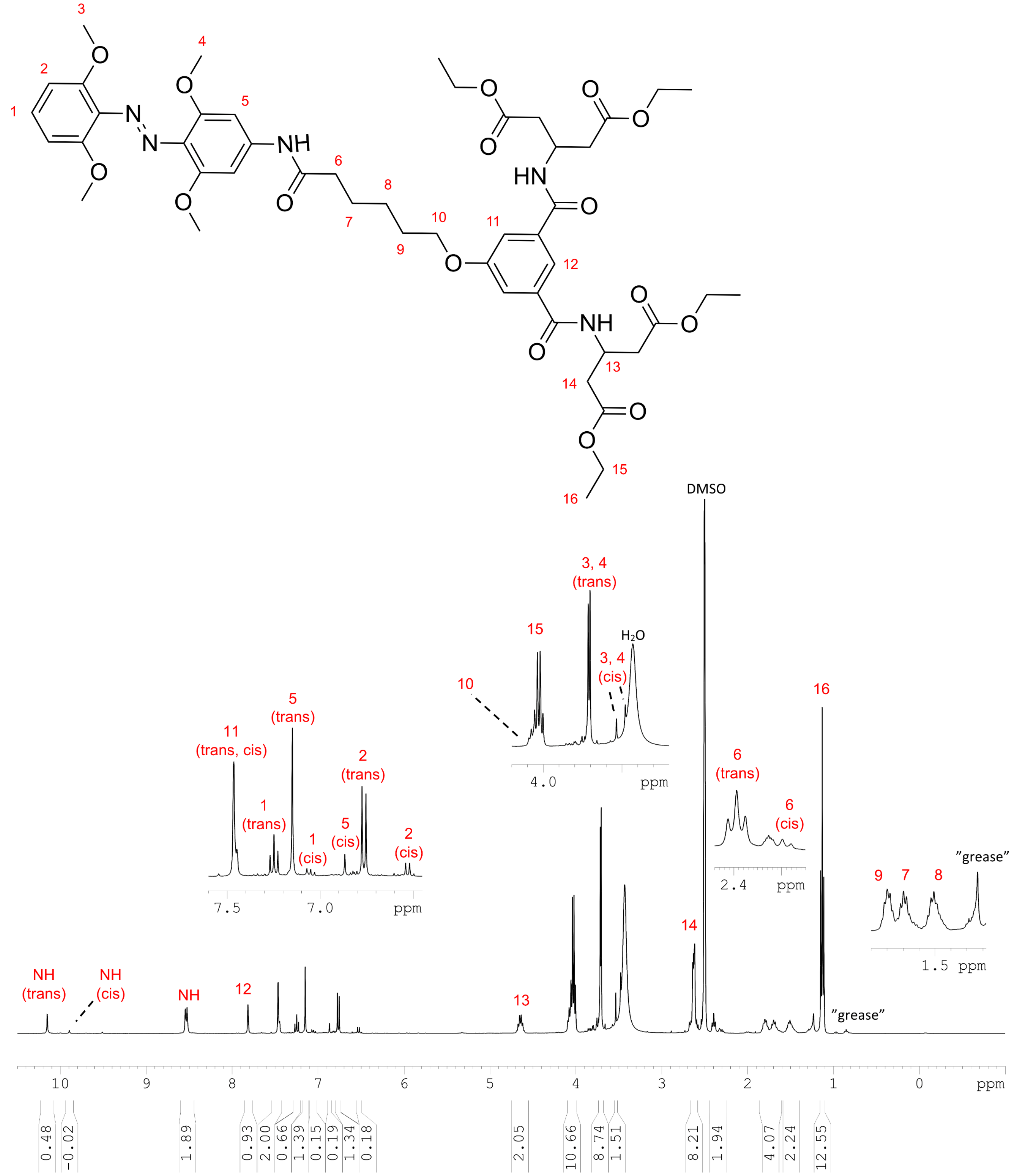

Figure S32: ^{1}H NMR spectrum of compound **10** in DMSO-d_6 at 298 K.

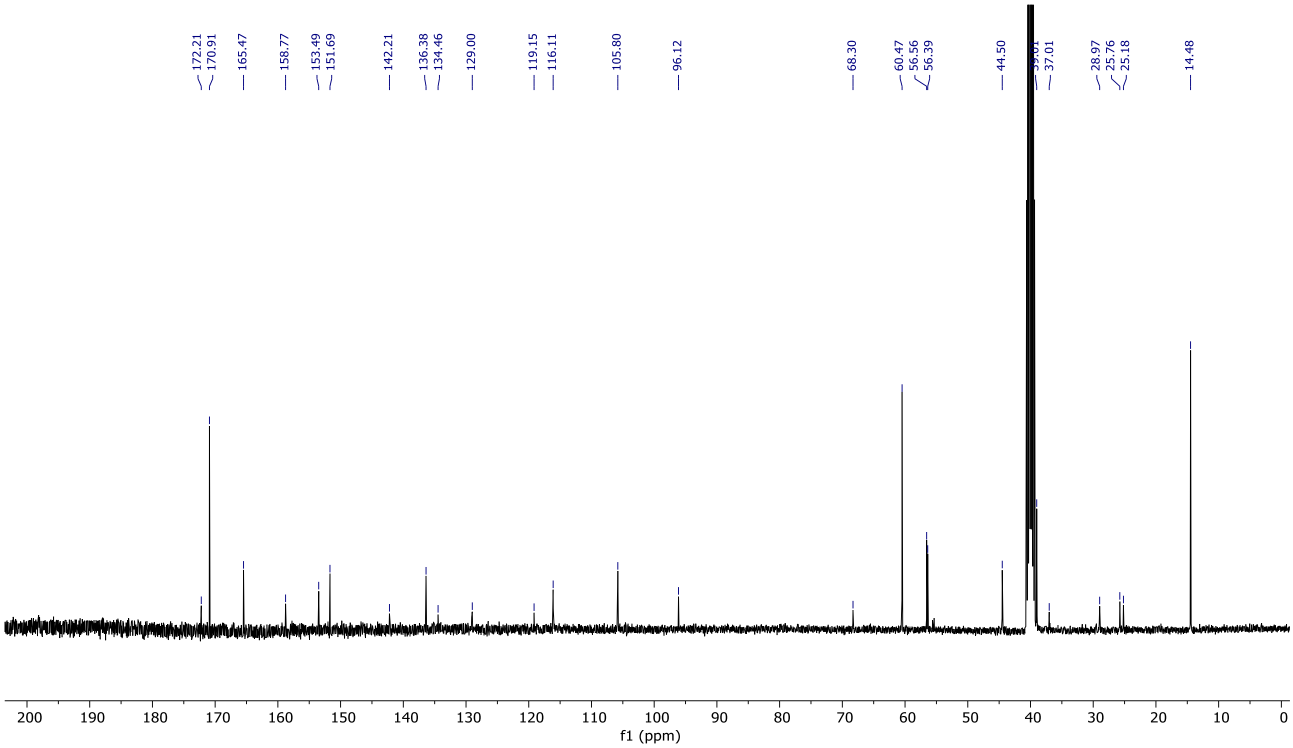

Figure S33: ^{13}C NMR spectrum of compound **10** in DMSO-d_6 at 298 K.

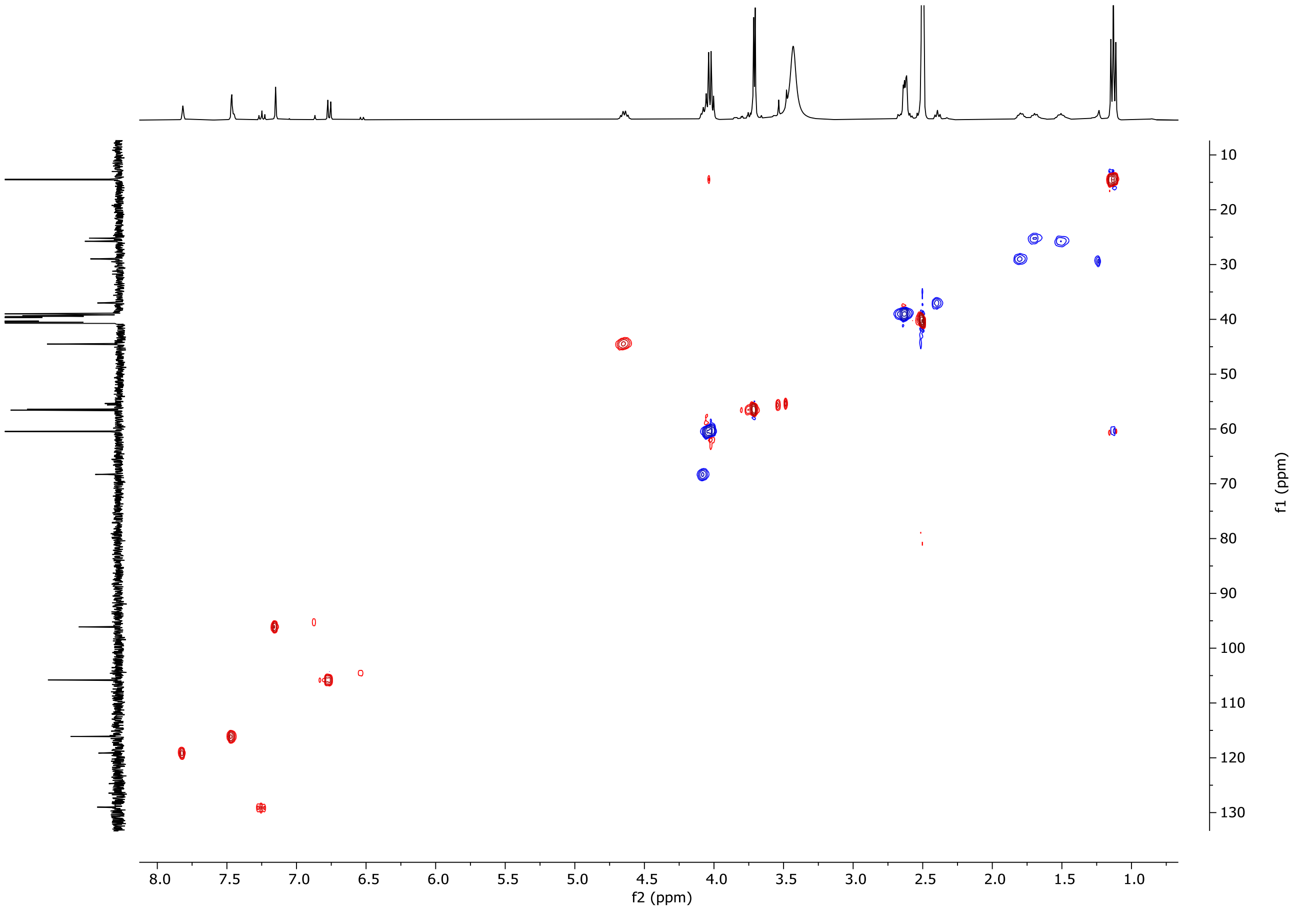

Figure S34: ^{1}H–^{13}C HSQC spectrum of compound **10** in DMSO-d_6 at 298 K.

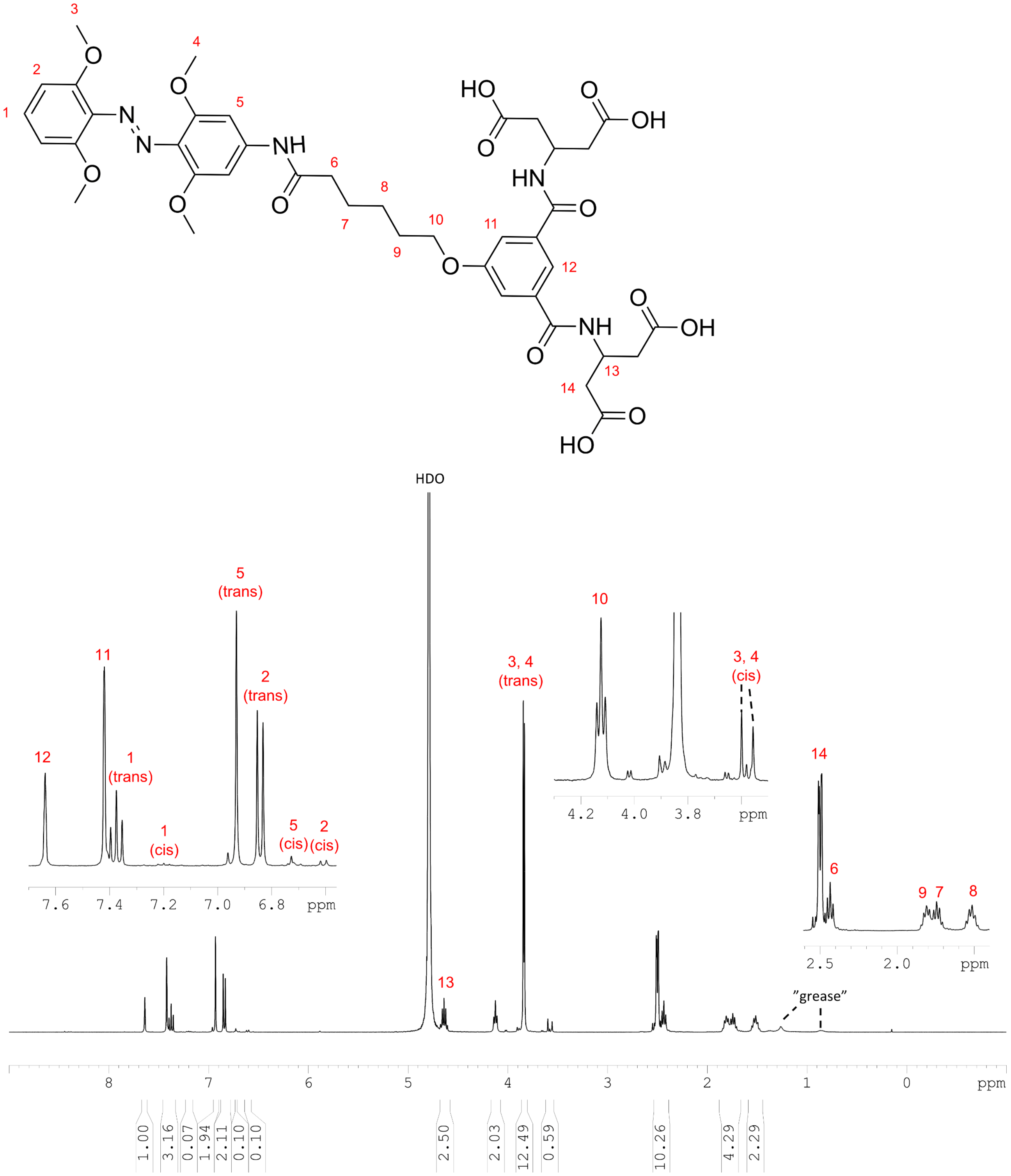

Figure S35: ^{1}H NMR spectrum of template **mAzo** in 100 mM sodium phosphate buffered D_2O at pH 7.5 at 298 K.

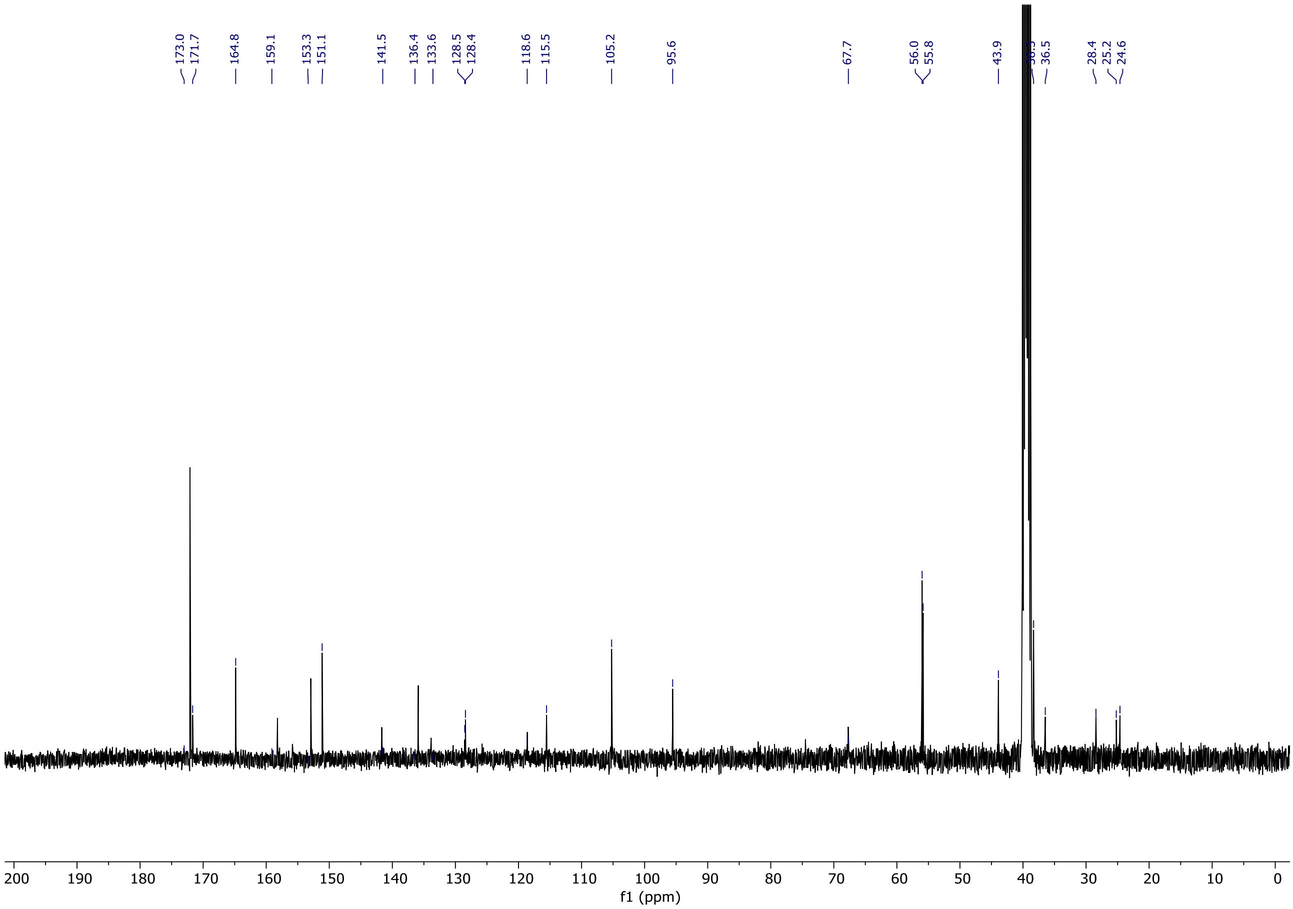

Figure S36: ^{13}C NMR spectrum of template **mAzo** in DMSO-d_6 at 298 K.

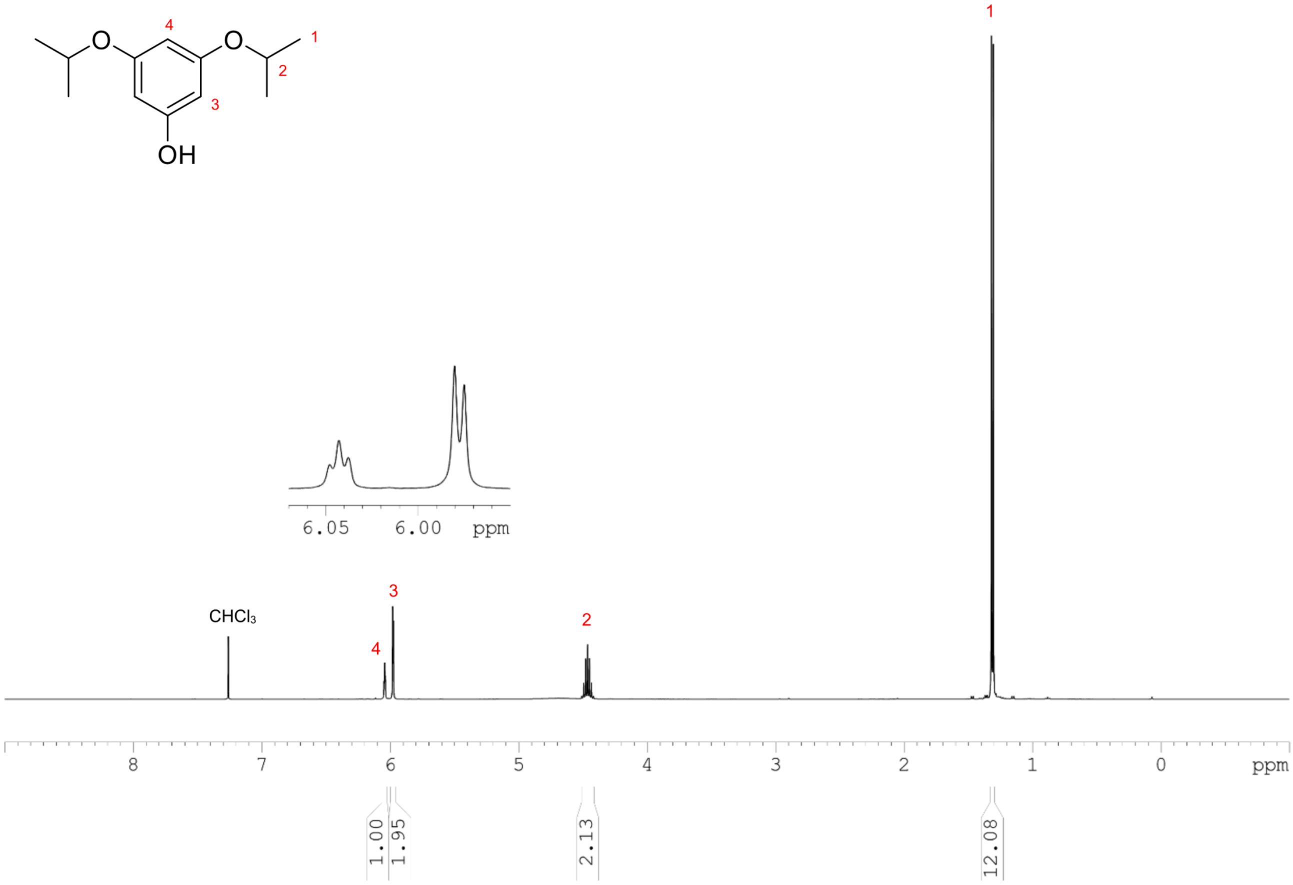

Figure S37: ^{1}H NMR spectrum of compound **1** in chloroform-*d* at 298 K.

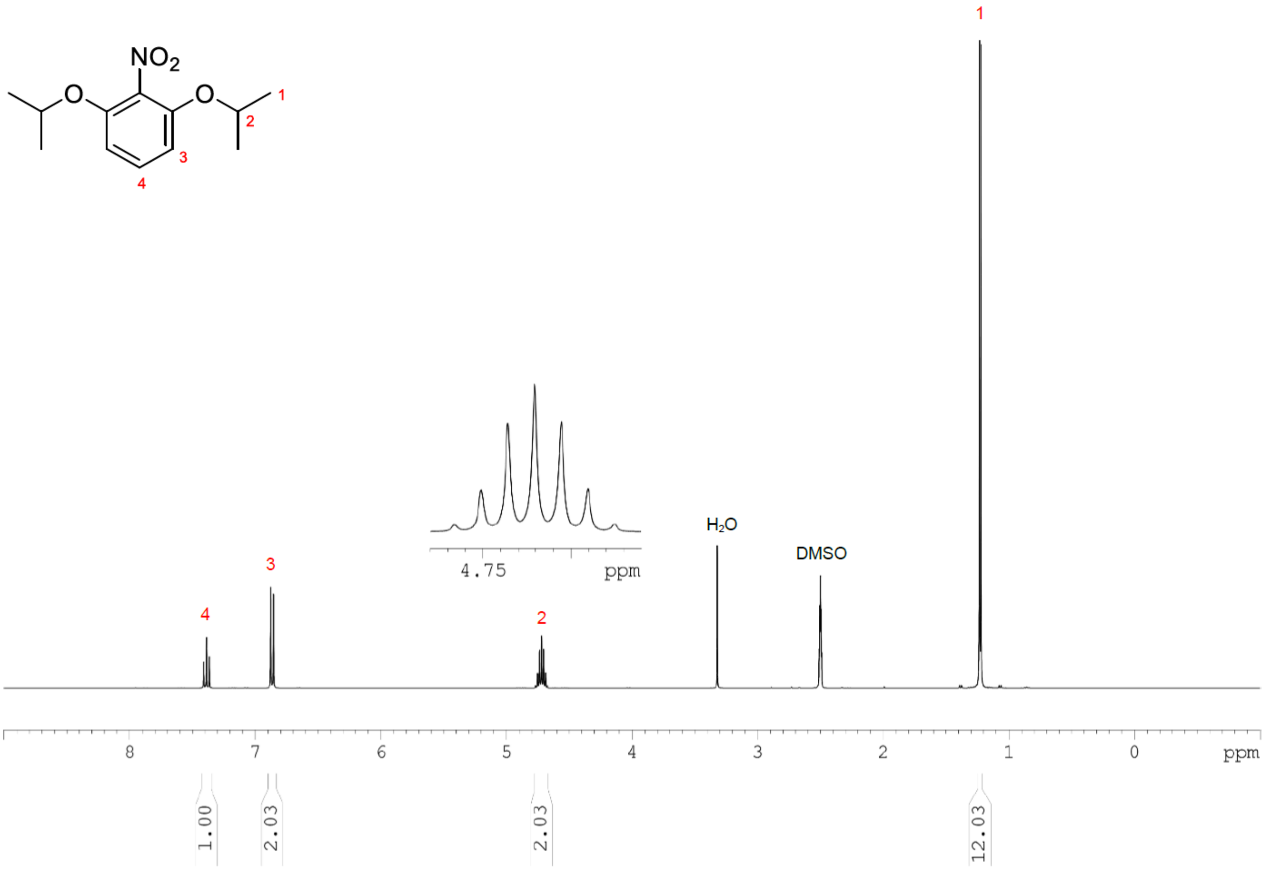

Figure S38: ^{1}H NMR spectrum of compound **11** in DMSO-*d*$_6$ at 298 K.

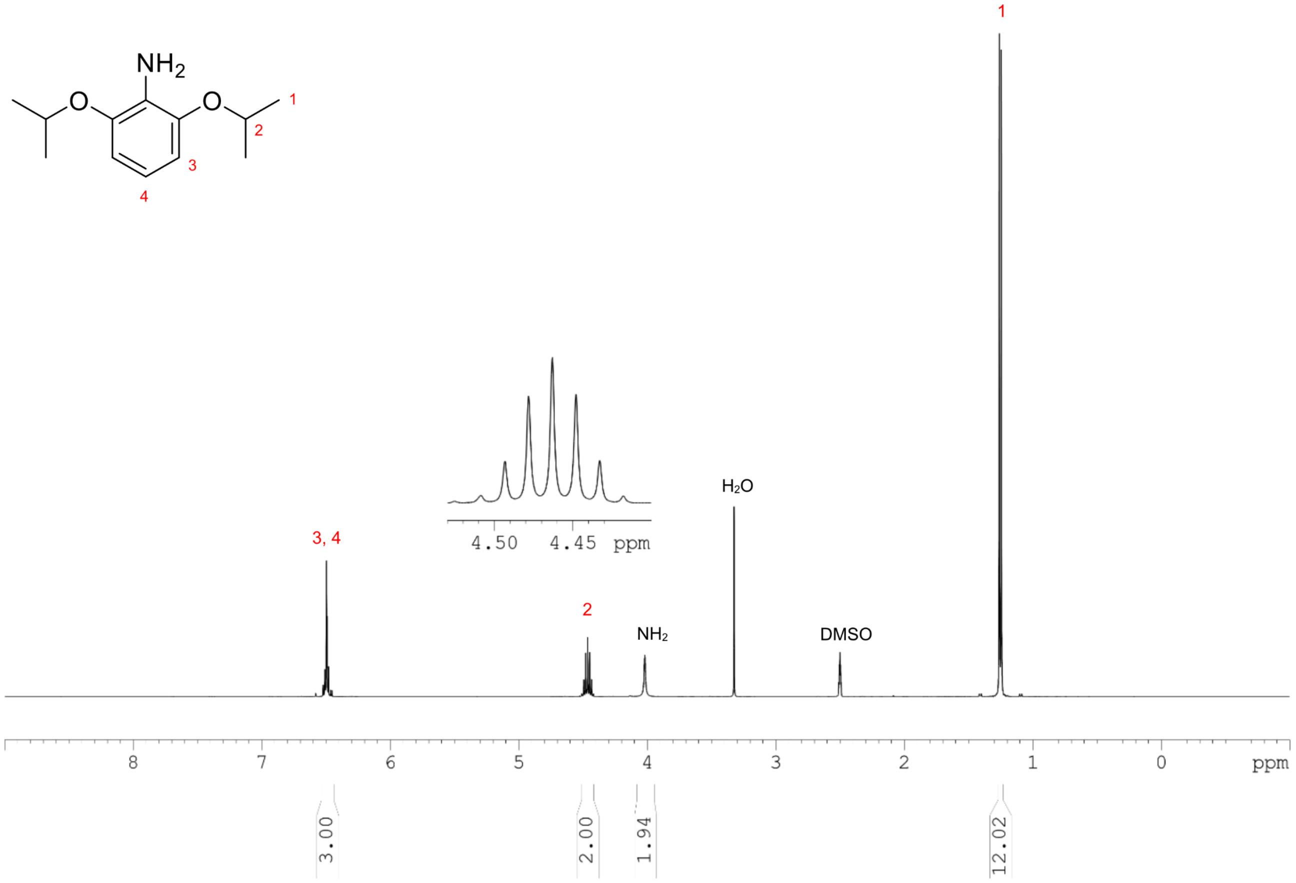

Figure S39: ^{1}H NMR spectrum of compound **2** in DMSO-d_6 at 298 K.

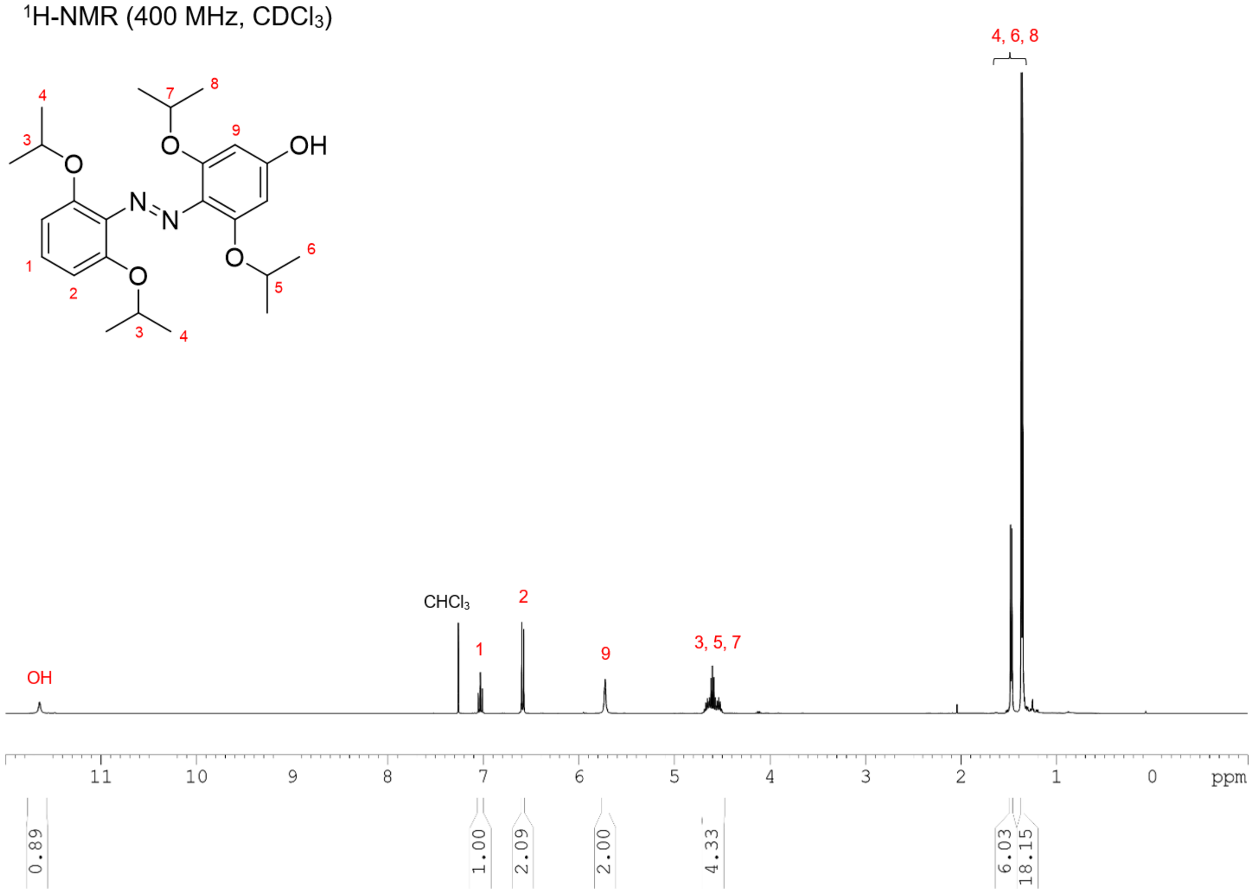

Figure S40: ^{1}H NMR spectrum of compound **3** in chloroform-*d* at 298 K.

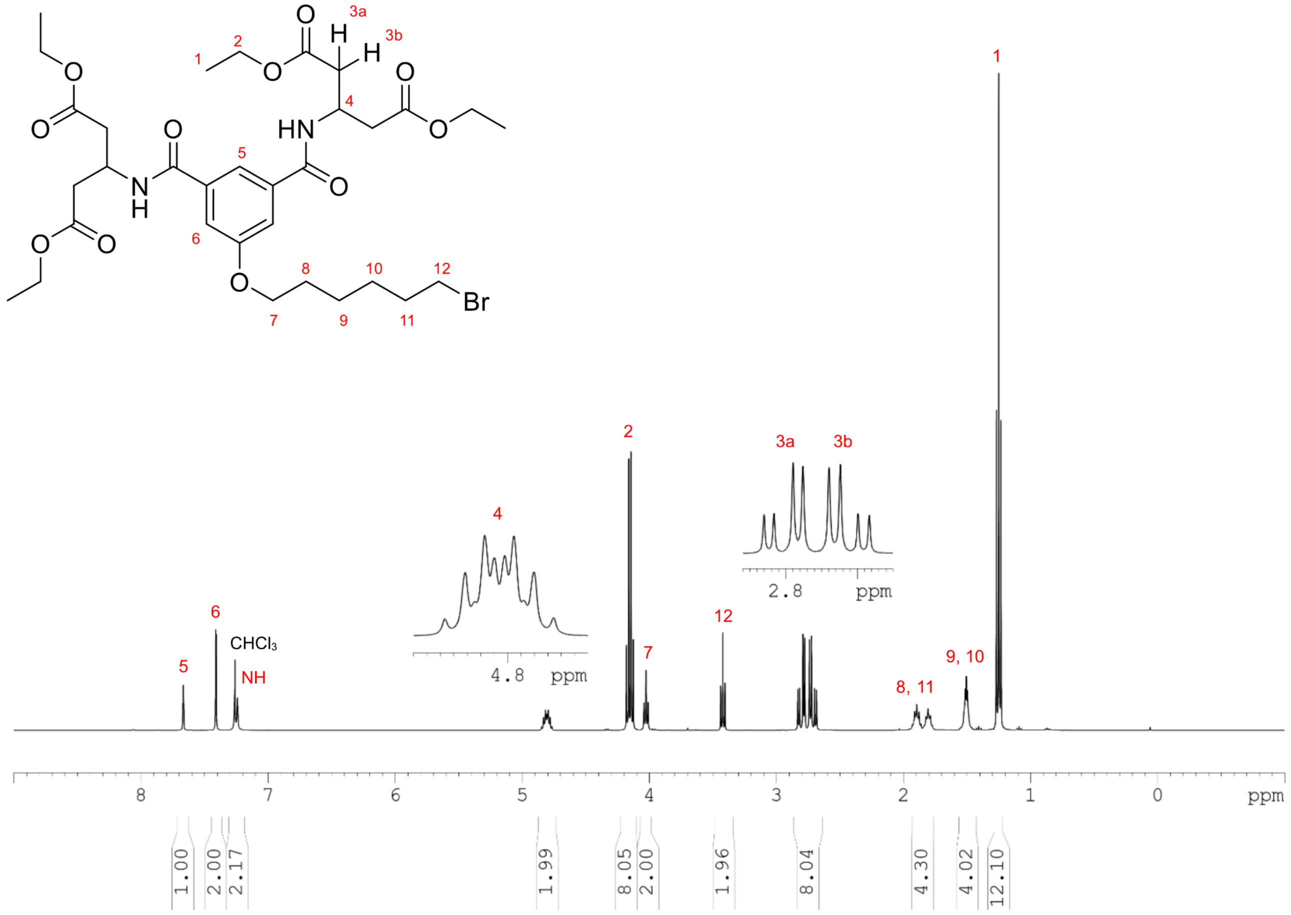

Figure S41: ^{1}H NMR spectrum of compound **12** in chloroform-*d* at 298 K.

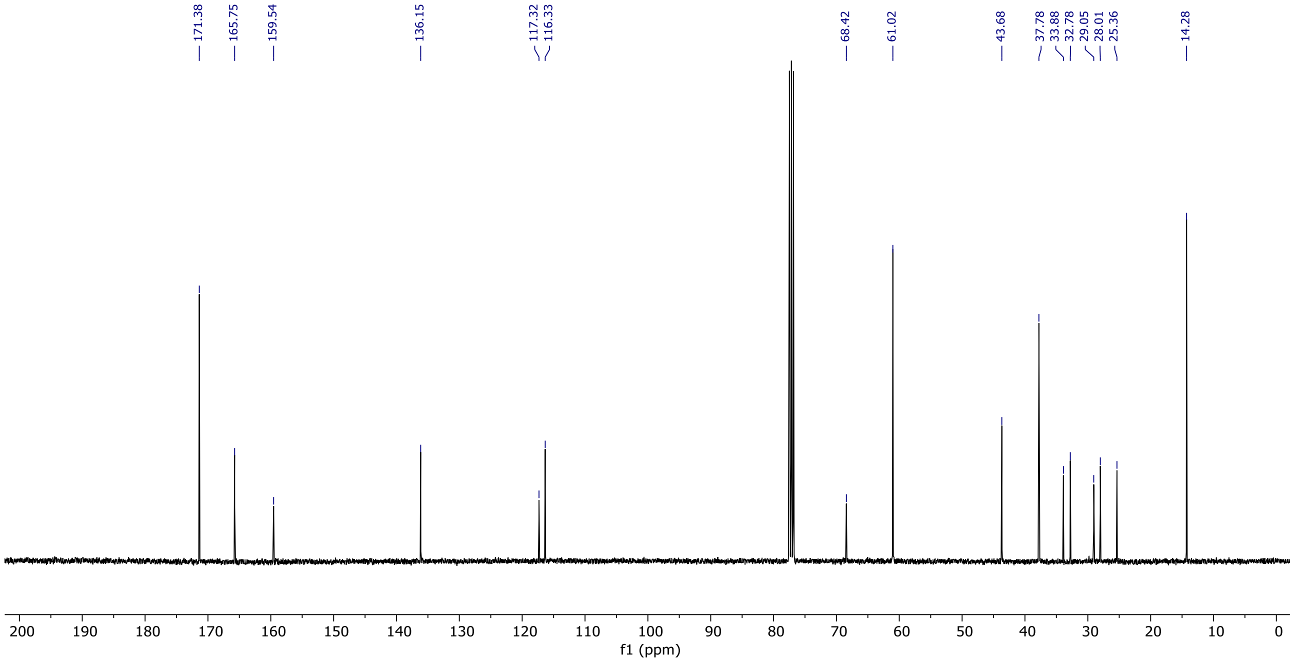

Figure S42: ^{13}C NMR spectrum of compound **12** in chloroform-*d* at 298 K.

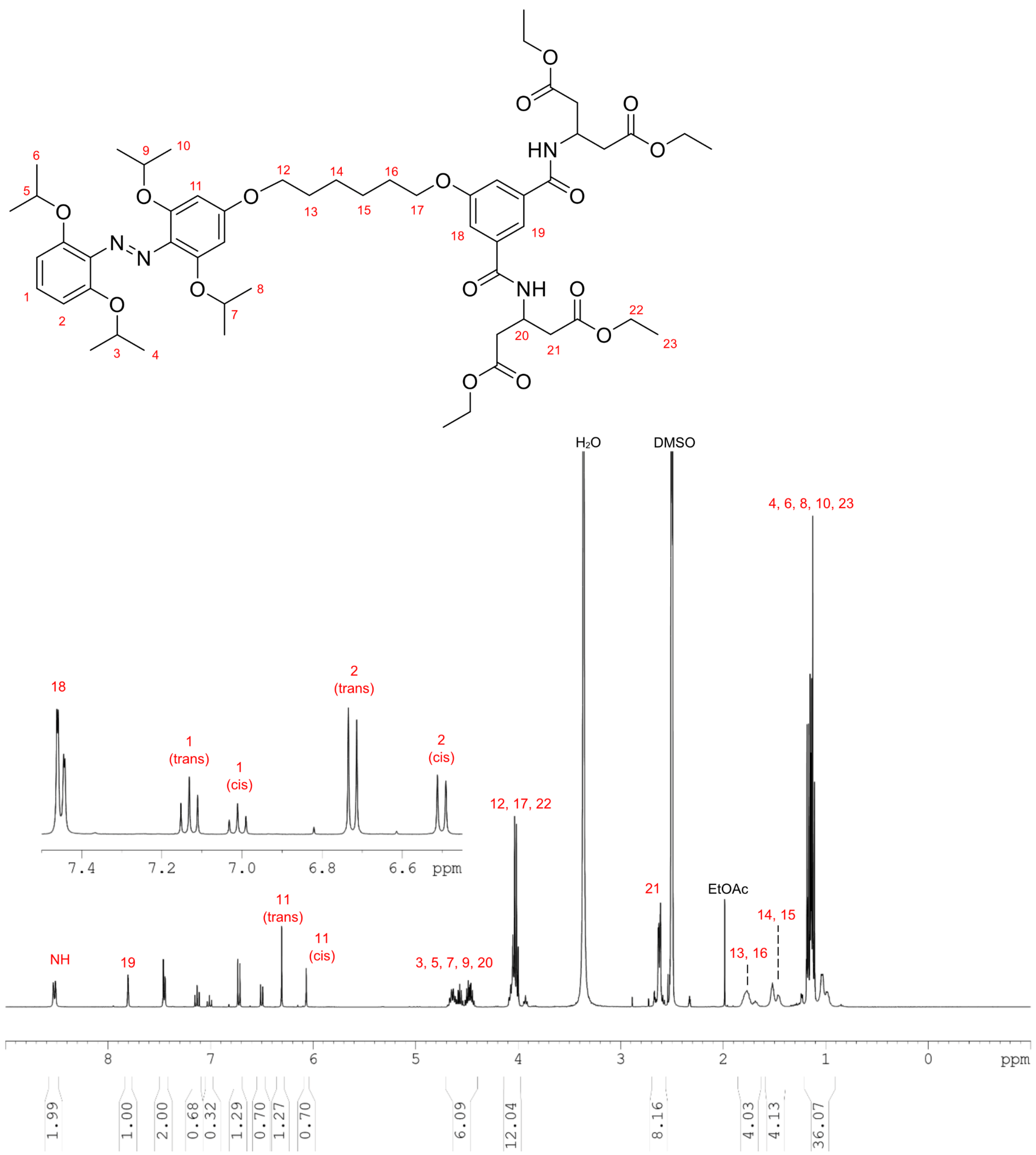

Figure S43: ^{1}H NMR spectrum of compound **13** in DMSO-d_6 at 298 K.

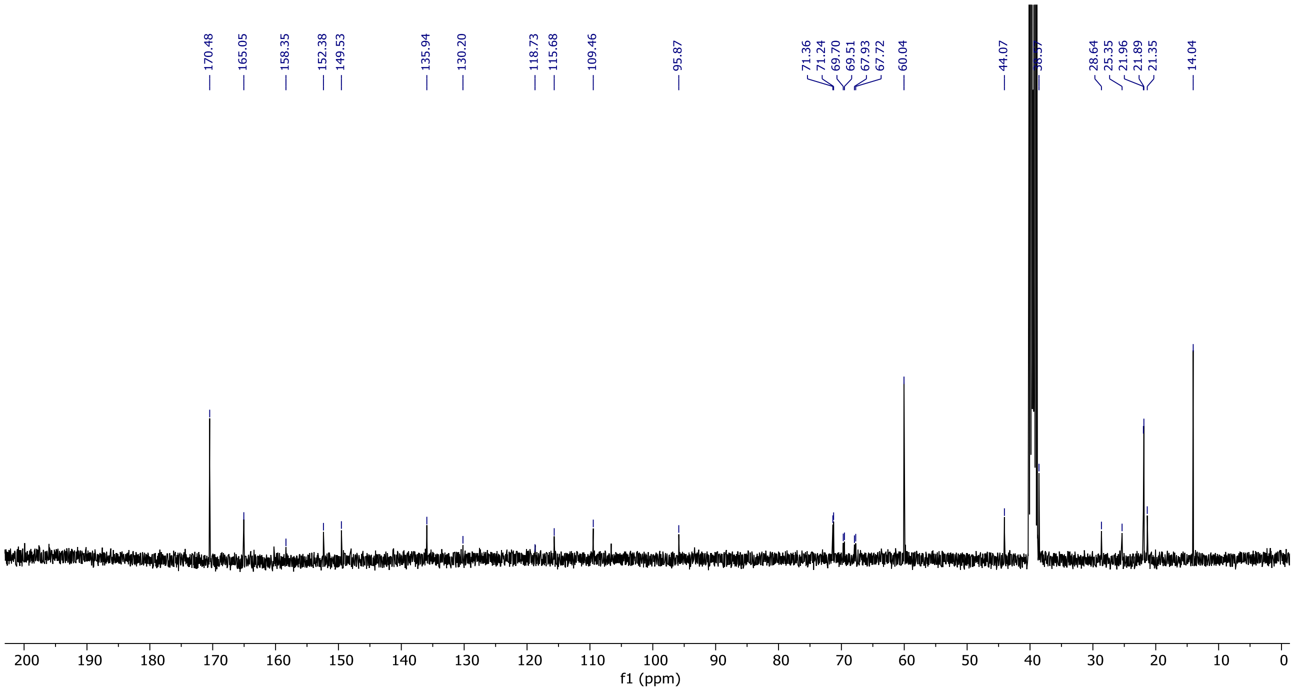

Figure S44: ^{13}C NMR spectrum of compound **13** in DMSO-d_6 at 298 K.

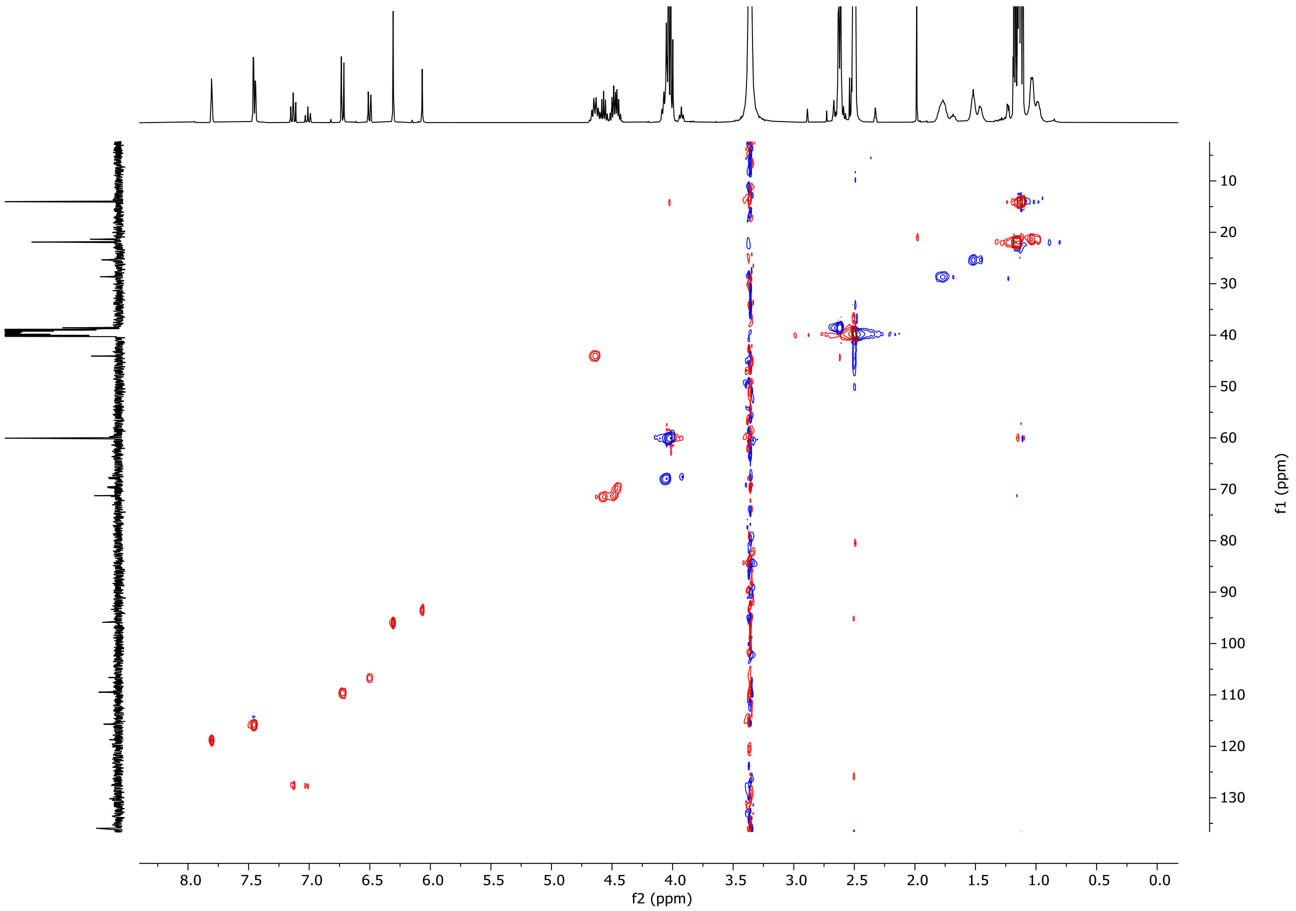

Figure S45: ^{1}H–^{13}C HSQC spectrum of compound **13** in DMSO-d_6 at 298 K.

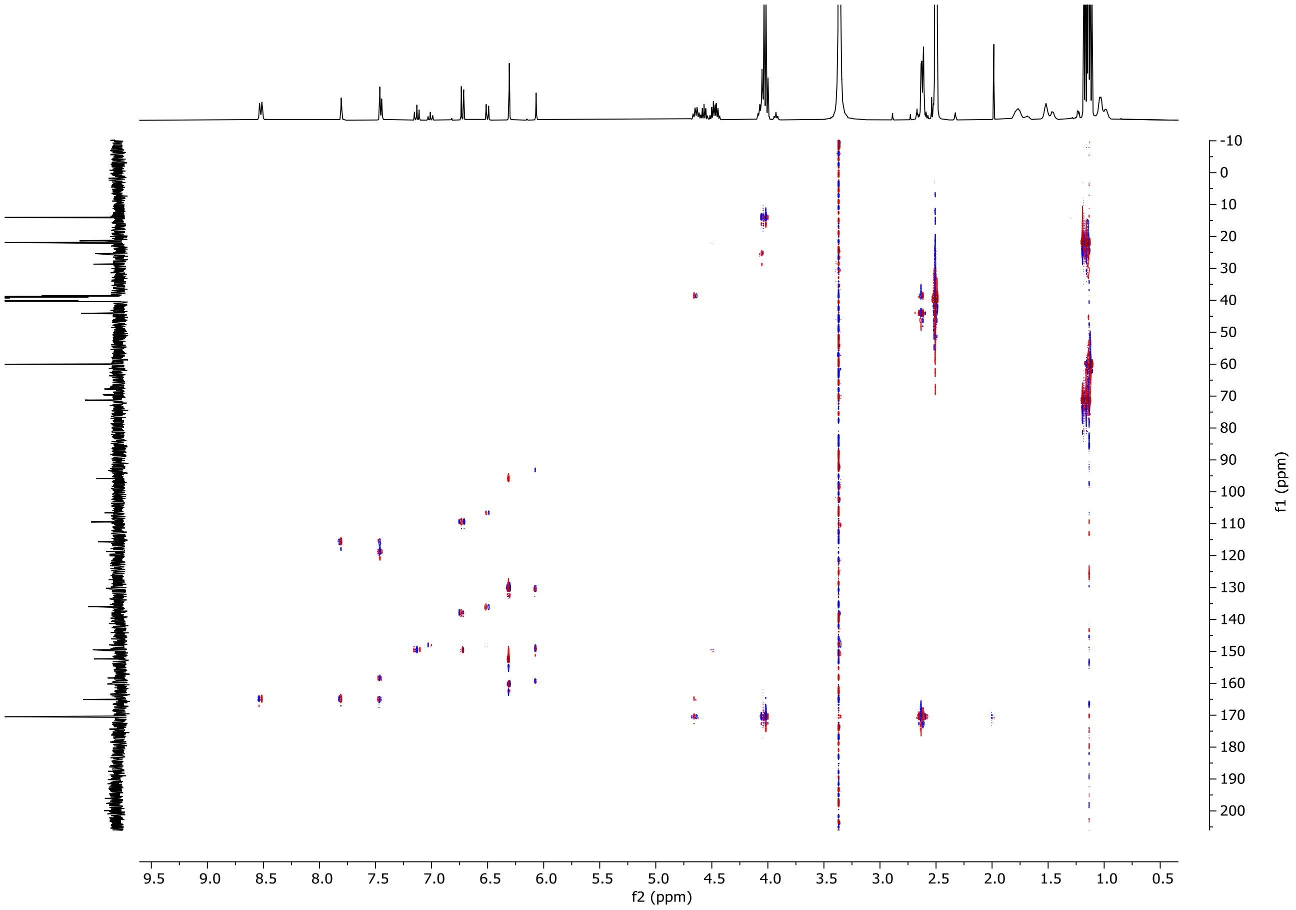

Figure S46: ^{1}H–^{13}C HMBC spectrum of compound **13** in DMSO-d_6 at 298 K.

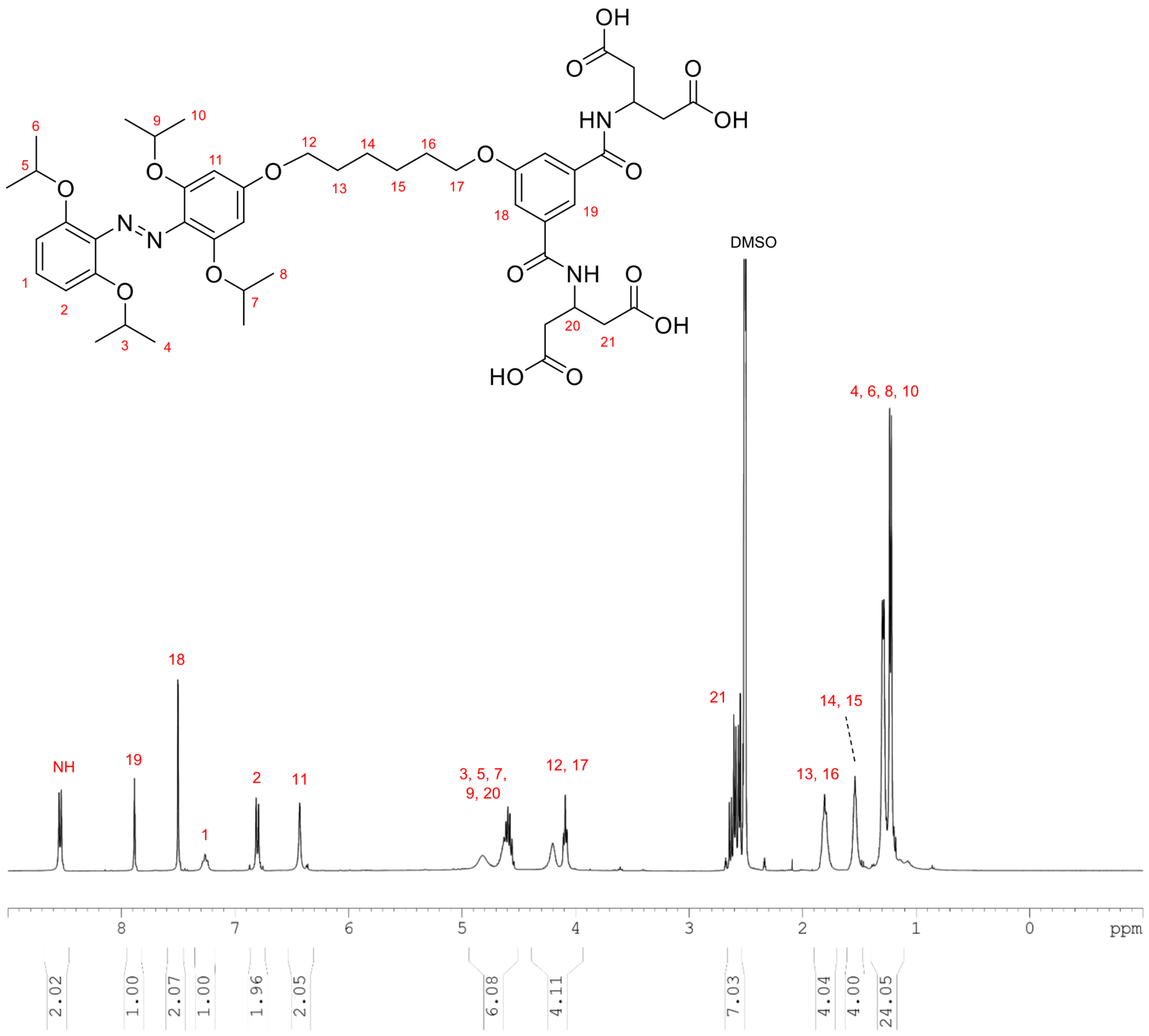

Figure S47: ^{1}H NMR spectrum of template **ipAzo** in DMSO-d_6 at 298 K.

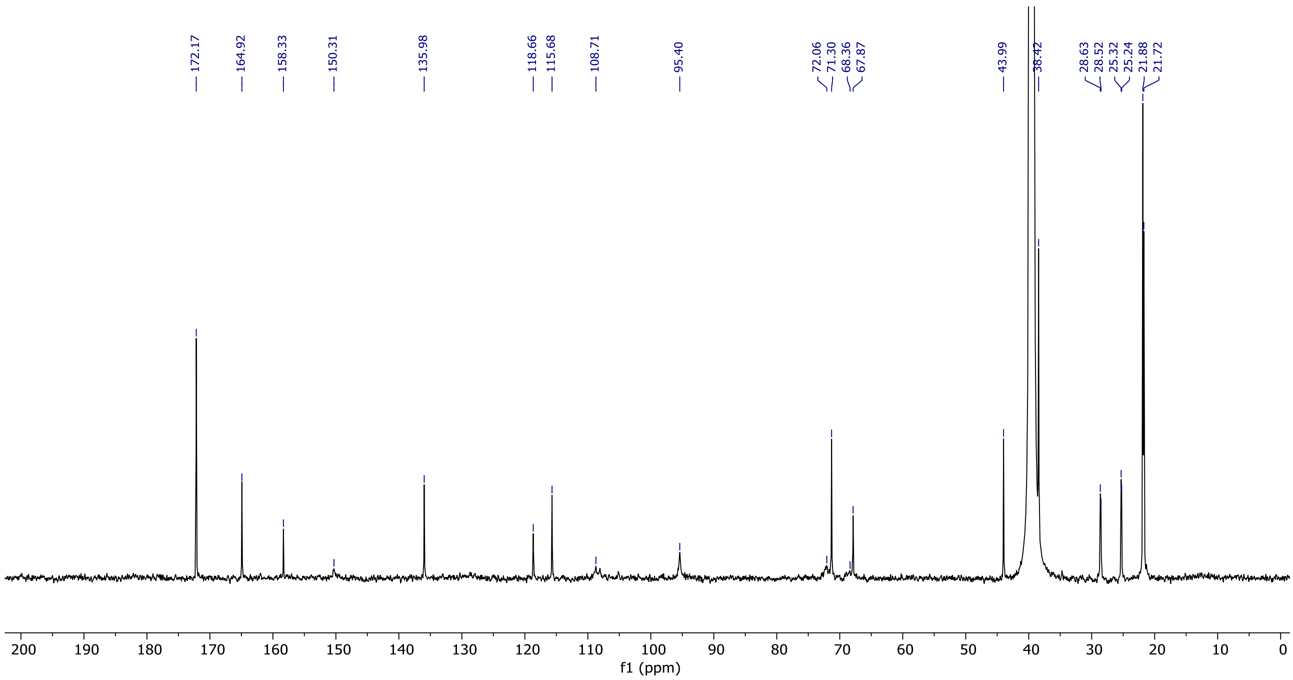

Figure S48: ^{13}C NMR spectrum of template **ipAzo** in DMSO-d_6 at 298 K.

S7. Supporting references

[S1] A. Erichsen, G. H. J. Peters and S. R. Beeren, *J. Am. Chem. Soc.*, 2023, **145**, 4882–4891.

[S2] D. Larsen and S. R. Beeren, *Chem. Sci.*, 2019, **10**, 9981–9987.

[S3] D. Wang, M. Wagner, A. K. Saydjari, J. Mueller, S. Minzen, H.-J. Butt and S. Wu, *Eur. J. Chem.*, 2017, **23**, 2628–2634.

[S4] M. J. Crossley, M. L. Fisher, J. J. Potter, P. W. Kuchel and M. J. York, *J. Chem. Soc. Perkin Trans. 1*, 1990, 2363–2369.

[S5] D. Wang, M. Wagner, H.-J. Butt and S. Wu, *Soft Matter*, 2015, **11**, 7656–7662.

[S6] K. Hirose, in *Analytical Methods in Supramolecular Chemistry*, ed. C. A. Schalley, Wiley-VCH Verlag GmbH & Co. KGaA, Weinheim, Second Edition, 2012, Chapter 2, pp. 27–66.

[S7] S. Meier and S. R. Beeren, *J. Am. Chem. Soc.*, 2014, **136**, 11284–11287.
